# The Role of Protein Side Chains in Enzyme-Activating Conformational Changes: Lessons from Studies on Variant Enzymes

**DOI:** 10.1021/acs.chemrev.5c00572

**Published:** 2025-10-20

**Authors:** Rania Hegazy, John P. Richard

**Affiliations:** † Department of Chemistry, 12292 University at Buffalo, SUNY, Buffalo, New York 14260-3000, United States

## Abstract

The active sites at the unliganded forms of many of Nature’s most proficient catalysts of metabolic reactions do not show a good fit for the enzymatic transition state; this fit is created by utilization of substrate binding energy to drive protein conformational changes that move side chains to positions that provide optimal transition-state stabilization. Static protein X-ray crystal structures of enzyme Michaelis complexes provide a critical starting point for determination of the roles of these side chains in stabilizing the enzymatic transition state but provide little insight into the catalytic role of the substrate-driven protein conformational change. Important elements of the mechanism of action of nature’s most proficient enzyme catalysts are therefore only revealed after examination of the structure for unliganded enzyme active sites and their substrate-driven transformations to structured forms that are complementary to reaction transition states. There have been few studies to determine the effect on enzyme activity of site-directed substitution of protein side chains that participate in substrate-driven enzyme conformational changes. The fascinating effects of these substitutions were probed by site-directed substitution of amino acid side chains that take part in conformational changes during catalysis by triosephosphate isomerase, glycerol phosphate dehydrogenase, and orotidine 5′-monophosphate decarboxylase.

## Graphical abstract

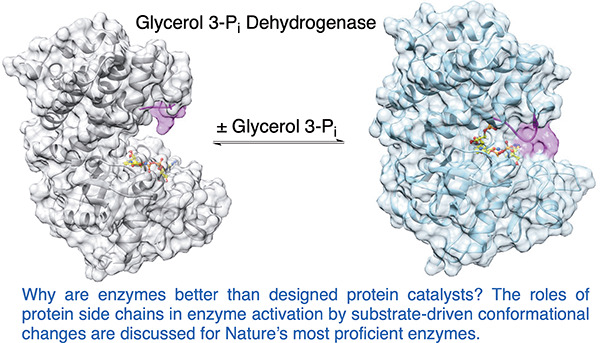

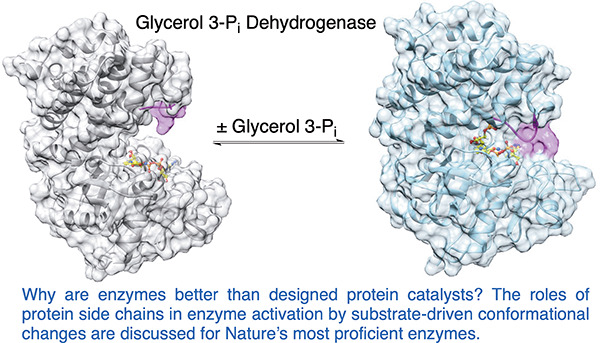

## 1 Introduction

The revolutionary methods for site-directed mutagenesis employing DNA polymerase and the primer extension method were developed by Hutchinson and Smith in 1978.^1^ This generated discussion about the potential and limitations of site directed mutagenesis as a tool for the determination of enzymatic reaction mechanisms. I first read in 1985 of the application of site-directed mutagenesis to triosephosphate isomerase (TIM), an enzyme whose mechanism I was working to understand.^2^ Motivated by X-ray crystallographic structural analyses and other results that implicated E165 as the basic active-site side chain that abstracts a proton from the enzyme-bound substrate,^3,4^ Knowles and co-workers reported the effect of an E165D substitution at TIM on the kinetic parameters for isomerization of glyceraldehyde 3-phosphate (GAP) to form dihydroxyacetone phosphate (DHAP).^5^ This side chain shortening resulted in a 1500-fold decrease in *k*_cat_ and an unexpected 3.6-fold decrease in *K*_m_ for wild-type TIM-catalyzed isomerization of GAP to form DHAP. Creative kinetic and mutagenesis studies on E165D TIM put to rest my doubts about the power of this method to shine light on enzymatic reaction mechanisms.^6,7^

Site-directed mutagenesis moved at lightning speed from an emerging technology to a routine tool for examining protein structure–function relationships. My grant proposals to use mutagenesis in studies on enzyme mechanisms drew comments that the experiments proposed might give uninterpretable results, complicated by unanticipated effects of the amino acid substitutions on protein structure.^8^ Such criticism emphasizes the importance of caution in the interpretation of experimental results. We have worked to obtain a large quantity of experimental data from studies on protein variants generated by substitutions of multiple active-site side chains, from substitution of multiple amino acids at a single position, and, from analyses of the effect of these substitutions on the kinetic parameters for several reactions catalyzed by a single enzyme. In some cases, it has been possible to examine the effects of substitutions on protein structure, and these effects have most often turned out to be minimal. In other cases, we have worked with the assumption that the substitutions have minimal effects on protein structure and then to justify this assumption by developing comprehensive, self-consistent and informative mechanistic models to rationalize large bodies of experimental data.

## 2 Substrate-Driven Protein Conformational Changes

### 2.1 One Key to Proficient Enzyme Catalysis

Many proficient enzyme catalysts, including TIM, undergo large substrate-driven conformational changes. We were struck by the lack of a general rationale for the many observations of induced-fit protein conformational changes.^9^ Our studies on enzyme mechanisms began in earnest at the start of this millennium and have focused on defining the role of phosphodianion-driven conformational changes in reactions catalyzed by three proficient and metabolically important enzymes: TIM, glycerol 3-phosphate dehydrogenase (GPDH) and orotidine 5′-monophosphate decarboxylase (OMPDC). Our seminal result was the demonstration that each protein catalyzes slow reactions of a phosphodianion truncated substrate piece and that each of these reactions is activated by up to 100,000-fold by the addition of 1.0 M of the missing phosphite dianion (HP_i_) piece.^10–13^ This was followed by the studies on variant enzymes reviewed here that focus on understanding the mechanism for enzyme activation from the utilization of binding energy from the whole phosphodianion substrate (Scheme 1A) or the HP_i_ piece (Scheme 1B) to drive a protein conformational change. This conformational change converts the floppy unliganded open enzyme **E**_**O**_ (*K*_C_ ≪ 1) to a tight and catalytically active closed protein **E**_**C**_, where the substrate is bound at an active-site cage that provides strong stabilization of the enzymatic reaction transition state.^14,15^

**Scheme 1:**
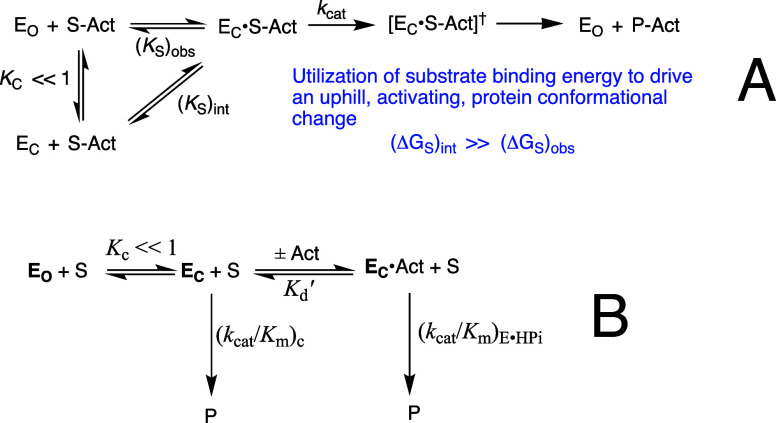
Substrate Induced-Fit Model for Enzyme Activation for Reaction of the Whole Substrate (S-Act) and Substrate Pieces (S + Act)

The explanation for enzyme-activation by these dianion-driven protein conformational changes now appears as obvious to us: the catalytic side chains at TIM, GPDH and OMPDC are initially scattered across the respective active sites of open enzymes **E**_**O**_ and are then locked into their active conformations by substrate binding that generates the active closed enzyme **E**_**C**_. Scheme 1A,B provide a starting point for rationalization of many more enzyme-activating substrate-driven conformational changes. These include the activation of a diverse set of enzyme-catalyzed reactions of truncated substrates **S** by activator pieces **Act** (Scheme 1B), where the activator is phosphite dianion derived from alkyl phosphates,^16^ the adenosine fragment derived from AMP,^17,18^ and the AMP or ADP fragments derived from the NAD^+^ cofactor.^19,20^

### 2.2 Thermodynamics for Protein Conformational Changes

The first reports of enzyme activation by substrate fragments appeared out of thin air, because the activating roles for these fragments had not been previously suggested by exhaustive examinations of X-ray crystal structures of enzyme-ligand complexes. For example, the structures of the **E**_**C**_ **•S-PO**_**3**_^**2–**^ Michaelis complexes of whole phosphodianion substrates or intermediate analogs for TIM and OMPDC, determined by X-ray crystallography, are typically used as the starting point in high-level calculations of the activation barriers to formation of enzyme-bound transition states.^21–26^ The substrate phosphodianion is treated as a spectator in these complexes, without consideration of the effect of the dianion on the catalytic activity of the open enzyme (**E**_**O**_). In fact, a low catalytic activity is expected for **E**_**O**_ relative to the closed enzyme (**E**_**C**_) because the active-site side chains are poorly placed to carry out their roles in catalyzing substrate turnover. Dianion activation would be minimal for Scheme 1B if the protein conformational change from inactive **E**_**O**_ to active **E**_**C**_ were thermoneutral. However, the extensive dianion-driven conformational changes for the enzymes reviewed in this article are best regarded as active collaborations between the protein and substrate in stabilization of protein active-site cages with structures complementary to that for enzymatic transition states. The large activation observed for enzyme-catalyzed reactions of truncated substrates (**S)** by the **Act** piece HPO_3_^2–^ is related to the barriers for the protein conformational change from **E**_**O**_ to **E**_**C**_ (Scheme 1B). For example, a 10,000-fold activation by 1.0 M phosphite dianion is consistent with *K*_C_ ≈ 10^–4^ and a ≈ 5–6 kcal/mol barrier for the conformational change from **E**_**O**_ to **E**_**C**_.

The induced-fit mechanism (Scheme 1A) did not evolve to provide for large second-order rate constants *k*_cat_/*K*_m_ compared to what is possible for catalysis by a closed rigid enzyme with the same structure and stabilizing transition state interactions.^27^ There are two important advantages for the utilization of substrate binding energy to drive the large activating protein conformational changes observed for catalysis by the most proficient enzymes.^28,29^

(1) Caged Michaelis complexes provide for optimal protein–ligand interactions, but at the same time occlude the substrate from binding directly to the closed form of the protein catalyst. Substrates therefore must bind to a second open protein conformer followed by the protein conformational change to form the active catalyst.

(2) The advantage for utilizing substrate binding energy to drive protein conformational changes so that (Δ*G*_S_)_int_ ≫ (Δ*G*_S_)_obs_ (Scheme 1A), compared to the direct expression of the full binding energy at the Michaelis complex, is that this avoids the full expression of large intrinsic substrate binding energies that would favor tight and effectively irreversible formation of the Michaelis complex.^30,31^

### 2.3 Roles for Active-Site Side Chains in Enzyme-Activating Conformational Changes

Metabolic and other enzymes operate by stabilizing the enzyme-bound transition state by direct interactions of protein side chains with the transition state. The magnitude of the interaction of an individual side chain may be estimated from the effect of its replacement with a noninteracting side chain on the enzyme kinetic parameter that is sensitive to changes in transition state stability. Other effects of side chain substitution almost invariably complicate the interpretation of experimental data. For example, protein side chains sometimes function to stabilize enzymes in their catalytically active closed forms (**E**_**C**_). These represent a second class of side chains. Substitution of an interacting side chain that stabilizes active **E**_**C**_ relative to **E**_**O**_ by a noninteracting side chain (Scheme 1) also results in a decrease in catalytic activity. This review will focus on our work to (i) Identify protein side chains that function to stabilize active **E**_**C**_ relative to inactive **E**_**O**_ protein conformations. (ii) Examine the effect of substitution of these side chains on enzyme kinetic parameters. (iii) Develop models to rationalize these effects.

Sections 3, 4, and 5 of this review each open with a brief discussion of the mechanism of action TIM, GPDH, or OMPDC, respectively, and then summarize the results of earlier traditional mutagenesis studies. This is followed by a description of mutagenesis studies that focus on characterizing the mechanism for each enzyme-activating protein conformational change. Substitutions of three types of side chains that participate in these conformational changes are described: (i) Side chains that stabilize the active closed enzyme **E**_**C**_ through direct protein-dianion interactions. These side chain interactions are prominent at the active site of GPDH and OMPDC. (ii) Side chains that stabilize the active closed form of the protein catalyst through interactions with other side chains. Examples of these side chains are presented for TIM, GPDH and OMPDC. (iii) Side chains whose movement during ligand-driven protein reorganization causes changes in the active-site microenvironment that favor formation of the reaction transition state. The ligand-driven clamping interactions of hydrophobic side chains with the E165 carboxylate in TIM are shown to play a critical role in the enhancing the basicity of the side chain that abstracts the substrate α-carbonyl proton.

## 3 Triosephosphate Isomerase (TIM)

### 3.1 Introduction

TIM catalyzes the reversible deprotonation of GAP (Scheme 2) by the carboxylate side chain of E165 to form an O-1 enediolate reaction intermediate.^32,33^ This intermediate undergoes intramolecular proton transfer mediated by H95 to form the O-2 enediolate followed by protonation to give the product DHAP.^33–35^ TIM is present in all known organisms and catalyzes isomerization with a second-order rate constant of close to the encounter-controlled limit.^36^

**Scheme 2:**
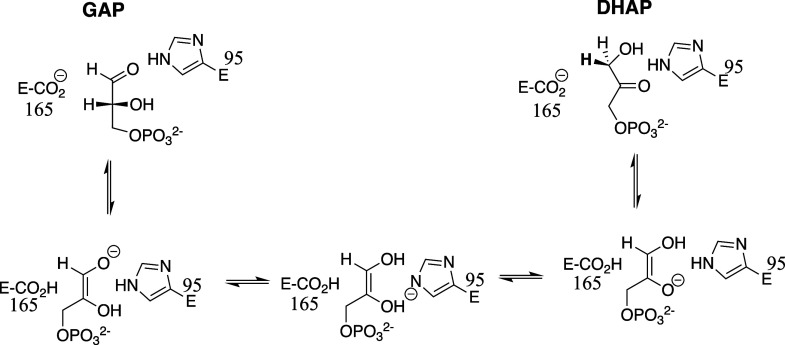
Mechanism for the Isomerization Reaction Catalyzed by TIM

There is a high conservation of amino acid side chains and of their positioning at the active sites of TIM from different organisms, but relatively little conservation in the sequence of amino acids at positions outside the active site. Consequently, there is only *ca* 14% sequence conservation at TIMs from different organisms.^37^ The high conservation of the structure of TIM active sites at enzymes from across the spectrum of evolved organisms suggests that TIM approached catalytic perfection as long as two billion years ago.^33–35^ We have worked with TIM from four different organisms; chicken (*c*TIM), yeast (*y*TIM), *Trypanosoma brucei brucei* (*Tbb*TIM), and *Leishmania mexicana* (*Lm*TIM), where the side chain numbering is identical for *c*TIM and *y*TIM, and for *Tbb*TIM and *Lm*TIM. We note the following small differences in the numbering of the amino acid residues that serve equivalent functions at the active sites of these different TIMs: (*c*TIM or *y*TIM and *Tbb*TIM or *Lm*TIM); N10/N11, K12/K13, E165/167, P166/168, I170/172, L230/232.

### 3.2 Early Mutagenesis Studies

There is an excellent review of the essential active-site side chains that are common to most, and in some cases, all TIM orthologues and a comprehensive summary of the results of studies of the effect of single side chain substitutions on enzyme activity.^37^ A second important review summarizes the results of many X-ray crystallographic structural determinations of TIM.^38^

The first mutagenesis studies on TIM targeted the E165,^5^ H95,^39,40^ and K12 ^41,42^ side chains that provide, respectively, the carboxylate base for substrate deprotonation, the neutral imidazole to provide hydrogen bonding stabilization of the enediolate reaction intermediate, and an alkyl ammonium cation to provide electrostatic stabilization of the enzyme-bound phosphodianion. The E165D substitution at *c*TIM results in a 420-fold falloff in *k*_cat_/*K*_m_ compared to wild-type TIM-catalyzed isomerization of GAP, which corresponds to a 3.6 kcal/mol destabilization of the isomerization reaction transition state. There are several small differences in the X-ray crystal structures for complexes of wild-type and E165D TIM to the enediolate analog phosphoglycolohydroxamate (PGH), the most notable being a 1 Å increase in the distance separating the side chain and enzyme-bound inhibitor.^43^

The S96 side chain is conserved in 98% of TIM sequences.^44^ The side chain sits close to the E165 carboxylate, and the Ser-OH forms a hydrogen bond to the E165 carboxylate in unliganded TIM. This hydrogen bond is lost upon binding of substrate or inhibitor to TIM, as the E165 carboxylate moves into position to deprotonate the acidic carbon of substrate.^43^ The S96P substitution results in a 27-fold falloff in *k*_cat_/*K*_m_ for wild-type *c*TIM, which corresponds to a 1.9 kcal/mol destabilization (ΔΔ*G*_GAP_^†^) of the isomerization reaction transition state. The S96P substitution at the E165D variant suppresses the effect of the E165D substitution and results in a 1.8 kcal/mol stabilization of the transition state (Figure 1A).^7^ The effect of the H95N substitution is likewise suppressed by the S96P substitution (Figure 1B).^7^ The G10S, S96T, E97D, V167F, and G223R substitutions are also second-site suppressors of the reduction in the catalytic activity for the E165D variant.^45^ The results indicate that the interactions of the E165 and D165 carboxylates, or the H95 and N95 side chains with the isomerization reaction transition state depend on neighboring active-site side chains and reflect differences in side chain packing at the congested active sites for variant TIMs (section 3.8).

**Figure 1:**
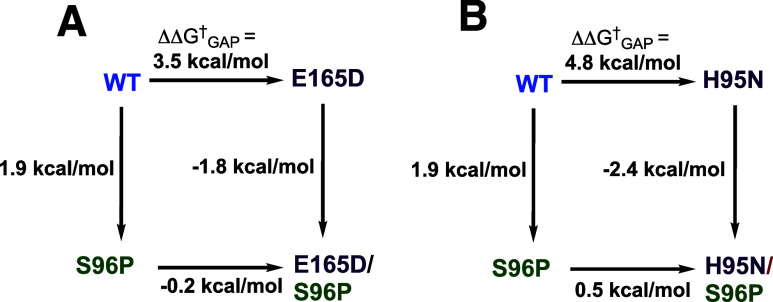
Variant cycles for the effect (ΔΔ*G*_GAP_^†^) of consecutive substitutions of amino acid side chains on the activation barrier Δ*G*_GAP_^†^ for TIM-catalyzed isomerization of GAP. The values of ΔΔ*G*_GAP_^†^ were calculated from the ratio of the values of *k*_cat_/*K*_m_ for the parent and variant enzyme-catalyzed reactions.

A comparison of X-ray crystal structures for wild-type TIM, the single S96P and E165D variants, and the double S96P/E165D variants shows that the substitutions give rise to small changes in active-site structure, but do not provide a full rationalization for the side chain effects on transition state stability illustrated by Figure 1A.^46^ These results are consistent with Arieh Warshel’s proposal that enzymes have evolved to optimize the precision in the placement of side chains at the Michaelis complex,^47,48^ so that small changes in the positioning of the catalytic side chains at the tightly packed active site for TIM, result in significant changes in the stability of the enzymatic transition state.

The neutral Nε2 of the H95 imidazole is positioned to form hydrogen bonds to DHAP and GAP substrate oxygens (Scheme 2). The results of studies on H95 variants of TIM provide strong evidence that these hydrogen bonds stabilize the enediolate reaction intermediates (Scheme 2).^39,40,49^ The H95 imidazole side chain also plays a complex role in facilitating proton transfer between the O-1 and O-2 intermediate oxyanions. Scheme 2 gives a hypothetical pathway for this proton transfer step^49^ that is consistent with results from studies by Mildvan and co-workers.^50,51^

The total 12 kcal/mol intrinsic phosphodianion binding energy for TIM was calculated from the ratio of the values of *k*_cat_/*K*_m_ for TIM-catalyzed reactions of the whole substrate GAP and of the truncated substrates glyceraldehyde or glycolaldehyde (GA, see Scheme 3).^11,52^ The dianion binding energy is due mainly, or entirely, to interactions of the dianion with G171, S211, G232, and G233 backbone amides and with the cationic side chain of K12 that forms a solvent-separated ion pair with the ligand phosphodianion.^53,54^ The X-ray structure of the K12M/G15A TIM variant, determined for crystals grown in the presence of 1.5 mM of the tight binding inhibitor PGH closely resembles the open structure for unliganded wild-type TIM. The enzyme is present in the open conformation **E**_**O**_ and there is no bound inhibitor.^42^ The interactions between the K12 side chain and enzyme-bound inhibitor therefore stabilize the active closed enzyme **E**_**C**_ relative to **E**_**O**_. This stabilization represents a tightening of ionic interactions between the K12 cation and the ligand dianion that occurs as the effective active-site dielectric constant is reduced on moving from the aqueous environment for open **E**_**O**_ to the organized nonaqueous environment for closed **E**_**C**_.^29^

**Scheme 3:**
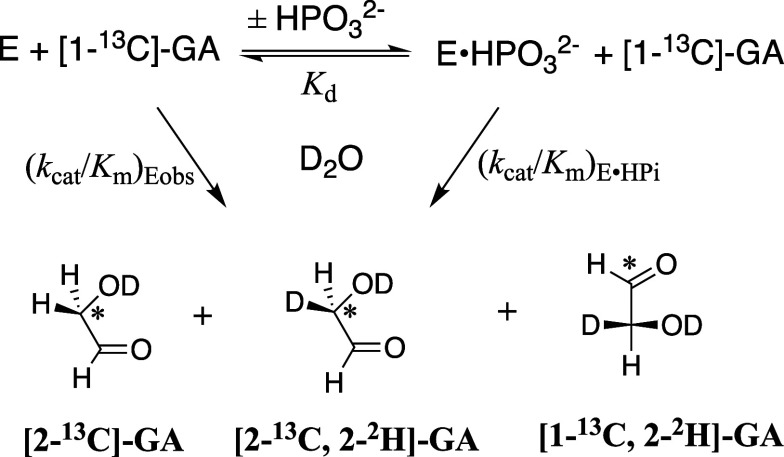
Unactivated and Phosphite Dianion Activated TIM-Catalyzed Reactions of [1-^13^C]-GA in D_2_O Where the ^13^C-Labeled Carbon is Shown by an Asterisk

The interactions between the K12 side chain and the transition state for TIM-catalyzed isomerization were characterized in studies on the effect of K12M, K12M/G15A,^41,42^ and K12G^55,56^ substitutions at *y*TIM on enzyme activity. The K12M variant of *y*TIM has no detectable activity,^41^ while the K12G substitution results in a 600,000-fold decrease in *k*_cat_/*K*_m_ for *y*TIM-catalyzed isomerization of DHAP and the elimination of phosphite dianion-activated TIM-catalyzed reactions of GA observed for wild-type TIM.^55^ The results show that the transition state for TIM-catalyzed isomerization of GAP and for phosphite dianion activated deprotonation of GA are strongly stabilized interactions with the K12 side chain cation. This is divided for the whole substrate reaction between a 2–3 kcal/mol stabilization of the Michaelis complex to substrate and a *ca* 5–6 kcal/mol stabilization that is expressed specifically at the isomerization reaction transition state.^55^ The placement of the K12 side chain at the surface of TIM (see Figure 5), and a second ion pair interaction between the K12 cation and the E97 side chain anion facilitates the efficient rescue of the K12G variant by alkyl ammonium cations.^56^ The effect of substitutions of the E97 side chain on enzyme activity are discussed in section 3.8.^57,58^

### 3.3 Catalysis by TIM: Better Because it is Different

An interesting conclusion from early mutagenesis studies on TIM was that enzymatic catalysis of isomerization was “*not different just better*” than nonenzymatic catalysis.^34^ A comparison of the roles of the active-site TIM side chains with those for small buffer catalysts in water provides justification for this statement.^2,59^ At the same time the statement that catalysis by TIM somehow approaches perfection without being different is confusing. Freely diffusing buffer acids and bases in water are unstructured in comparison to the structured placement of catalytic side chains at the active site of TIM that is only achieved after a complex substrate-driven protein conformational change.^53,54,60^ Including this conformational change in the catalytic cycle for TIM-catalyzed isomerization represents a profound difference from the reactions of freely diffusing substrate and catalyst in water.^27,31^

The prime imperative for efficient catalysis of deprotonation of the weakly acidic α-carbonyl carbon of DHAP is to reduce the reaction activation barrier by reducing the large thermodynamic barrier to formation of an enediolate reaction intermediate.^61,62^ The results of high-level empirical valence bond (EVB) calculations (Figure 2)^63^ of the free-energy reaction profiles for nonenzymatic deprotonation of DHAP (p*K*_a_ = 18)^2^ by propionate anion (p*K*_a_ = 4.9)^64^ and the profile for deprotonation of DHAP bound to TIM provide strong evidence that this imperative is satisfied for substrate deprotonation at the active site of TIM. The profiles show that interactions between TIM and the bound substrate effect a 13 kcal/mol reduction in Δ*G* ^0^ for substrate deprotonation and a smaller 10 kcal/mol reduction in the activation barrier Δ*G*_f_^†^ compared to the corresponding barriers for substrate deprotonation by propionate anion in water.

**Figure 2:**
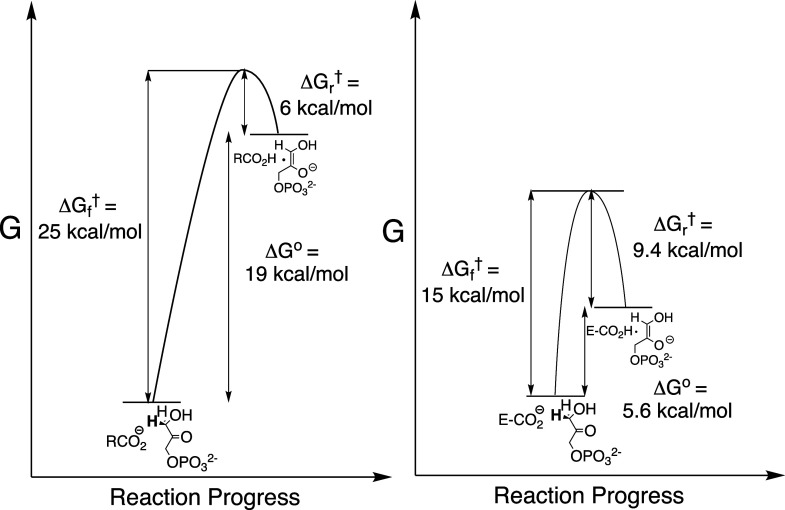
A comparison of the free-energy reaction profiles for deprotonation of DHAP by propionate anion in water (left) and the computed free energy profile for deprotonation of TIM-bound DHAP by the carboxylate side chain of E165 (right). The binding of DHAP to TIM reduces Δ*G*_f_^†^ and Δ*G* ^0^ by 10 and 13.4 kcal/mol, respectively.^23,63^

The preorganization of side chains at the active site of TIM cannot cause a decrease in the large thermodynamic barrier to deprotonation of the weakly acidic α-carbonyl substrate carbon.^62,65^ Catalysis by TIM is enhanced compared to catalysis by small molecules in water by the utilization of binding energy from the substrate phosphodianion to drive a protein conformational change that creates an environment at the enzyme active site where substrate deprotonation is strongly favored in comparison to deprotonation in water (Figure 2). In other words, part of the substrate dianion binding energy is invested to drive a protein conformational change, and the investment is returned by creation of an active site that strongly favors substrate deprotonation to form the enediolate intermediate. We have focused on determining the specific features of the closed form of TIM that favor formation of the reaction intermediate.

### 3.4 Kinetic Parameters for TIM-Catalyzed Reactions of Substrate Pieces

Original insight into the role of the dianion-driven protein conformational change in reducing the thermodynamic barrier to deprotonation of TIM-bound substrate (Figure 2) has been obtained in studies on the mechanism for activation of TIM-catalyzed deprotonation of GA by phosphite dianion. The first step in determining the effect of site-directed substitutions of TIM on the enzyme-catalyzed reactions of the substrate pieces GA + HP_i_ was the development of analytical methods to quantify the yields of reaction products. We first observed that TIM catalyzes the slow exchange from D_2_O into the C-2 carbon of GA to form [2-^2^H]-GA by either a direct deuterium exchange reaction or by the degenerate isomerization reaction that also proceeds with deuterium exchange.^11^ These TIM-catalyzed reactions of GA are strongly activated by phosphite dianion.^11^ We then examined the TIM-catalyzed isomerization and deuterium exchange reactions of [1-^13^C]-GA in D_2_O (Scheme 3) and quantified the yields of the following three isotopomer products (Scheme 3) using ^1^H NMR; (i) [2-^13^C]-GA from isomerization of [1-^13^C]-GA; [2-^13^C, 2-^2^H]-GA from isomerization of [1-^13^C]-GA with deuterium exchange. (iii) [1-^13^C, 2-^2^H]-GA from direct deuterium exchange at carbon-2 of [1-^13^C]-GA.^14^ All three TIM-catalyzed reactions are strongly activated by phosphite dianion, but the dianion has little or no effect on the fractional product yields and, likely, no significant effect on the position of GA relative to the enzyme catalytic side chains.^14^ There is a *ca* 20% yield of the dideuterium labeled product [1-^13^C, 2,2-^2^H]-GA from the unactivated TIM-catalyzed reaction of [1-^13^C]-GA. This was attributed to the reversible reaction of surface lysine amines to give an iminium cation that undergoes rapid deuterium exchange.^66^ The yield of [1-^13^C, 2,2-^2^H]-GA falls off sharply for reactions in the presence of phosphite dianion activator, because there is no dianion activation of the deuterium exchange reaction mediated by surface lysines.

1 $$K_{d}^{†}=\frac{K_{d}{\left(k_{\mathrm{cat}}/K_{m}\right)}_{E}}{{\left(k_{\mathrm{cat}}/K_{m}\right)}_{E•\mathrm{HPi}}}$$

2 $$\Delta G_{\mathrm{HPi}}^{†}=-\mathrm{RTln}\left(1/K_{d}^{†}\right)$$

We typically report the following kinetic parameters for wild-type and variant TIM-catalyzed reactions of the substrate pieces [1-^13^C]-GA and HP_i_ in D_2_O (Scheme 4). (i) The second-order rate constant (*k*_cat_/*K*_m_)_E_ for the unactivated TIM-catalyzed reactions of [1-^13^C]-GA to form the reaction products from Scheme 3 is calculated as (*k*_cat_/*K*_m_)_E_ = f_E_ (*k*_cat_/*K*_m_)_obs_ where *f*_E_ = 0.80 is the fractional yield of the three products from reactions at the wild-type enzyme active site. The value of *f*_E_ decreases for substitutions that reduce the velocity of product formation from active-site reactions (Scheme 3) without affecting the velocity for formation of [1-^13^C, 2,2-^2^H]-GA from deuterium exchange mediated by surface lysines. (ii) The third-order rate constant [(*k*_cat_/*K*_m_)_E•HPi_/*K*_d_] for phosphite dianion activated TIM-catalyzed reactions of [1-^13^C]-GA. (iii) The dissociation constant *K*_d_^†^ (eq 1) for release of phosphite dianion from the transition state complex for TIM-catalyzed reactions of [1-^13^C]-GA. This dissociation constant defines the intrinsic phosphite dianion binding energy Δ*G*_HPi_^†^ (eq 2).

**Scheme 4:**
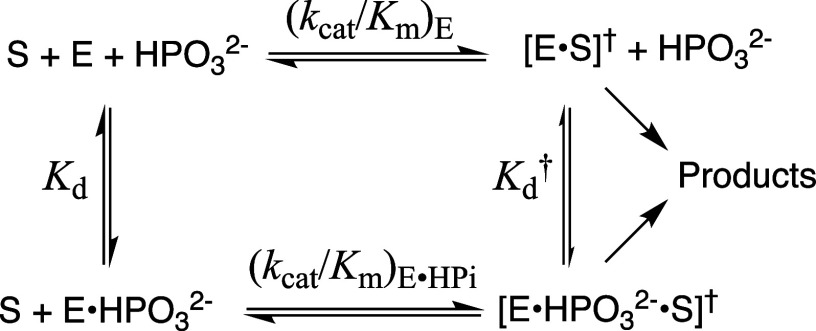
Mechanism for Phosphite Dianion Activated Enzyme-Catalyzed Reactions of Substrate Pieces S + HP_i_, where S = GA for TIM and GPDH, and EO or FEO for OMDC (Scheme 15)

### 3.5 Loops 6 and 7

The roles of TIM barrel loops 6 and 7 in TIM-catalyzed reactions of whole substrate and substrate pieces have been probed extensively in mutagenesis studies. Wierenga and co-workers provide a lucid description of the motions of loops 6 and 7 that are driven by interloop and protein-phosphodianion interactions.^67^ Most striking is the *ca* 10 Å movement of rigid loop 6 residues [169-AIGTG-173] about N-terminal [166-PVW-168] and C-terminal [174-KTA-176] hinges, to cover the ligand dianion and form a hydrogen bond between the dianion and backbone amide of G171 (Figure 3).^54^ This motion of loop 6 is coupled to 90° and 180° rotations, respectively, in the planes defined by the peptide bonds for the G209 and G210 side chains at loop 7 that give rise to the following intra- and interloop hydrogen bonds (Figure 3):^67^ (A) Interloop hydrogen bonds between; (i) the backbone amide NH of Gly-173 and the γ-OH of S211, (ii) The backbone amide NH of A176 and the phenol oxygen of Y208 and, (iii) the carbonyl amide oxygen of A169 and the γ-OH of S211. (B) An intraloop hydrogen-bond between the backbone amide NH of G173 and the carbonyl amide oxygen of A169 (not shown).

**Figure 3:**
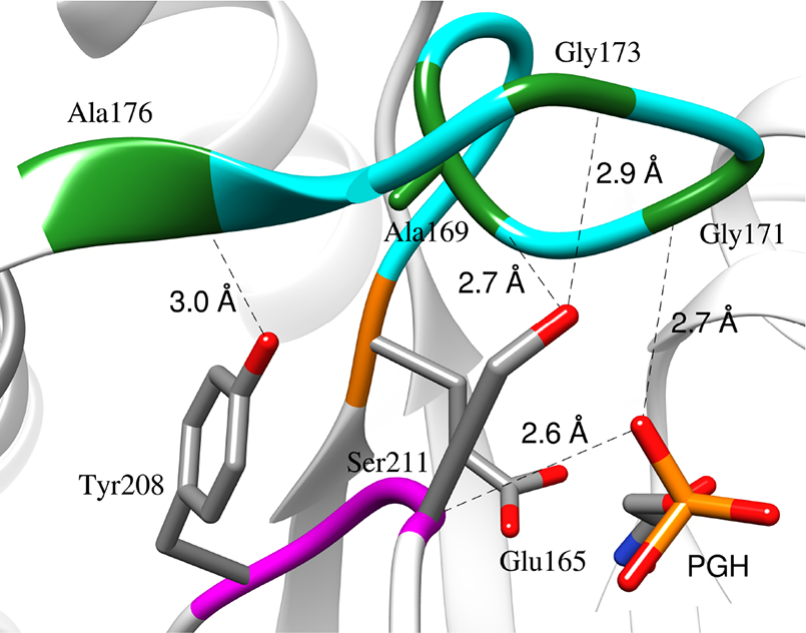
A representation of the structure of the closed form of *c*TIM liganded with PGH (PDB 1TPH][Bibr ref68] with the following hydrogen bonds between: The backbone amide from G171 in loop 6 (blue and green colors) and the substrate phosphodianion; backbone amides from loop 6 and the Y208 and S211 side chains from loop 7 (magenta). Reproduced with permission from ref 69. Copyright 2015 American Chemical Society.

#### 3.5.1 Substitutions and Deletions at Loop 6

The loop 6 deletion variant (L6DM) of wild-type *c*TIM (Figure 4) was prepared by deletion of the 170–173 tip residues (Figure 4) and introduction of a new peptide bond between A169 and K174. This eliminates the hydrogen bond between the substrate phosphodianion and G171 and exposes the enzyme active site to bulk solvent (Figure 5). The L6DM causes 10^6^- and 10^5^-fold decreases, respectively, in the kinetic parameters *k*_cat_/*K*_m_ and *k*_cat_ for isomerization of GAP,^70,71^ that correspond to an 8 kcal/mol destabilization of the isomerization reaction transition state relative to reactants in water, a 7 kcal/mol destabilization of this transition state relative to the substrate Michaelis complex but only a 1 kcal/mol destabilization of the Michaelis complex. The L6DM eliminates phosphite activation of TIM-catalyzed deprotonation of GA,^71^ and strongly promotes the TIM-catalyzed elimination reaction of GAP to form methylglyoxal and phosphate.^70^ This elimination is the dominant nonenzymatic reaction in water and is strongly suppressed at TIM by phosphodianion interactions with loop 6,^2,70,72^ and the K12 side chain.^55^ The TIM-catalyzed elimination reaction of substrate is the only documented enzymatic source of methylglyoxal in mammalian tissues.^72,73^

**Figure 4:**
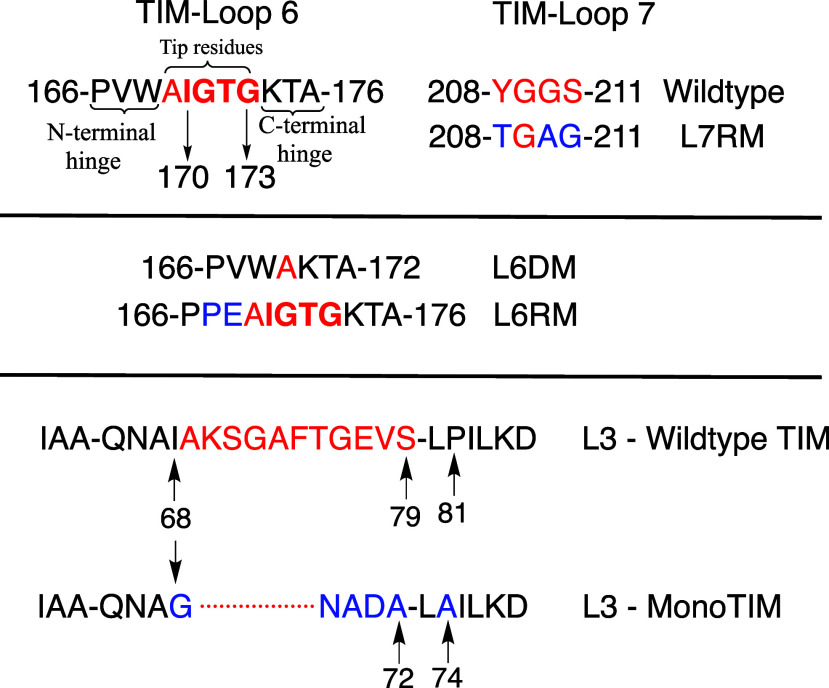
(top) Sequences for loop 6 and loop 7 for wild-type TIMs from eukaryotes, and for the loop 7 replacement variant L7RM obtained by substituting wild-type loop 7 side chains by side chains for TIM from a*rchaebacteria*.^74^ (middle) Sequence for the loop 6 deletion (L6DM)^70^ and loop 6 replacement variants (L6RM). The later was prepared by substituting wild-type loop 6 side chains by the side chains for the enzyme from *archaebacteria*.^74^ (bottom) A comparison of the sequence for loop 3 at *Tbb*TIM and loop 3 at an engineered monomeric variant (section 3.9.)^75^

**Figure 5:**
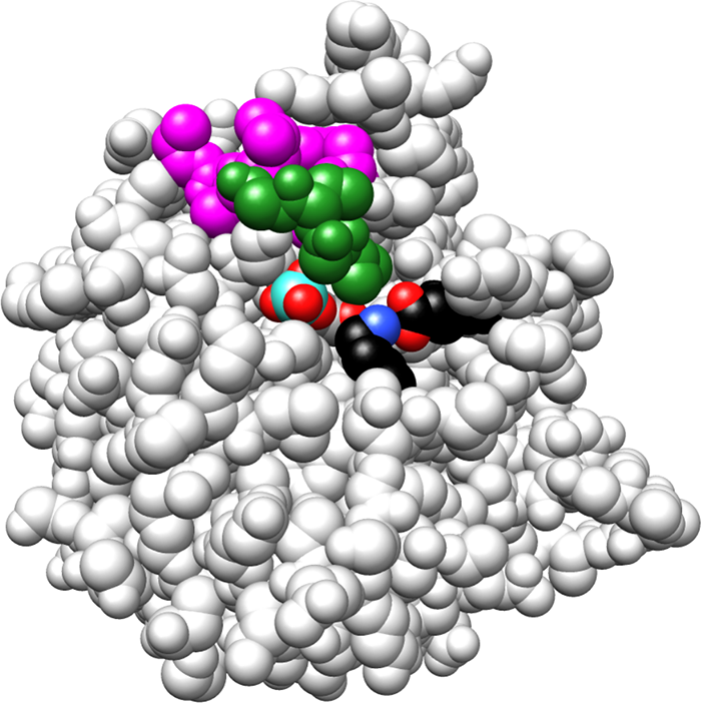
Space-filling model of the complex between TIM from yeast and PGA [PDB 2YPI].^54^ The amino acid residues retained and deleted for the L6DM are colored, respectively, magenta and green so that the L6DM exposes the enzyme active-site to solvent water. The K12 side chain has the cationic nitrogen (blue) in an ion pair to the carboxylate side chain of E97 (red). Reproduced with permission from ref 32. Copyright 2012 American Chemical Society.

The 1 kcal/mol effect of the L6DM on *K*_m_ is consistent with only weak interactions between the substrate and deleted side chains at the Michaelis complex, and the total 8 kcal/mol effect on the stability of the isomerization reaction transition state is larger than expected for elimination of the single hydrogen bond between loop 6 and the substrate dianion. However, the interactions between loop 6 and the substrate trap the substrate/intermediate at a hydrophobic active site of low effective dielectric constant *D*, an environment that strongly enhances transition state stabilization by electrostatic interactions that are proportional to 1/*D*.^29^

The effects of substitution of C- and N-terminal hinge side chains from loop 6 (Figure 4) on *c*TIM-catalyzed isomerization were examined by Sampson and co-workers.^76–81^ A library of 20 ^3^ N-terminal hinge sequences was prepared by random codon substitution. A number of sequences gave active TIMs, but only three active variants contain even a single hinge glycine.^81^ The [166-P**GGA**IGTG**GGG**-176] variant in which five of the six loop 6 hinge residues are replaced by glycine has a 2500-fold smaller *k*_cat_ and a 10-fold larger *K*_m_ compared to wild-type TIM.^77^ An NMR study on this variant provides evidence that the five Gly side chains introduce a large conformational heterogeneity into loop 6.^76^ This suggests that the low activity for the variant is partly or entirely due to the large entropic barrier to freezing motions at the five Gly residues that occurs when loop 6 closes over substrate to form the active closed enzyme **E**_**C**_.

A loop 6 replacement variant (L6RM) of TIM was prepared by substituting the N-terminal hinge sequence at wild-type TIM from eukaryotes/bacteria [166-PXW-168 (X = L, V)] by the *archaebacteria* sequence (166-PPE-168, Figure 4).^78^ This substitution results in a 23000-fold decrease in *k*_cat_/*K*_m_ for isomerization, a 5400-fold decrease in (*k*_cat_/*K*_m_)_E•HPi_/*K*_d_) for phosphite dianion activation of TIM-catalyzed reactions of [1-^13^C]-GA (Scheme 4),^71^ an increase in *K*_m_ from 2.9 × 10^–4^ M to ≫ 0.04 M for isomerization of GAP, and elimination of enzyme inhibition by 10 mM PGA, for which *K*_i_ = 1.9 × 10^–5^ M for wild-type TIM.^71^ The X-ray crystal structure for the unliganded L6RM shows a large *ca* 5 Å displacement of the E168 hinge side chain from the position at unliganded wild-type TIM.^71^ The side chain displacement is due to substitution of a stabilizing hydrogen bond between the W168 (loop 6) and E129 side chains at wild-type *c*TIM by destabilizing electrostatic interactions between the E168 and E129 side chain anions at the L6RM. The formation of the active closed form of the L6RM now requires the close approach of anionic E129 and E168 side chains that is driven by utilization of the large intrinsic substrate dianion binding energy. The very large values of *K*_m_ and *K*_i_, respectively, for isomerization of GAP and inhibition by PGA for the L6RM provide an informative example of a TIM variant that utilizes essentially all of the large phosphodianion binding energy to drive the now strongly unfavorable enzyme conformational change associated with formation of the electrostatically stressed, catalytically active, Michaelis complex (**E**_**C**_ **•S-Act**, Scheme 1) so that minimal binding energy is expressed in the observed parameters *K*_m_ or *K*_i_.^71^

#### 3.5.2 Substitutions at Loop 7

The TIM active-site cage is stabilized by hydrogen-bonding interactions of the phenol oxygen of Y208 with the backbone amide NH of A176 from loop 6 and of the γ-OH of S211 with the backbone amide NH of A176 and the carbonyl oxygen of A169 from loop 6 (Figure 3). Substitutions of Y208 and S211 from loop 7 weaken interloop interactions and result in falloffs in the values of *k*_cat_ and *k*_cat_/*K*_m_ for TIM-catalyzed reactions of the whole triosephosphate substrate.^82,83^ We revisited this problem and examined the effect of Y208 and S211 substitutions on the kinetic parameters for TIM-catalyzed reactions of the substrate pieces [1-^13^C]-GA and HP_i_.^69,84^ The results are summarized in Table 1 where (i) (*k*_cat_/*K*_m_)_E_ is the rate constant for the unactivated TIM-catalyzed reactions of [1-^13^C]-GA (Scheme 4). This is the sum of the rate constants for the reactions catalyzed by the closed (**E**_**C**_) and open (**E**_**O**_) forms of TIM (eq 3, derived for Scheme 5). The results from an EVB computational study provide support for the proposal that the unactivated reaction of GA occurs exclusively at **E**_**C**_ so that *f*_Ec_ (*k*_cat_/*K*_m_)_E_c__ ≈ (*k*_cat_/*K*_m_)_E_.^85^ (ii) (*k*_cat_/*K*_m_)_E•HPi_/*K*_d_ is the third-order rate constant for the phosphite dianion-activated TIM-catalyzed reaction of [1-^13^C]-GA (Scheme 4); and (iii) Δ*G*_HPi_^†^ is the intrinsic phosphite dianion binding (eq 2, for Scheme 4).

3 $${\left(k_{\mathrm{cat}}/K_{m}\right)}_{E}=f_{\mathrm{Ec}}{\left(k_{\mathrm{cat}}/K_{m}\right)}_{\mathrm{Ec}}+f_{\mathrm{Eo}}{\left(k_{\mathrm{cat}}/K_{m}\right)}_{\mathrm{Eo}}$$

**Table 1:** Kinetic Parameters for Wild-Type and Variant TIM-Catalyzed Reactions of the Whole Substrate GAP and the Substrate Pieces [1-^13^C]-GA and Phosphite Dianion^69^

enzyme	(*k*_cat_/*K*_m_)_GAP_ (M^–1^s^–1^) (*k*_cat_)_GAP_ (s^–1^)	ΔΔ*G* ^†^ ^a^ (kcal/mol)	(*k*_cat_/*K*_m_)_E_ (M^–1^s^–1^)^b^	$\frac{{\left(k_{\mathrm{cat}}/K_{m}\right)}_{E•\mathrm{HPi}}}{K_{d}}$ (M^–2^s^–1^)^c^	Δ*G*_HPi_^†^ ^d^(kcal/mol)	ΔΔ*G*_HPi_^†^ (kcal/mol)
wild-type^e^ 208-YGGS-211	8.9 × 10^6^ 8900		0.062	2700	–6.3	
Y208T^e^	1.1 × 10^6^ 3700	1.2	0.065	390	–5.1	1.2
Y208S^e^	2.4 × 10^5^ 940	2.1	0.071	78	–4.1	2.2
Y208A^e^	2.6 × 10^5^ 740	2.1	0.050	63	–4.2	2.1
Y208F^e^	5.4 × 10^3^ 13	4.4	0.003	2.1	–3.9	2.4
S211A^e^	2.3 × 10^5^ 2800	2.2	0.002	79	–6.3	0.0
S211G^e^	4.2 × 10^6^ 7500	0.4	0.13	2900	–5.9	0.4
Y208T/S211G^e^	7.3 × 10^5^ 520	1.5	0.12	600	–5.0	1.3
L7RM^f^ 208-TGAG-211	5.9 × 10^4^ 16	3.0	0.0045	95	–5.9	0.4
L6RM^f^	470	6.2	nd	0.5		

a *RT* ln­[(*k*_cat_/*K*_m_)_WT_/(*k*_cat_/*K*_m_)_var_)].

b Equation 3.

c Scheme 4.

d Equations 1 and 2.

e *y*TIM.

f *c*TIM.

**Scheme 5:**
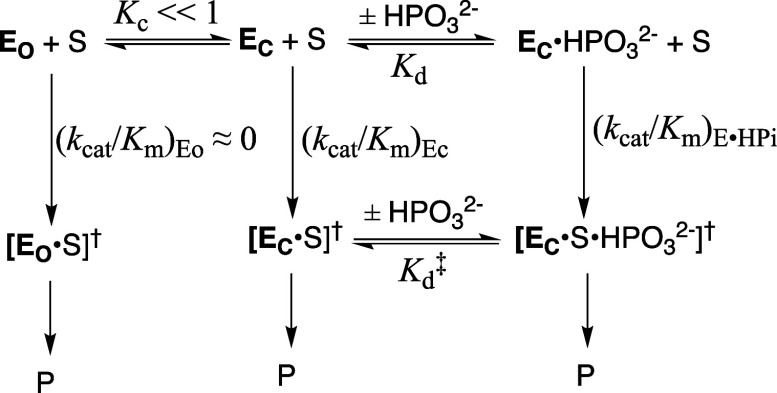
Kinetic Mechanism for Unactivated and Phosphite Dianion Activated Reactions of a Dianion Truncated Substrate Where (*k*_cat_/*K*_m_)_Eo_ ≈ 0 ^85^

A logarithmic plot of third-order rate constants (*k*_cat_/*K*_m_)_E•HPi_/*K*_d_ for dianion activated wild-type and variant TIM-catalyzed reactions of GA against (*k*_cat_/*K*_m_)_GAP_ for the TIM-catalyzed reactions of whole substrates is linear with a slope of 1.04.^69,84^ This plot demonstrates that these amino acid substitutions cause the same changes in the activation barrier for TIM-catalyzed reactions of substrate pieces and whole substrate and that the two reaction transition states are stabilized by similar interactions with the substituted side chains.^69,84^ The results provide support for the conclusion that the dianion serves a similar role, in catalysis of the reactions of whole substrate and substrate pieces, of providing binding energy to hold TIM in the active closed conformation **E**_**C**_. The similar linear logarithmic correlations of kinetic parameters observed for reactions of whole substrate and substrate pieces catalyzed by wild-type enzyme and variants of GPDH (section 4.4.2) and OMPDC are consistent with similar roles for the dianion in providing binding energy to stabilize catalytically active protein cages.^86–89^ The results of an EVB computational study to model TIM-catalyzed deprotonation of GA showed that binding of phosphite dianion to the binary E•GA complex results in no significant change in Δ*G* ^†^ for carbon acid deprotonation. This provides support for the conclusion that the dianion functions at the ternary E•GA•HP_i_ complex solely to hold TIM in the active closed conformation.^85^

A second important observation is that substitutions of the Y208 side chain, which does not interact directly with the enzyme-bound dianion (Figure 3), cause up to a 2.4 kcal/mol decrease in the intrinsic dianion binding energy Δ*G*_HPi_^†^. The interactions of this large hydrophobic side chain with loop 6 therefore enable the development of stabilizing interactions between the protein and the dianion, presumably by stabilizing a protein cage with low effective dielectric constant that provides for optimal stabilizing electrostatic interactions. We note several additional interesting observations from Table 1.

(1) The S211A substitution has only a small effect on the intrinsic dianion binding energy Δ*G*_HPi_^†^, but causes the values for (*k*_cat_/*K*_m_)_GAP_, (*k*_cat_/*K*_m_)_E_ and (*k*_cat_/*K*_m_)_E•HPi_/*K*_d_ to decrease. The 30-fold decreases in (*k*_cat_/*K*_m_)_E_ for the unactivated reaction of GA and in (*k*_cat_/*K*_m_)_E•HPi_/*K*_d_ for the dianion activated reaction are consistent with a decrease in *K*_C_ for the protein conformational change (Scheme 1B) due to the destabilization of **E**_**C**_ relative to **E**_**O**_ (Scheme 5) from loss of hydrogen bonds from the S211 side chain to the backbone amide NH of G173 and the carbonyl oxygen of A169 (Figure 3). The proposed decrease in the concentration of the active enzyme **E**_**C**_ would also give rise to an increase in dianion binding energy utilized to drive the protein conformational change that is reflected by the 12-fold larger value of *K*_m_ for the S211A variant compared to wild-type *y*TIM (Scheme 1A). By comparison the S211G substitution in wild-type TIM causes only small changes in (*k*_cat_/*K*_m_)_GAP_, (*k*_cat_/*K*_m_)_E_ and (*k*_cat_/*K*_m_)_E•HPi_/*K*_d_ for the parent enzyme, perhaps because water can now substitute for the excised Ser hydroxyl to stabilize the loop-closed enzyme.

(2) The effect of the Y208F substitution on Δ*G*_HPi_^†^ (2.4 kcal/mol) is similar to that for Y208 S and A substitutions. However, Y208F causes a 4.4 kcal/mol increase in the barrier to (*k*_cat_/*K*_m_)_GAP_ for isomerization of GAP that is large in comparison to the effects of Y208 T, S and A substitutions, and, a 1.8 kcal/mol increase in the barrier for (*k*_cat_/*K*_m_)_E_. The results are consistent with a specific effect of the Y208F substitution on protein structure that reduces the reactivity of **E**_**C**_ toward catalysis of the reaction of both GAP, and the GA and HP_i_ pieces. There is evidence, from earlier work, that the Y208F substitution causes an increase in the barrier for a rate-determining protein conformational change.^82^

(3) The G210A substitution at the Y208T/S211G variant gives the TGAG sequence for loop 7 in *archaebacteria*; the overall TGAG for YGGS substitution at *c*TIM corresponds to the loop 7 replacement mutation (L7RM, Figure 4). The L7RM at *c*TIM results in a small 0.4 kcal/mol decrease in Δ*G*_HPi_^†^ (eq 2) for dianion activation. The loop substitution causes a large decrease in the intrinsic reactivity of the TIM variant that gives rise to 150-, 14- and 28-fold decreases, respectively, in (*k*_cat_/*K*_m_)_GAP_, (*k*_cat_/*K*_m_), and (*k*_cat_/*K*_m_)_E•HPi_/*K*_d_ for the L7RM (Table 1) for reasons that are not well understood.^74^

### 3.6 Side Chain Substitutions at a Hydrophobic Clamp

The movement of loop 6 toward the substrate dianion occurs alongside 90° and 180° rotations, respectively, in the planes defined by peptide bonds for G211 and G212.^67,76–78,90,91^ The peptide bond rotations create a steric clash between the backbone carbonyl oxygen of G211 and the pyrrolidine side chain of P166 that is relieved as P166 carries with it the E165 side chain 2 Å towards enzyme-bound ligand,^91,92^ and into the hydrophobic clamp created by the mobile I170 side chain from loop 7 and the stationary L230 side chain from loop 8 (Figure 6 for *Tbb*TIM, but a similar hydrophobic clamp is observed for *c*TIM and *y*TIM).^90^

**Figure 6:**
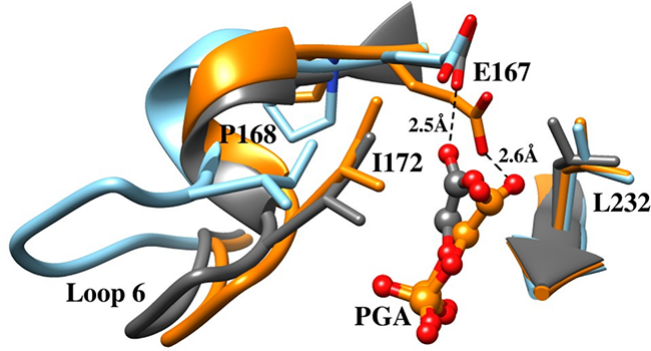
Superposition of the structures for unliganded wild-type *Tbb*TIM (cyan, PDB 5TIM), wild-type *Lm*TIM liganded to PGA (gold, PDB 1N55) and the P168A variant of *Tbb*TIM liganded to PGA (gray, PDB 2J27). Movement of P168 at the wild-type enzyme carries the E167 side chain 2 Å toward PGA and into a hydrophobic clamp composed of the mobile I172 and the stationary L232 side chains. The dotted lines show the distance separating the E167 side chain and the inhibitor carboxylate oxygen. Similar enzyme conformational changes are observed for complexes of PGA to wild-type and the P168A variant, except that the E167 side chain remains in swung-out conformation at the variant.

The roles of the conserved P166/P168, I170/I172, and L230/L232 side chains in catalysis were investigated for *Tbb*TIM by determining the effect of side chain substitutions on the kinetic parameters for wild-type enzyme-catalyzed reactions of the whole substrate GAP, and the [1-^13^C]-GA/phosphite dianion pieces (Table 2).^74,91,93–95^ The kinetic studies were carried out alongside the determination of high-resolution protein crystal structures for the P166A,^91^ I170A,^93^ L230A,^93^ and I170A/L230A^93^ variants and computational studies to model the experimental results.^23,63^

**Table 2:** Kinetic Parameters for Wild-type and Variant *Tbb*TIM-Catalyzed Reactions of GAP and the Substrate Pieces [1-^13^C]-GA and Phosphite Dianion^74,91,93–95^

enzyme *Tbb*TIM	(*k*_cat_/*K*_m_)_GAP_ (M^–1^ s^–1^) (*K*_m_)_GAP_ (M)	ΔΔ*G*_GAP_^†^ (kcal/mol)^d^	(*k*_cat_/*K*_m_)_E_ (M^–1^ s^–1^)^e^	$\frac{{\left(k_{\mathrm{cat}}/K_{m}\right)}_{E•\mathrm{HPi}}}{K_{d}}$ (M^–2^s^–1^)^f^	ΔΔ*G*_GA+HPi_^†^ (kcal/mol)^g^	Δ*G*_HPi_^†^ (kcal/mol)^h^	ΔΔ*G*_HPi_^†^ (kcal/mol)
wild-type^a^	8.4 × 10^6^		0.07	3400		–6.4	
	2.5 × 10^–4^						

P166A^b^	2.6 × 10^5^	2.1	0.0013	83	2.2	–6.5	–0.1
	9.1 × 10^–5^						

I170A^a^	8.0 × 10^4^	2.7	≤0.001	20	3.0	>−5.8	
	1.5 × 10^–4^						

L230A^a^	1.5 × 10^6^	1.0	1.2	83000	–1.9	–6.6	–0.2
	1.4 × 10^–4^						

I170A/L230A^c^	3.5 × 10^5^	1.9	≤0.001	390	1.3	nd	
	1.8 × 10^–5^						

a Reference 94.

b Reference 74.

c Reference 93,

d The effect on the activation barrier for wild-type TIM-catalyzed isomerization of GAP.

e Second-order rate constant for unactivated TIM-catalyzed reactions of [1-^13^C]-GA.

f Third-order rate constant for HP_i_ -activated TIM-catalyzed reactions of [1-^13^C]-GA (Scheme 4).

g Effect on the activation barrier for the wild-type TIM-catalyzed reaction of substrate pieces.

h Calculated using eqs 1 and 2.

#### 3.6.1 P166A Variant

The X-ray crystal structures for complexes between PGA and wild-type TIM^90,96^ or the P166A variant are nearly superimposable except that the carboxylate side chain of E165 remains in the “swung-out” conformation for the P166A variant (Figure 6).^91^ The P166A substitution (Table 2) causes a *ca* 2 kcal/mol increase in the barrier Δ*G*_GAP_^†^ for TIM-catalyzed isomerization of GAP and in the barrier Δ*G*_GA+HPi_^†^ for reaction of the substrate pieces, but there is no significant change in the dianion binding energy Δ*G*_HPi_^†^ (Scheme 4).^74,91^ The results are consistent with a *ca* 2.0 kcal/mol barrier to movement of E165 from the swung-out to the catalytically active swung-in position at the P166A variant.^91^

#### 3.6.2 I170A Variant

The only effect of the I170A substitution on the structure of the complex between TIM and PGA is to allow insertion of water at the position of the excised I170 side chain.^93^ This small change in protein structure is accompanied by 100- and >70-fold decreases respectively, in (*k*_cat_/*K*_m_)_GAP_ and (*k*_cat_/*K*_m_)_E_ for TIM-catalyzed reactions of whole and phosphodianion truncated substrates, but there is little change in the intrinsic phosphite dianion binding energy Δ*G*_HPi_^†^ (Table 2).^94^ The results show: (1) The hydrophobic I170 side chain functions to increase the reactivity of TIM for deprotonation of both the truncated substrate GA and the whole substrates DHAP/GAP. (2) The phosphite dianion-driven conformational change provides a healthy activation of wild-type and I170A variant TIM-catalyzed reactions of GA.^94^

#### 3.6.3 L230A Variant

The only effect of the L230A substitution on the structure of the complex between TIM and PGA is to allow insertion of water at the position of the excised L230 side chain.^93^ This change in structure is accompanied by a 6-fold decrease in (*k*_cat_/*K*_m_)_GAP_ and surprising *ca* 20-fold increases, respectively, in the second order rate constant (*k*_cat_/*K*_m_)_E_ and the third order rate constant (*k*_cat_/*K*_m_)_E•HPi_/*K*_d_ for unactivated and phosphite dianion activated TIM-catalyzed reactions of GA. There is no change in the intrinsic dianion binding energy Δ*G*_HPi_^†^. The increases in the kinetic parameters for unactivated and dianion-activated reactions of GA catalyzed by the L230A variant are consistent with an increase in the fraction of TIM present in the active **E**_**C**_ form (Scheme 5) due to an increase in *K*_C_ for the protein conformational change.

We consider now the role of desolvation of the enzyme active site, that accompanies substrate binding, on the basicity of the E165 side chain. The p*K*_a_ of 3.9 reported for the E165 side chain at unliganded TIM is similar to that for acetic acid, consistent with the observation that the total of six waters of solvation at the E165 carboxylate is the same as for the first solvation shell of acetate anion in water (Figure 7).^97–99^ The formation of the Michaelis complex to PGH is accompanied by extrusion of four of the six solvating waters to bulk solvent (Figure 7).^100^ The barrier to side chain desolvation, that accompanies the ligand-driven conformational change and contributes to the overall barrier *K*_C_ (Scheme 5) should be reduced by the greater solvation for the L230A variant at **E**_**C**_.^93^ We proposed that this increase in *K*_C_ is expressed as (i) An increase in the kinetic parameter (*k*_cat_/*K*_m_)_E_ that is proportional to the fraction of TIM present in the active **E**_**C**_ form (eq 3 and Scheme 5). (ii) A decrease in the dissociation constant *K*_d_ for HP_i_ activation from 19 mM to 1.2 mM,^95^ due to the reduction in dianion binding energy utilized to drive the protein conformational change.

**Figure 7:**
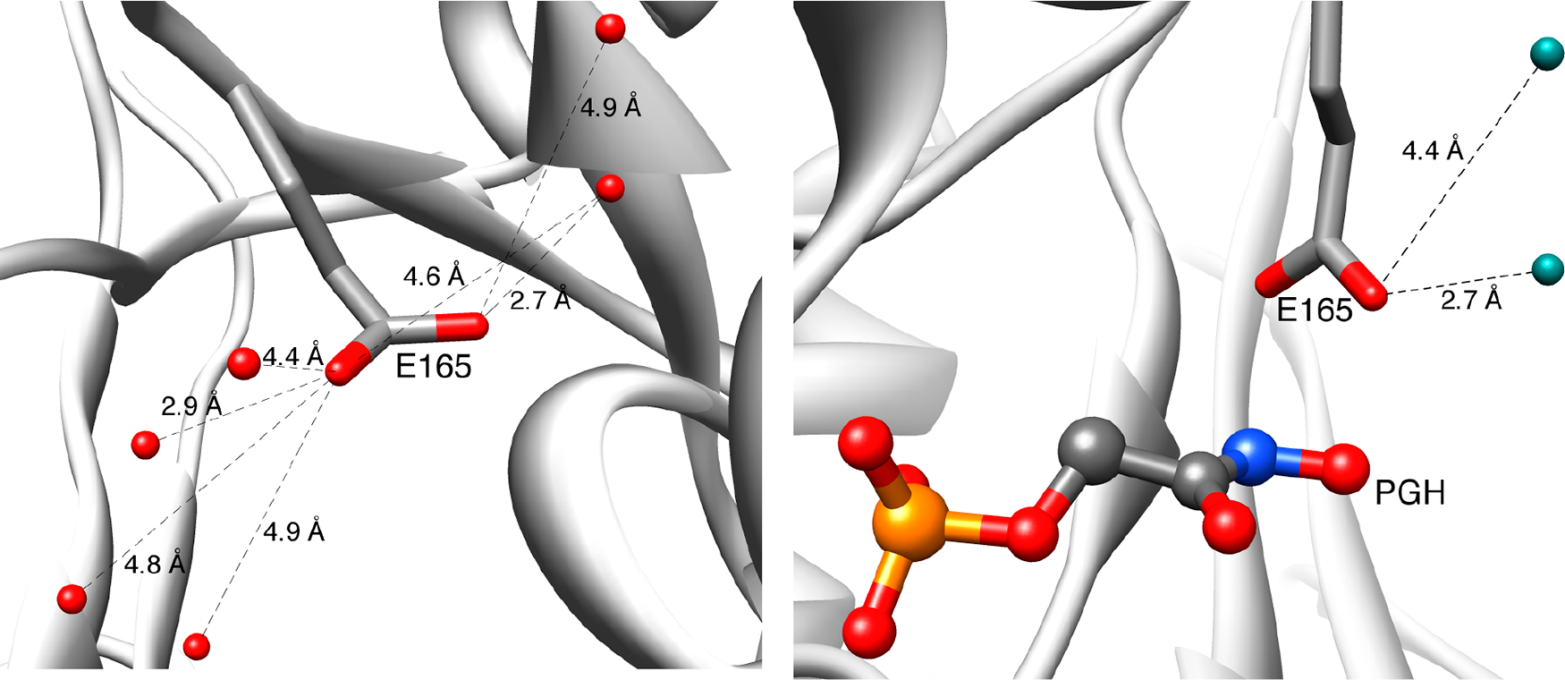
(left) The open form of *Tbb*TIM with the solvated carboxylate side chain of E165. The six solvating waters are shown as red spheres (PDB 5TIM). (right) The closed form of *Lm*TIM liganded to PGH with the desolvated carboxylate side chain (PDB 2VXN).^100^ The two solvating waters are shown as aqua spheres. Reproduced with permission from ref 29. Copyright 2014 Elsevier.

#### 3.6.4 I170A/L230A Variant

The only effect of the double I170A/L230A substitution on the structure of the complex between TIM and PGA is to allow insertion of one water molecule at the position of each excised side chain.^93^ The complex effects of these substitutions on the kinetic parameters for reactions of whole substrate and substrate pieces (Table 2) were interpreted using the model developed to rationalize the effects of single I170A and L230A substitutions.^93^ For example, the L230A substitution at the I170A variant results in a 4-fold increase in (*k*_cat_/*K*_m_)_GAP_ that is consistent with an increase in *K*_C_ for the reaction catalyzed by the double variant,^93^ while the double I170A/L230A substitutions causes 14- and 11-fold decreases, respectively, in *K*_m_ for wild-type TIM-catalyzed isomerization of GAP and DHAP. The latter effect corresponds to a 1–2 kcal/mol stabilization of the substrate Michaelis complexes, so that the I170A and L230A side chains act to destabilize the Michaelis complex at wild-type TIM by enforcing thermodynamically unfavorable desolvation of the E165 side chain (Figure 7).

#### 3.6.5 P166A/I170A Variant

The value of *k*_cat_ = 0.02 s^–1^ for P166A/I170A *Tbb*TIM-catalyzed isomerization of GAP at 25 °C is the same as *k*_cat_ = 0.02 s^–1^ at 39 °C (Scheme 6) estimated for deprotonation of GAP in a complex with the tertiary amine base quinuclidinone (p*K*_a_ = 7.5).^2,101^ The difference between *k*_cat_/*K*_m_ = 340 M^–1^ s^–1^ for P166A/I170A variant-catalyzed isomerization of GAP and *k*_cat_/*K*_d_ = 0.0018 M^–1^ s^–1^ quinuclidinone-catalyzed deprotonation of GAP is therefore due to stabilization of its Michaelis complex to the P166A/I170A variant by interactions between the protein catalyst and the substrate.

**Scheme 6:**
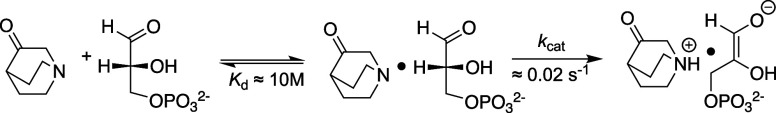
Quinuclidinone-Catalyzed Deprotonation of GAP

A comparison of the free energy profiles for wild-type and P166A/I170A variant *Tbb*TIM-catalyzed deprotonation of GAP to form the enediolate X^–^ (Figure 8) shows that this double substitution causes a 6.9 kcal/mol increase in the activation barrier to substrate deprotonation and a 1.0 kcal/mol stabilization of the Michaelis complex to GAP. In other words, the combined effect of the P166 and I170 side chains is to stabilize the transition state for TIM-catalyzed deprotonation of GAP and to destabilize the Michaelis complex of GAP at wild-type TIM. The results provide support for the conclusion that the P166 and I170 amino acid side chains facilitate the energetically uphill ligand-driven extrusion of solvating waters from the E165 side chain to bulk solvent (Figure 7) and that this causes the basicity of the E165 carboxylate side chain to increase. This conclusion is supported by the results of experiments from section 3.7 to directly determine the effect of these substitutions on the p*K*_a_ of the E165 side chain.^102,103^

**Figure 8:**
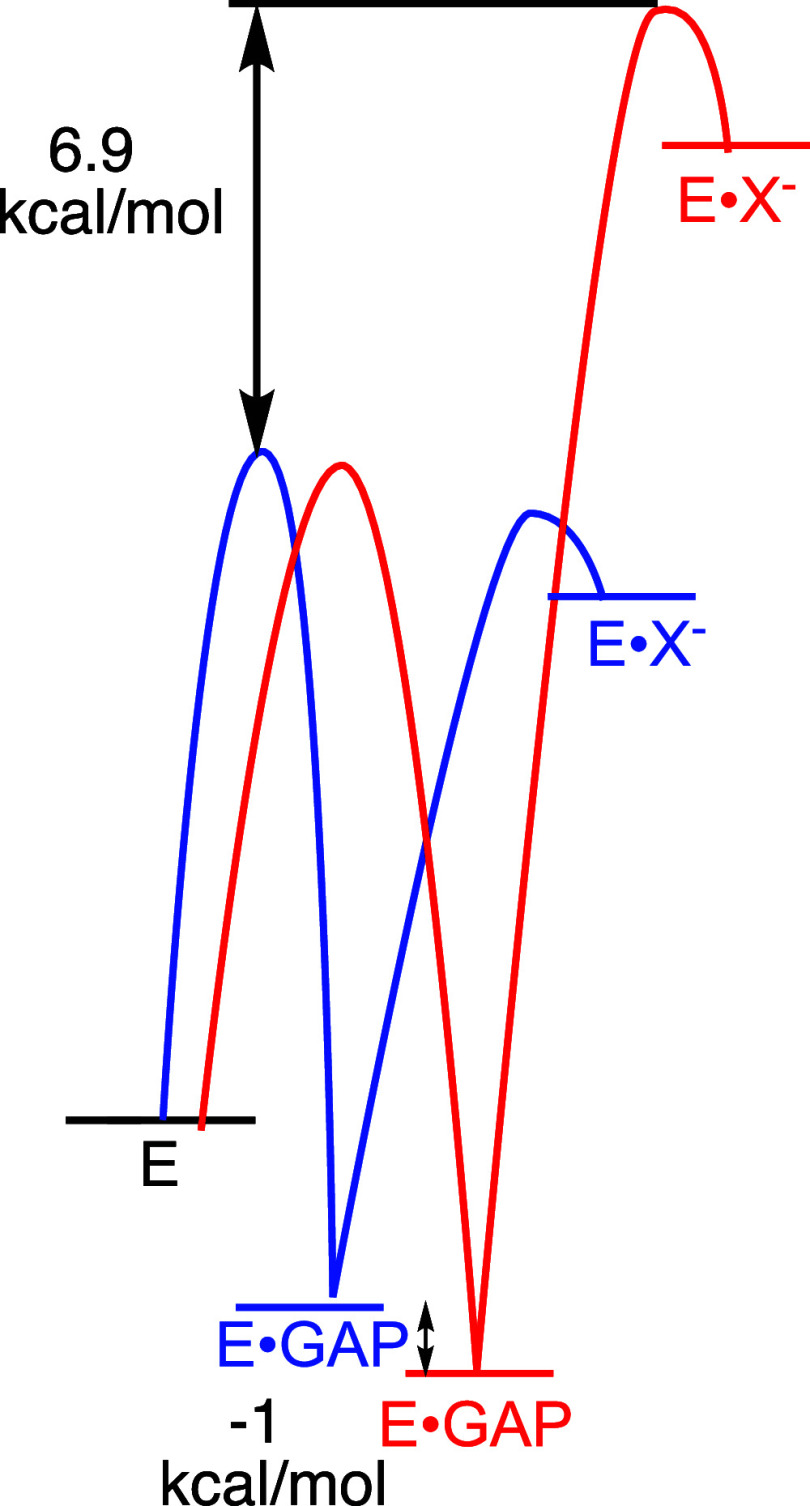
Free energy profiles for wild-type (blue profile)^35,104^ and P166A/I170A (red profile)^101^ *Tbb*TIM-catalyzed deprotonation of GAP to form the enediolate reaction intermediate X^–^. The profiles illustrate the effect of the double substitution on the activation barrier for *k*_cat_/*K*_m_ (ΔΔ*G*_GAP_^†^ = 6.9 kcal/mol) and on the stability of the Michaelis complex (Δ*G*_ES_^0^ = – 1.0 kcal/mol). Reproduced with permission from ref 101. Copyright 2023 American Chemical Society.

#### 3.6.6 Complications for a Simple Model

We have worked with a model where the P166, I170, and L230 side chains function to enhance the reactivity of the E165 base by driving extrusion of waters of solvation to bulk solvent (Figure 7). This desolvation is predicted to destabilize **E**_**C**_ relative to **E**_**O**_ while enhancing the reactivity of **E**_**C**_ toward deprotonation of bound substrate. The results of studies on variant enzymes provide support for this model and evidence that the I170 and L230 side chains act differently in promoting TIM-catalyzed substrate deprotonation.

The I170A substitution causes an increase in the number of waters solvating the E165 side chain and a decrease in enzyme reactivity toward deprotonation of whole and truncated substates (Table 2). There is no kinetic evidence that this substitution affects the stability of **E**_**C**_ relative to **E**_**O.**_ By comparison, the L230A substitution causes a similar increase in the number of waters solvating the E165 side chain, but this now results in increases in the kinetic parameters for the unactivated and dianion activated TIM-catalyzed reactions of the truncated substrate GA that are consistent with a decrease in the barrier for desolvation of inactive **E**_**O**_ to form active **E**_**C**_. The L230A substitution at wild-type TIM causes a decrease in *k*_cat_/*K*_m_ for TIM-catalyzed reactions of the whole substrate, but the effect on ΔΔ*G*_GAP_^†^ for substate isomerization is substantially smaller than observed for the I170A substitution (Table 2). The L230A substitution at the I170A variant results in a 4-fold increase in (*k*_cat_/*K*_m_)_GAP_ that is consistent with an increase in *K*_C_ for the reaction catalyzed by the double variant.^93^

The results are largely consistent with a model where the additional water of solvation at the I170A variant reduces the reactivity of the E165 side chain for substrate deprotonation but does not affect the barrier to the protein conformational change, while the additional water of solvation at the L230A variant stabilizes the active enzyme **E**_**C**_ relative to inactive **E**_**O**_ but does not affect enzyme reactivity for substrate deprotonation. The failures of the effects of side chain substitutions to conform to this model may be related to differences in side chain motion during the dianion-driven protein conformational change (Figure 6). The I170 side chain is part of the dianion gripper loop 6 and moves toward E165 during the conformational change, while the position of the L230 side chain at loop 8 relative to E165 does not change.

### 3.7 Side Chain Effects on the Basicity of the E165 Carboxylate

Results from high-level empirical valence bond (EVB) simulations show that the thermodynamic barrier for proton transfer from DHAP (p*K*_a_ = 18)^2,62^ to a carboxylate anion (p*K*_a_ ≈ 4–5) decreases from 19 kcal/mol for the reaction in water to 5.6 kcal/mol for the reaction at the TIM active site (Figure 2 and Scheme 7). This corresponds to a 10 unit effect of the dianion-driven conformational change on the difference in the p*K*_a_s of the carbon acid substrate and carboxylic acid side chain.^63^

**Scheme 7:**
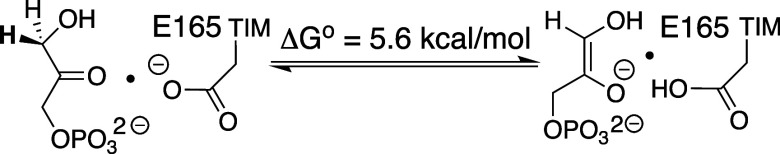
Thermodynamic Barrier for Proton Transfer from DHAP to the E165 Side Chain at TIM Determined by EVB Calulations.^63^

PGA is a tight binding analog for the TIM enediolate reaction intermediate.^105^ The values of *K*_i_ for inhibition of wild-type TIM by PGA trianion change sharply as the pH is increased from 4.9 [*K*_i_ = 7 × 10^–8^ M] to 9.9 [*K*_i_ = 7 × 10^–3^ M], because PGA trianion binds specifically to TIM with E165 in the protonated form (Scheme 8).^106–108^ The linear plots with slope of −1 for -log*K*_i_ against pH through pH 10 for inhibition of wild-type *c*TIM, yTIM and *Tbb*TIM by PGA trianion are consistent with values of p*K*_a_ ≈ 11 for deprotonation of E165 at the TIM•PGA complex (Scheme 8).^102,103^ This large perturbation in the p*K*_a_ ≈ 4–5 for a carboxylic acid in water is due to (i) The stabilizing hydrogen bond between the E165 protonated TIM and the PGA trianion. (ii) The destabilizing electrostatic interaction between the E165 carboxylate anion and the PGA trianion (Scheme 8). A similar perturbation in the p*K*_a_ for E165 at the complex to the enediolate reaction intermediate would contribute to the large effect of TIM on the reaction driving force (Figure 2 and Scheme 7).

**Scheme 8:**
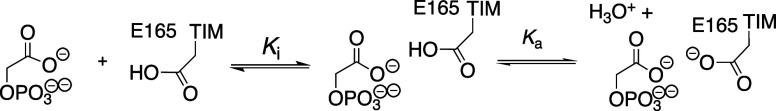
Ionization of the E165 Side Chain at the Complex to PGA Trianion

The positions on Figure 9 for upward breaks in plots of p*K*_i_ against pH for inhibition of wild-type TIM and TIM variants by PGA trianion define the following p*K*_a_s for deprotonation of E165 at the enzyme•inhibitor trianion complex (Scheme 8): P166A, p*K*_a_ = 9.3;^103^ S211A, p*K*_a_ = 8.2;^103^ I170A, p*K*_a_ = 7.7;^102^ L7RM, p*K*_a_ = 6.7;^103^ Y208F, p*K*_a_ = 6.3;^103^ and P166A/I170A, p*K*_a_ = 7.8.^101^ We concluded that these side chains function to optimize the basicity of the E165 carboxylate relative to the carbon acid substrate where (i) The P166 and I170 side chains create a hydrophobic clamp for the E165 side chain that enhances side chain basicity (Figure 6). (ii) The interactions of the Y208 and S211 side chains from loop 7 with backbone amides from loop 6 stabilize the active-site cage (Figure 3) and provide an environment that strongly favors proton transfer at the active site compared to the reaction in water.

**Figure 9:**
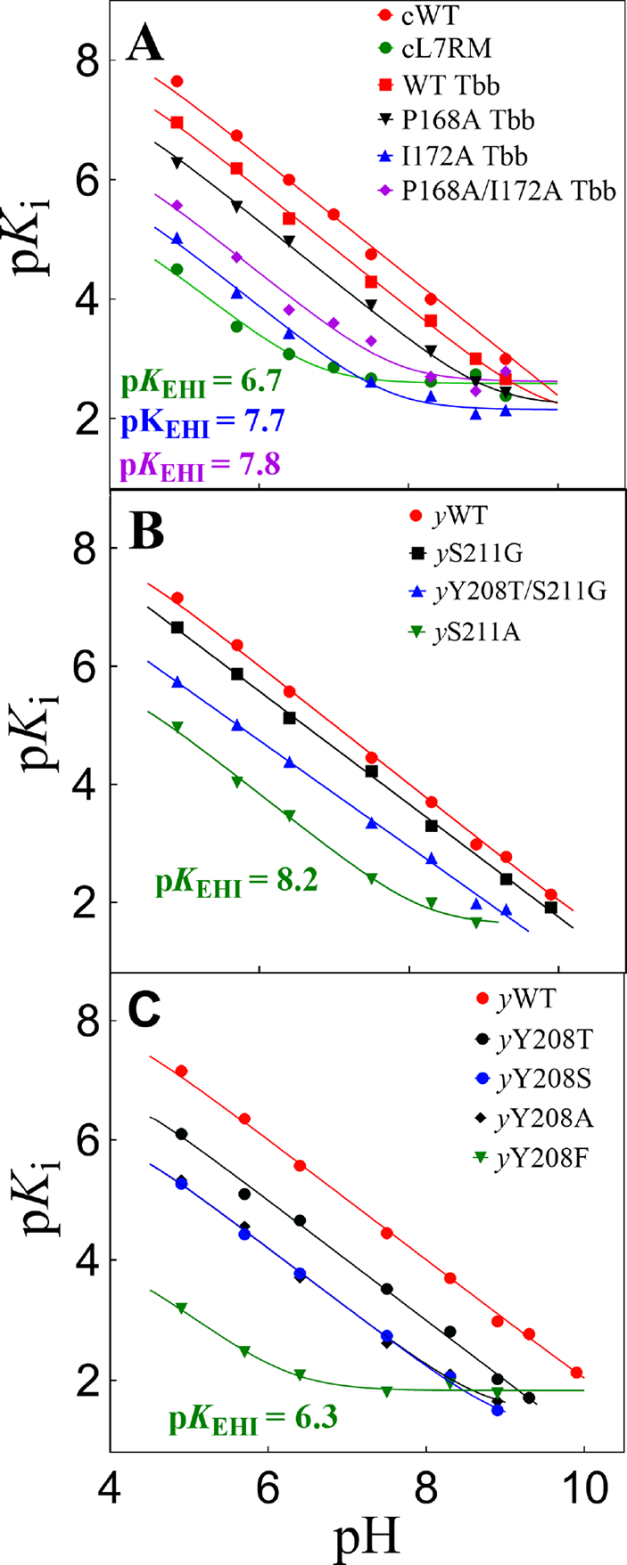
Logarithmic relationships between *K*_i_ for inhibition of wild-type and variant forms of TIM by PGA trianion and the concentration of hydrogen ion.^103^ The positions of the upward breaks in these profiles at high pH define the p*K*_a_ of the E165 side chain at the complex with the PGA trianion (Scheme 8).^106,107^

Large increases in the p*K*_a_ of the E165 side chain are expected for formation of both the complex to the enediolate analog PGA (Scheme 8) and the true enediolate reaction intermediate (Scheme 9), because each complex is stabilized by hydrogen-bonding interactions with the protonated E165 side chain and destabilized by electrostatic interactions between the neighboring anions. These effects on reactivity will not be observed for deprotonation of triosephosphates in water where hydrogen bonding and electrostatic effects are attenuated, compared to the nonpolar enzyme active site, by the polar hydrogen bonding solvent.^109^

**Scheme 9:**
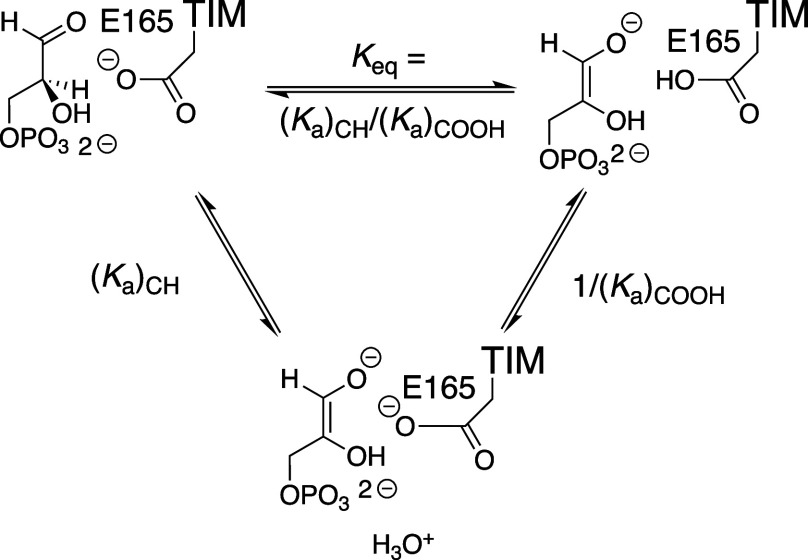
Equilibrium Constant *K*_eq_ for Proton Transfer from Enzyme-Bound GAP to the Carboxylate Side Chain of TIM Expressed as the Ratio [(*K*_a_)_CH_/(*K*_a_)_COOH_] of Acidity Constants for Proton Transfer from Enzyme to Water

The driving force for proton transfer from substrate to E165 at TIM compared to proton transfer in water is determined by the difference in the p*K*_a_s of the carboxylic acid [(*K*_a_)_COOH_] and carbon acid substrate [(*K*_a_)_CH_] in water or at the TIM active site-effective catalysis is favored by a minimal difference in these p*K*_a_s (Figure 2).^110^ A computational study is needed to model the effect of substitutions of TIM side chains on the p*K*_a_ of the E165 side chain at the complex to substrate and at the complex to the enediolate reaction intermediate.

### 3.8 Other Side Chain Substitutions

The TIM active-site side chains are segregated into side chains at TIM barrel loops 6 and 7 that undergo dianion-driven changes in their position at the enzyme active site, and side chains at loop 1 (N10 and K12), loop 3 (Q64) loop 3′ (T75′ and G76′) and loop 4 (H95, S96, E97, and R98) whose positions remains fixed during movement of loops 6 and 7.^111–113^ This is illustrated by the superposition of loop 1, 3, 3′, and 4 side chains at the active sites for open unliganded *y*TIM and the closed Michaelis complex to DHAP (Figure 10). The side chains from mobile loops 6 and 7 (not shown) converge around the substrate dianion during the protein conformational change, while the catalytic side chains from loops 1, 3, 3′, and 4 sit in a tight network of hydrogen bonds. This hydrogen bond network plays the important role of holding the catalytic side chains from loops 1, 3, 3′, and 4 at fixed positions during the closure of loops 6 and 7 over the substrate dianion.

**Figure 10:**
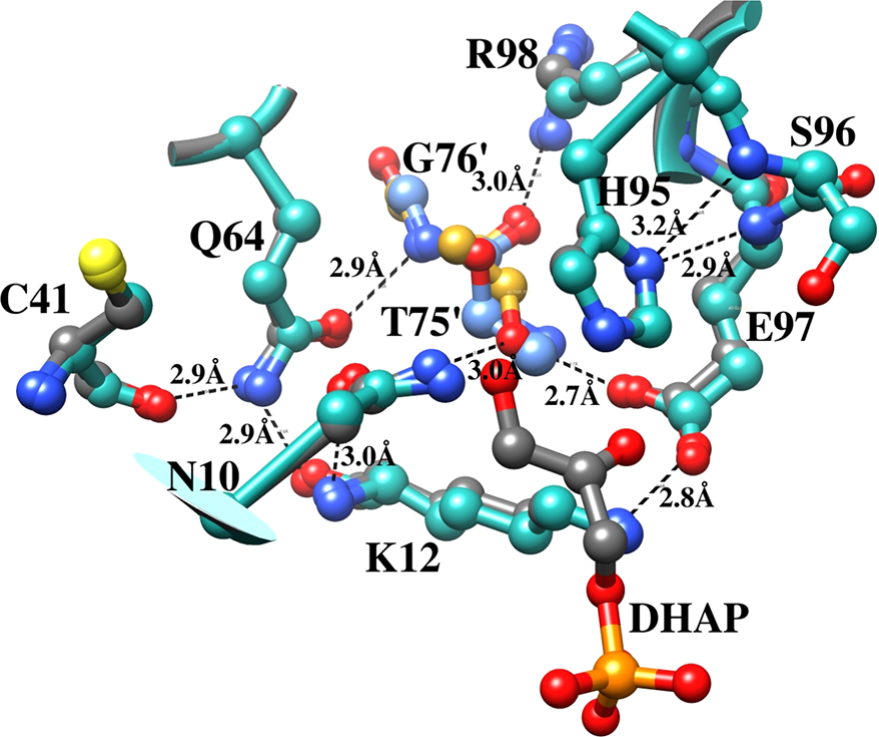
Superposition of the structures for unliganded *y*TIM (aqua, PDF 1YPI) and for the Michaelis complex to DHAP (gray, PDB 1NEY). The T75′ and G76′ residues from the second subunit for the TIM homodimer are highlighted as blue and gold for the free enzyme and Michaelis complex.

We have emphasized the similarity in the structure of the active sites for TIM from different organisms, because this supports the generalization of results from studies on substitution of side chains at one orthologue to all TIMs. However, different effects are sometimes observed for the same substitution of highly conserved amino acid side chains in different TIM orthologues. We propose that these orthologue-specific effects reflect subtle differences in the packing of active-site side chains that arise from small and difficult to detect changes in the highly conserved network of hydrogen bonded residues illustrated in Figure 10. By contrast, we propose that the effects of substitutions at mobile loops 6 and 7 will be more broadly generalizable to all TIM orthologues.

For example, the E97D and E97Q substitutions at TIM from *Plasmodium falciparum* (*Pf*TIM) result in 100- and 4000-fold decreases, respectively, in *k*_cat_/*K*_m_ for enzyme-catalyzed isomerization; and, a large displacement of the K12 side chain due to a gauche–trans rotation about the C_γ_/C_δ_ side chain bond.^58^ By comparison the E97D variant at *c*TIM has no detectable effect on enzyme kinetic parameters because the packing of K12 allows the side chain position to remain fixed at the E97D variant.^45^ The E97D and E97A substitutions at *Tbb*TIM, *y*TIM, and TIM from *Homo sapiens* (*hs*TIM) likewise cause only small changes in the wild-type enzyme kinetic parameters, while the E97Q substitution causes 4-fold and 20-fold reductions in *k*_cat_/*K*_m_ for isomerization reactions catalyzed by *y*TIM and *hs*TIM.^57^

The K12 side chain amide bond has a strained conformation and a bond dihedral angle that lies in the fourth quadrant of the Ramachandran plot (Φ = 59, Ψ = −148) that is disallowed for every amino acid except glycine.^114^ The strained conformation is enforced by packing interactions of the K12 side chain with neighboring N10, M14, Q64, and E97 side chains and with the substrate dianion.^41^ It was proposed that substitutions that relieve destabilizing packing interactions at the K12 side chain might promote structural reorganization to an allowed dihedral bond angle and cause a significant falloff in enzyme activity when there is an increase in the separation of the K12 side chain cation and substrate dianion. The M14A, Q64A, E97A and N10A variants of *hs*TIM were prepared to test this hypothesis. Robust activity was observed for the first three variants, but no activity was detected for N10A. Molecular dynamics simulations for the N10A variant provide evidence for movement of the K12 dihedral angle to an allowed value, while the unfavorable dihedral angle is retained for simulations at the other variants.^115^

### 3.9 Catalysis by a Monomeric Variant of TIM

The dimer interface at TIM monomers buries ca. 1600 Å of protein surface,^98^ and is composed of residues from TIM barrel loops 1–4,^111^ that includes active-site side chains from loops 1 and 4. In the case of *Tbb*TIM, loop 3 residues contribute 203 of 256 non-hydrogen atoms that lie within 4 Å of the subunit interface.^116^ The monomer–monomer interactions strongly stabilize TIM to temperature- and urea-induced unfolding.^117,118^ Reducing stabilizing intersubunit interactions of loop 3 side chains favors formation of a stable monomeric form of TIM (*mono*TIM).^75^ The *mono*TIM designed by Wierenga and co-workers eliminated a hydrophobic patch at the loop 3 dimer interface by deletion of seven central loop residues, optimized the thermal stability of the designed protein by a P81/A74 substitution that allowed for an additional α-helical turn, and reduced loop 3 hydrophobicity through I68G and S79/A72 substitutions (Figure 4).^75^

Engineered *mono*TIM is more stable at room temperature than the wild-type monomer, and has a 20-fold greater *K*_m_ and 400-fold smaller *k*_cat_ compared with the wild-type dimer.^75,119^ There is no detectable catalysis by *mono*TIM of isomerization of [1-^13^C]-GA in D_2_O in the presence or absence of added phosphite dianion.^119^ The binding of sulfate dianion, or the intermediate analogs PGH or PGA to *mono*TIM drives conformational changes similar to those observed for wild-type TIM.^120,121^ These conformational changes may result in enzyme activation by phosphite dianion that is too low to detect by our methods.

The X-ray crystal structures of unliganded *mono*TIM and for complexes to PGA and PGH inhibitors show TIM barrel protein folds.^120,121^ Loops 1 and 4 are held close to the dimer interface at wild-type TIM, but their large conformational flexibility at *mono*TIM gives rise to shifts in the position of the K12 and H95 active-site side chains.^120^ The falloff in the kinetic parameters for K12A and H95A variants of *mono*TIM show that these side chains continue to carry out their wild-type function. The reduction in activity of *mono*TIM is therefore partly or entirely due to a reduction in the fraction of *mono*TIM present in the active conformation.^122^

### 3.10 Side Chain Substitutions that Alter Dimer Stability

The active-site side chains at one TIM monomer interact across the monomer interface with side chains at the second subunit. For example, the T75′ side chain of *y*TIM forms hydrogen bonds to the E97 and N10 active-site side chains at the second subunit, while the Q64 side chain is hydrogen bonded to K12 and G76′ backbone amides (Figure 10). T75′ is highly conserved at TIMs from different organisms, but position 64 can accept either E or Q. The Q64N or Q64E substitutions at TIM from *Plasmodium falciparum* each favor dimer to monomer dissociation. However, T75′V or T75′N substitutions do not affect dimer stability, but cause large reductions in enzyme activity.^123^

The conserved N10 amide side chain interacts with the substrate O-1 (Figure 10). The determination of the contribution of this side chain to the catalytic rate acceleration was complicated by the observation that the N10A variant of *Tbb*TIM undergoes dissociation from the dimer to monomer during enzyme assays at 25 °C.^124^ The N10A variant of *Lm*TIM is more stable to dissociation than the N10A variant of *Tbb*TIM and the E65Q substitution at wild-type *Lm*TIM further stabilizes this enzyme to dissociation without effect on the enzyme catalytic activity.^125,126^ The N10A/E65Q double variant of *Lm*TIM is stable to dissociation and the N10A substitution at E65Q *Lm*TIM results in the (13–14)-fold decrease in *k*_cat_/*K*_m_ for the variant, a similar decrease in *k*_cat_ for the catalysis of isomerization of DHAP, but only a 2-fold decrease in *k*_cat_ for GAP.^124^ There is evidence that the rate-determining step for *k*_cat_/*K*_m_ for the N10A/E65Q variant-catalyzed isomerization of GAP is substrate deprotonation.^124^ The (13–14)-fold effects on *k*_cat_/*K*_m_ are consistent with a 1.5 kcal/mol stabilizing interaction between the N10 amide side chain and the substrate O-1 at the rate-determining transition state for substrate isomerization.

A full discussion of the many reports of amino acid substitutions that destabilize the dimeric form of TIM orthologues is outside the scope of this review.^117,127–132^ The results of these studies provide interesting and perhaps unprecedented insight into the inter- and intrasubunit side chain interactions that maintain the active dimeric enzyme. These results are worth building on in future work.

### 3.11 Summary

The phosphodianion of substrates DHAP or GAP provides a 12 kcal/mol stabilization of the transition state for TIM-catalyzed isomerization while phosphite dianion provides a 6 kcal/mol stabilization of the transition state for TIM-catalyzed deprotonation of GA. The dianion binding energy is utilized to generate an enzyme active site that provides strong stabilization of the enediolate phosphate reaction intermediate relative to the carbon acid substrate.

No phosphite dianion activation is observed for K12G variant-catalyzed or for the L6DM-catalyzed reactions of GA. The first observation shows that interactions between the K12 side chain cation and the activator dianion account for a large fraction of the dianion binding energy.^55^ Since loop 6 shows only a single interaction with the substrate dianion, the second observation shows that closure of this loop over substrate is required to optimize dianion interactions with the K12 side chain and other protein backbone amides. In other words the closure of loop-6 is driven by development of these strong stabilizing protein dianion interactions at the closed enzyme.^70,71^ The loop 6 replacement substitution strongly destabilizes the closed form of TIM. This gives rise to the very large utilization of dianion binding energy to drive the activating protein conformational change, so that little dianion binding energy is available to stabilize the substrate Michaelis complex.^71^

The TIM-barrel fold combines rigid secondary structural elements and protein loops that are either flexible or rigid depending upon their role in catalysis.^111^ The side chains at loops 6, 7, and 8 interact mainly with the substrate dianion. Loop 6 and 7 side chains participate in a protein conformational change that promotes substrate deprotonation by causing a large increase in basicity of the E165 side chain.^103^ The side chains from loops 1, 3, and 4 interact mainly with the carbon acid fragment of the substrate and remain locked, during the dianion-driven protein conformational change, at positions that provide for optimal stabilization of the transition state for substrate deprotonation.

The P166 and I170 side chains from loop 6 and the L230 side chain from loop 8 cooperate to trap the basic E165 side chain at a hydrophobic clamp. Substitutions of P166, I170, and L230 side chain have little effect on the protein–dianion interactions that determine the total intrinsic dianion binding energy Δ*G*_HPi_^†^ (Table 2 and Scheme 4). Substitutions of the P166 and I170 side chains weaken the hydrophobic clamp and result in decreases in enzyme activity for catalysis of reactions of the whole substrate and the dianion truncated substrate (Table 2), and a decrease in the p*K*_a_ of the E165 side chain (Figure 9). Substitution of the L230 side chain results in a 20-fold increase in the kinetic parameters for TIM-catalyzed reactions of the dianion truncated substrate that are consistent with *ca* 2 kcal/mol stabilization of the active, closed, enzyme **E**_**C**_ relative to **E**_**O**_.

There are interesting and sometimes difficult to interpret differences in the effects of substitution of Y208 and S211 side chain on the kinetic parameters for TIM-catalyzed reactions of whole and truncated substrates. The Y208T/S/A substitutions at loop 7 perturb the substrate cage and weaken the protein dianion interactions that determine the total intrinsic dianion binding energy Δ*G*_HPi_^†^ (Table 1 and Scheme 4). These substitutions cause little change in enzyme reactivity for catalysis of the reaction of the dianion truncated substrate. The Y208F substitution results in an additional decrease in the kinetic parameters *k*_cat_/*K*_m_ and (*k*_cat_/*K*_m_)_E_, respectively, for reactions of for GAP and GA that are consistent with a destabilization of active enzyme **E**_**C**_ relative to inactive **E**_**O**_ that gives rise to a decrease in *K*_C_ (Table 1 and Scheme 1). The S211A/G substitutions cause little change in Δ*G*_HPi_^†^, but the effect of the S211A substitution on (*k*_cat_/*K*_m_)_E_ for reactions of GA is likewise consistent with a decrease in *K*_C_ for the protein conformational change. Substitutions of either the Y208 or S211 side chains result in a decrease in the p*K*_a_ of the E165 side chain (Figure 9).

The side chains from loops 1, 3, and 4 are involved in an extensive network of hydrogen bonds which function to hold the N10, K12, and H95 side chains at their active positions during the protein conformational change (Figure 10). The side chains from these three loops are tightly packed at the enzyme active site, so that side chain substitutions which affect this packing sometimes result in substantial but difficult to rationalize changes in enzyme activity.^7,45,46^

## 4 Glycerol 3-Phosphate Dehydrogenase (GPDH)

### 4.1 Introduction

GPDH catalyzes the reversible reduction of DHAP by NADH to form glycerol 3-phosphate (G3P) and NAD^+^ (Scheme 10A) and phosphite dianion activated reduction of GA by NADH (Scheme 10B). The enzymatic reaction connects the metabolism of glucose to the biosynthesis of lipids by diverting DHAP formed in glycolysis toward pathways for the biosynthesis of phosphoglycerides.^133^

**Scheme 10:**
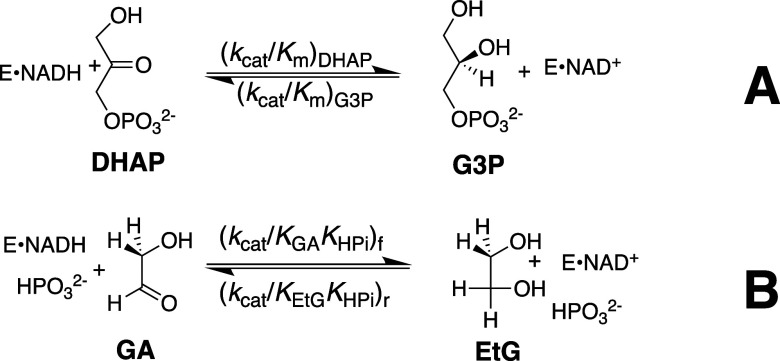
Reversible Hydride Transfer Reactions of Whole Substrate (A) and Substrate Pieces (B) Catalyzed by GPDH

Experiments to examine inhibition, by a mixture of GA and phosphite dianion, of TIM-catalyzed isomerization of GAP to form DHAP failed, serendipitously. The major reaction product when GPDH was used as a coupling enzyme to catalyze reduction of isomerization reaction product DHAP by NADH was NAD^+^ that forms instead by phosphite dianion-activated GPDH-catalyzed reduction of the GA inhibitor.^12^ These results foreshadowed the ease of the detection of enzyme-catalyzed reactions of glucose 6-phosphate and the substrate pieces xylose + phosphite dianion.^16^

It is no coincidence that TIM, OMPDC and GPDH have similar kinetic parameters for catalysis of the reaction of whole substrates (*k*_cat_/*K*_m_ = 10^6^–10^8^ M^–1^ s^–1^), of phosphodianion truncated substrates (*k*_cat_/*K*_m_ = 0.03–0.06 M^–1^ s^–1^), and for phosphite dianion activated reactions of truncated substrates (*k*_cat_/*K*_d_ *K*_HPi_ = 10^3^–10^4^ M^–2^ s^–1^).^13^ The values of *k*_cat_/*K*_m_ for whole substrates are close to the limit for diffusion-controlled reactions. The ratio of rate constants for the respective enzyme-catalyzed reactions of whole and phosphodianion truncated substrates (10^8^) and for phosphite dianion activated and unactivated reactions of truncated substrates (10^4^ M–10^5^ M) give similar phosphodianion binding energies of 12 kcal/mol and phosphite dianion binding energies Δ*G*_HPi_^†^ of (6–8)-kcal/mol (eq 2 and Scheme 4). These results from studies on highly evolved metabolic enzymes are consistent with the notion that 12 kcal/mol is the limit for stabilization of enzymatic transition states from protein-dianion interactions, and that (6–8) kcal/mol is the limit for the binding energy captured in phosphite dianion-driven protein conformational changes. Each of these enzymes accept the following dianion activators at phosphodianion binding sites; HPO_3_^2–^, FPO_3_^2–^, S_2_O_3_^2–^, SO_4_^2–^, and HOPO_3_^2–^.^13,134^ The falloff in the kinetic parameters for activation by these structurally homologous dianions is readily accounted for by the changes in dianion structure.^13^

### 4.2 The Activating Protein Conformational Change

The substrate-driven protein conformational change for GPDH and the catalytic side chains that lie close to the enzyme-bound substrates were characterized by analysis of X-ray crystal structures for the unliganded enzyme from human liver (*hl*GPDH, PDB 6E8Y), the enzyme Michaelis complex to NAD^+^ (PDB 6E8Z), and the nonproductive E•NAD^+^•DHAP complex (PDB 6E90).^87^ The apoenzyme and the complex to NAD^+^ (Figure 11A) are both in the domain-open form. The binding of DHAP to give the nonproductive ternary complex (Figure 11B) drives a domain clamping motion and the folding of a flexible loop [magenta, 292-LNGQKL-297] over the mouth of the enzyme active site.^87^ A second set of structures were determined for *hl*GPDH crystallized in the presence of sulfate dianion,^135^ which is an activator for *hl*GPDH-catalyzed reduction of GA by NADH.^13^ All three structures from this set are in the domain-closed form shown in Figure 11B. However, the E•SO_4_^2–^ complex (PDB 1X0V) has the flexible loop in the open conformation for Figure 11A, the E•NAD^+^• SO_4_^2–^ complex (PDB 1X0X) has the loop in the closed form for Figure 11B, and the binding of DHAP and release of SO_4_^2–^ causes no further changes in protein structure (PDB 1WPQ).^135^ A comparison of the two sets of crystal structures shows: (i) The apoenzyme exists in the open form. (ii) The E•SO_4_^2–^ complex exists in the domain-closed form but with an open flexible loop. (iii) The binding of NAD^+^ to the E•SO_4_^2–^ complex drives closure of the [292-LNGQKL-297] loop over the mouth of the enzyme active site.

**Figure 11:**
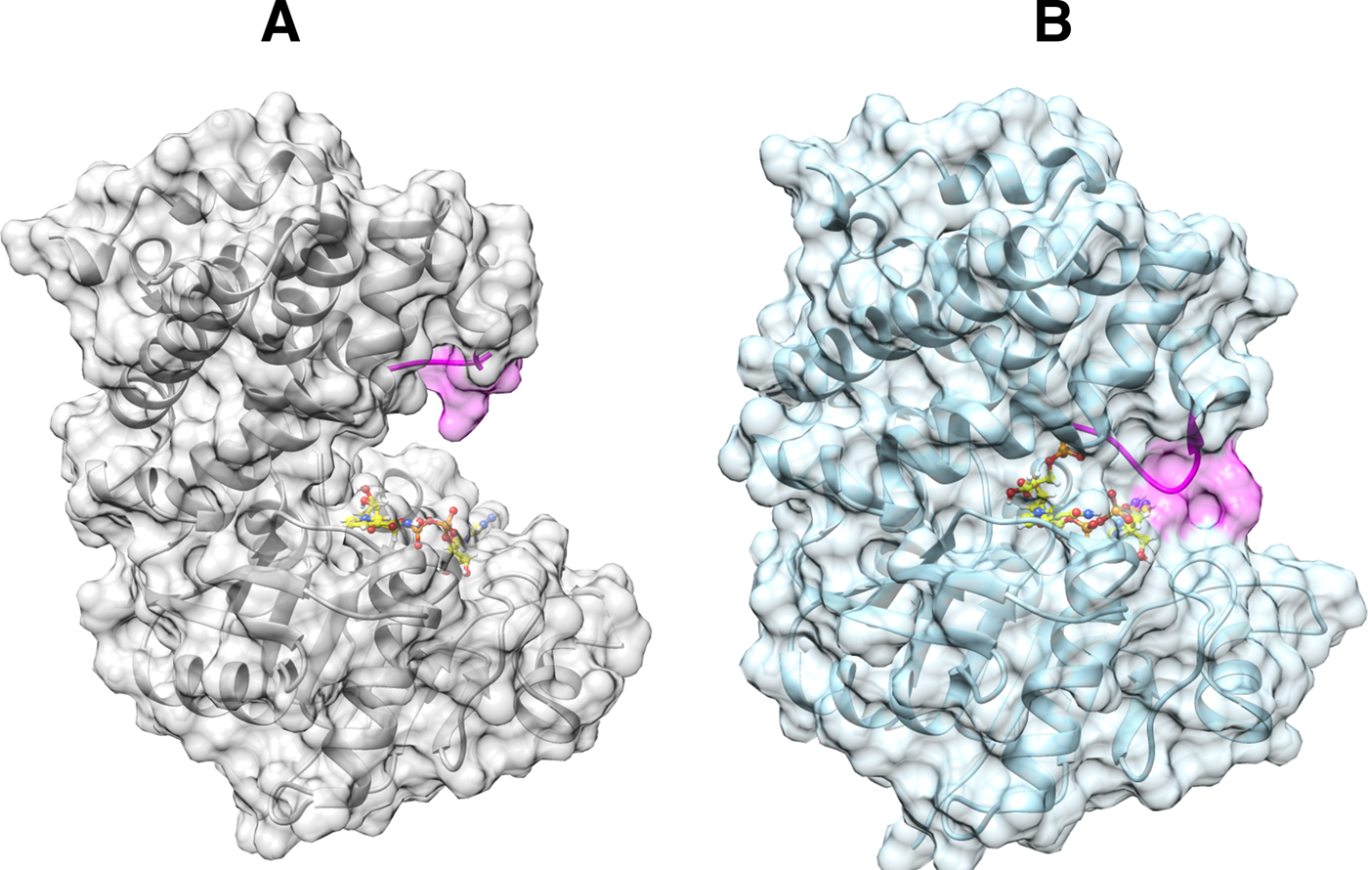
Representations of the X-ray crystal structures for *hl*GPDH with the [292-LNGQKL-297] loop shaded magenta. A: The open protein conformation **E**_**O**_ for the binary E•NAD^+^ complex (PDB 6E8Z). B: The closed conformation **E**_**C**_ for the nonproductive ternary E•NAD^+^•DHAP complex (PDB 6E90). Reproduced with permission from ref 20. Copyright 2024 American Chemical Society.

Substrate binding to *hl*GPDH is ordered, with NAD^+^ binding before G3P.^136–138^ There is no evidence that binding of NAD^+^ to the unliganded enzyme causes a large protein conformational change that creates the DHAP binding site.^87^ Rather, X-ray crystallographic results demonstrate that binding of DHAP to the E•NAD^+^ complex drives closure of the magenta [292-LNGQKL-297] loop over the mouth of the enzyme active site and that this blocks the binding and release of the cofactor.^87^ The binding of DHAP to the unliganded enzyme has been detected as substrate inhibition of enzyme-catalyzed hydride transfer.^136,139^ DHAP should act like sulfate dianion and drive domain closure upon binding to the unliganded enzyme.^135^ This would block cofactor binding and enforce the ordered sequence for substrate binding.

Binding of DHAP to the binary E•NAD^+^ complex drives an activating protein conformational change (Figure 11). Protein interactions with the cofactor might also specifically stabilize **E**_**C**_; however, no large conformational change is observed upon binding of NAD^+^ to apoenzyme to form the binary E•NAD^+^ complex (Figure 11A). The binding of NAD^+^ to the binary enzyme–sulfate complex causes the [292-LNGQKL-297] loop to move by about 5 Å to form hydrogen bonds with the enzyme-bound cofactor.^135^

*hl*GPDH shows a low activity for catalysis of hydride transfer from G3P to the truncated cofactor nicotinamide riboside (NR), and enzyme-catalyzed hydride transfer to NR is activated by AMP and ADP fragments from the whole cofactor (Scheme 11).^20^ The observation that a large fraction of the activity of the whole NAD^+^ cofactor is recovered in the reaction of the NR + AMP or NR + ADP pieces provides evidence that closure of the [292-LNGQKL-297] loop is driven by interactions with the AMP or ADP cofactor fragments that activate *hl*GPDH for catalysis of hydride transfer (see section 4.9.).^20^

**Scheme 11:**
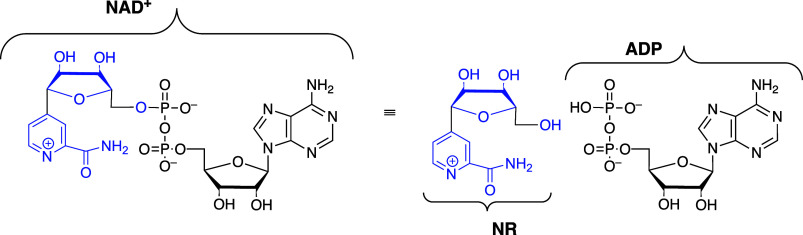
Comparison of the Whole NAD^+^ Cofactor and the NR + ADP Cofactor Pieces

### 4.3 Active-Site Side Chains

The following active-site amino acid side chains for GPDH from different organisms are highly conserved; Q295, R269, N270, N205, T264, K204, D260, and K120.^140^ A superposition of the structures for the open binary E•NAD^+^ and the closed ternary E•NAD^+^•DHAP complexes for *hl*GPDH (Figure 12) demonstrates that the dianion-driven conformational change is accompanied by formation of a network of hydrogen bonds for the conserved amino acid side chains that stabilize the loop closed enzyme.^87^ We have divided these side chains into two groups.

**Figure 12:**
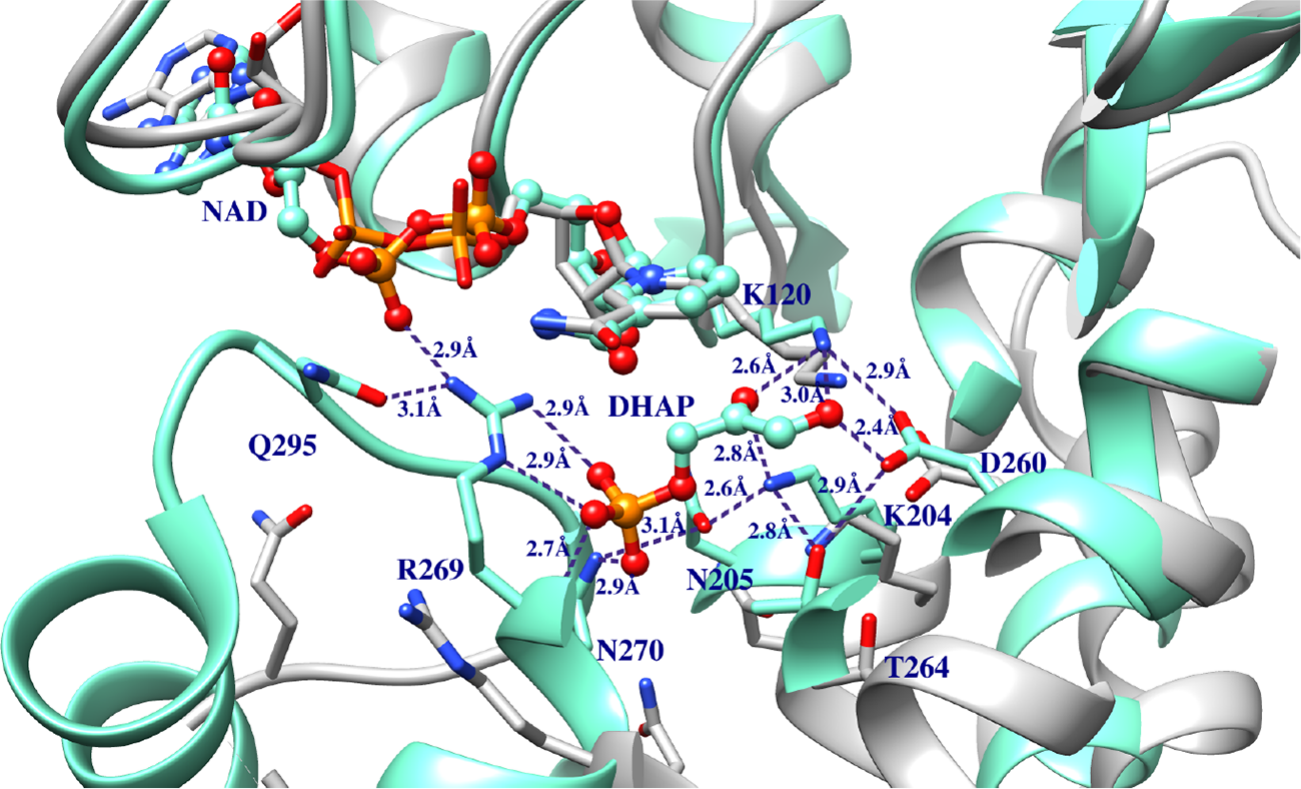
An overlay of the structures for the binary E•NAD^+^ complex to *hl*GPDH (gray, 6E8Z) and the ternary nonproductive E•NAD^+^•DHAP complex (green, PDB 6E90) that lists the distances that separate the active-site side chains from one another and from enzyme-bound DHAP. Reproduced with permission from ref 139. Copyright 2024 American Chemical Society.

(1) *R269*, *N270*, and *Q295*. These three side chains are scattered across the enzyme active site at the open binary E•NAD^+^ complex and move during the phosphodianion-driven protein conformational change to form a side chain cluster that is stabilized by (a) An ion pair and a hydrogen bond, respectively, between the substrate phosphodianion and the R269 and N270 side chains. (b) The extended network of hydrogen bonds that include the conserved Q295, R269, N270, N205, T264, K204, D260, and K120 side chains. (c) The hydrogen bond between the R269 and Q295 side chains that holds the R269 side chain cation close to the substrate dianion.^86^ (d) The ion pair between the R269 side chain cation and the α-adenosyl phosphate of NAD^+^ that connects the DHAP and cofactor binding sites.

(2) *K120*, *K204*, and *D260.* These side chains are involved in catalysis at the reacting carbonyl group of DHAP. The K120 and K204 side chains interact directly with the carbonyl oxygen and the D260 side chain forms an ion pair to the K120 side chain cation.

### 4.4 Studies on Variant Enzymes

#### 4.4.1 R269A Variant

The R269A substitution results in a 41,000-fold decrease in *k*_cat_ and 4.5 × 10^6^-fold decrease in *k*_cat_/*K*_m_ for *hl*GPDH-catalyzed hydride transfer from NADH to DHAP. These correspond, respectively, to 6.3 and 9.1 kcal/mol increases in the reaction activation barriers.^141^ There is no detectable unactivated or phosphite dianion activated R269A variant-catalyzed hydride transfer from NADH to the truncated substrate GA, so that R269 is required to observe the *hl*-GPDH-catalyzed reactions of the substrate pieces.^141^ Combining the upper limit for the rate constant for variant-catalyzed reaction of GA + HP_i_, *k*_cat_/*K*_d_ *K*_HPi_ ≤ 0.1 M^–2^ s^–1^ (Scheme 10) with *k*_cat_/*K*_d_ *K*_HPi_ = 16000 M^–2^ s^–1^ for wild-type *hl*GPDH gives *a* > (1.6 × 10^5^)-fold effect for the side chain substitution. This corresponds to *a* > 7.1 kcal/mol side chain stabilization of the transition state for reaction of the substrate pieces. The X-ray crystal structure for the binary complex between NAD^+^ and the R269A variant has the open structure observed for wild-type *hl*GPDH. It was not possible to obtain crystals for the R269A variant ternary complex, presumably because DHAP binds only weakly to this variant.^142^

The 110-fold increase in *K*_m_ for R269A variant compared to wild-type *hl*GPDH-catalyzed reduction of DHAP is due to the loss of ground-state interactions between the R269 side chain and the substrate dianion. The remaining 6.3 kcal/mol effect on the reaction activation barrier represents interactions that are specifically expressed at the hydride-transfer transition state. By comparison, interactions between the K12G side chain at TIM and the substrate dianion stabilize the Michaelis complex to DHAP by 2–3 kcal/mol and an additional 5–6 kcal/mol of side chain binding energy is expressed specifically at the isomerization reaction transition state.^55^ DHAP is a common substrate for these enzymatic reactions and the common K12 or R269 side chain cation interactions include: (a) Ground state side chain interactions with the substrate phosphodianion that stabilize the Michaelis complex. (b) Stabilizing side chain interactions that develop on moving from the **E**_**C**_ **•DHAP** Michaelis complex to reaction transition states where there are increases in the formal negative charge at the carbonyl oxygen.

The R269A substitution induces changes in the X-ray crystal structure for the open apoenzyme.^142^ However, the efficient rescue of the R269A variant by guanidine cation provides support for the conclusion that the only important difference in the structures for the closed ternary complexes for wild-type and R269A variant is the missing side chain cation at the latter, whose function is efficiently mimicked by the side chain fragment guanidine.^141^

One interpretation of the 9.1 kcal/mol effect of the R269A substitution on the activation barrier to hydride transfer is that this reflects only the transition state stabilizing R269 side chain interactions that are eliminated by the substitution. However, the R269A substitution perturbs the extended network of catalytic side chains shown in Figure 12, and this may weaken transition state stabilization by other network side chains. For example, the K120 side chain is positioned to interact with the carbonyl oxygen of DHAP and stabilize negative charge that develops at this oxygen at the hydride transfer transition state (section 4.4.4).^87^ The variant cycle from Figure 13 shows that the R269A substitution at wild-type *hl*GPDH destabilizes the hydride-transfer transition state by 9.1 kcal/mol but that the effect of the same substitution at the K120A variant is only 6.7 kcal/mol (Figure 13). In other words, the observed 9.1 kcal/mol effect of the first R269A substitution is equal to the sum of the 6.7 kcal/mol R269 side chain interaction determined for the R269A substitution at the K120A variant and the 2.4 kcal/mol decrease in transition state stabilization by the K120 side chain at the R269A variant

**Figure 13:**
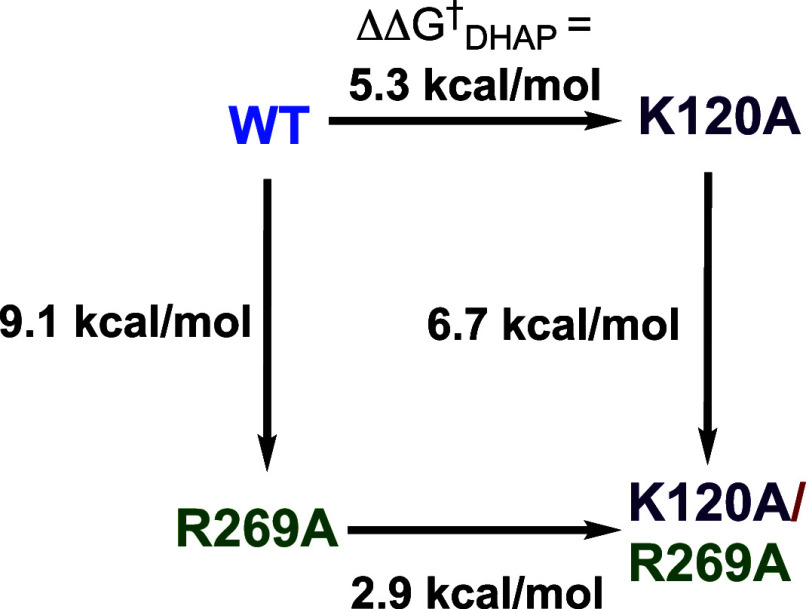
Variant cycle that illustrates the effect ΔΔ*G*_DHAP_^†^ of consecutive substitutions of amino acid side chains on the activation barrier Δ*G*_DHAP_^†^ for *hl*GPDH-catalyzed reduction of DHAP by NADH. The values of ΔΔ*G*_DHAP_^†^ were calculated from the ratio of the values of *k*_cat_/*K*_m_ for the parent and variant enzyme-catalyzed reactions.

Roughly 70% (6.7 kcal/mol) of the 9.1 kcal/mol effect of the R269A substitution on Δ*G* ^†^ for *hl*GPDH-catalyzed reduction of DHAP is recovered upon addition of 1.0 M guanidine cation (Gua^+^), which substitutes for the excised side chain cation at the enzyme active site (Figure 14C).^141,143^ Gua^+^ also substitutes for the R269 side chain cation in phosphite dianion activated reduction of GA catalyzed by the R269A variant (Figure 14D).^143^ The ease of self-assembly of dianion and cation activators is due in part to the Q295 side chain (Section 4.4.2.) that (i) holds R269 at wild-type *hl*GPDH close to the substrate dianion (Figure 14A) or to phosphite dianion (Figure 14B). (ii) Holds the Gua^+^ activator at the R269A variant close to the substrate dianion (Figure 14C) or to phosphite dianion (Figure 14D).^47,48^

**Figure 14:**
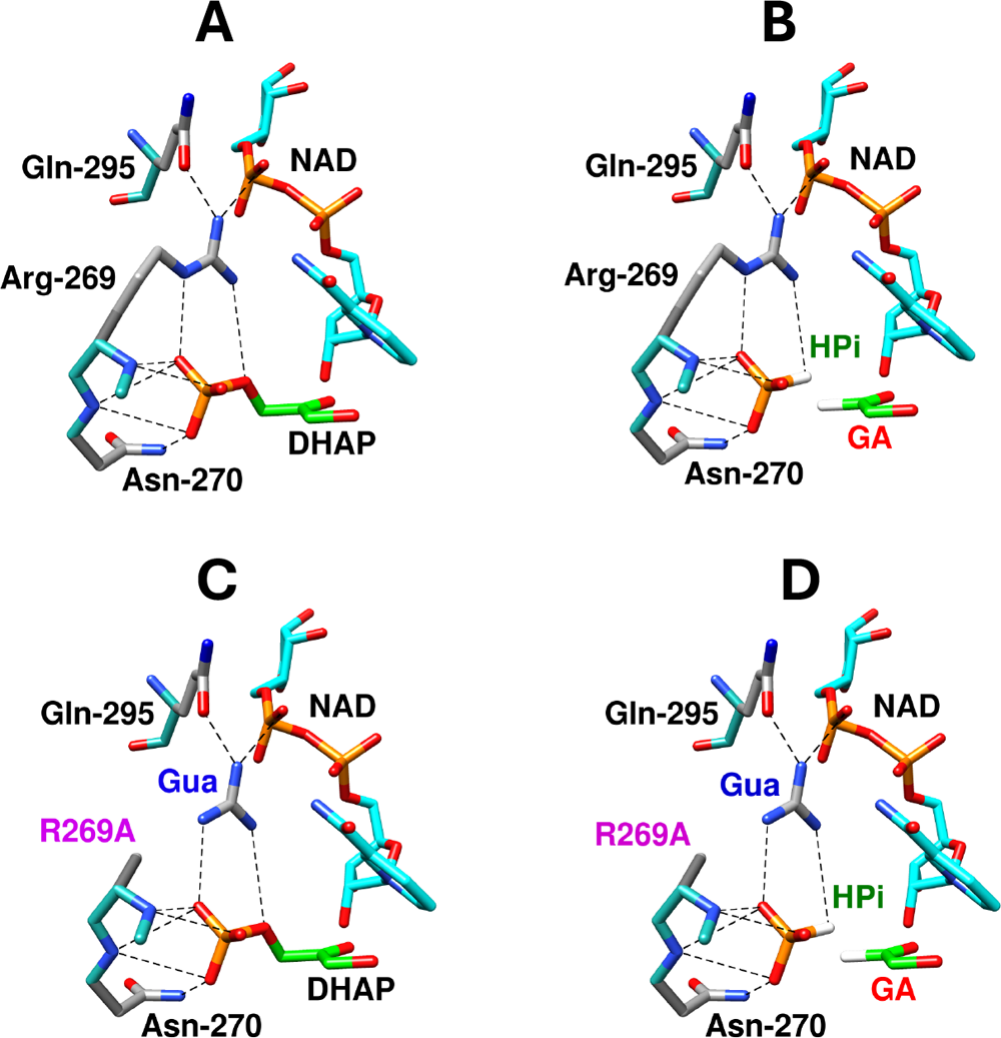
Representations of the X-ray crystal structure (Figure 14A, PDB 1WPQ) of the nonproductive Michaelis complex between wild-type *hl*GPDH, DHAP, and NAD^+^, and complexes of substrate pieces and/or enzyme pieces generated *in silico* by deletion of the relevant covalent linkage(s) from Figure 14A while maintaining a fixed position for the remaining atoms: (B) the complex between GA and HP_i_ for dianion-activated hydride transfer to GA; (C) the complex between R269A *hl*GPDH, DHAP, and Gua^+^ for rescue of the R269A variant; (D) the complex between R269A *hl*GPDH, GA, HP_i_, and Gua^+^ for rescue of R269A variant-catalyzed reduction of GA by HP_i_ and Gua^+^. Reproduced with permission from ref 143. Copyright 2016 American Chemical Society.

#### 4.4.2 Q295X Variants

The Q295 side chain is part of the [292-LNGQKL-297] loop that folds over the mouth of the enzyme active site at the nonproductive ternary complex (Figure 11). This second-shell active-site side chain forms a hydrogen bond to the first-shell R269 cation that holds the cation close to the DHAP dianion (Figures 12 and 14). The effect of Q295X (X = G, S, A, or N) substitutions on the kinetic parameters for *hl*GPDH-catalyzed reactions of the whole substrate DHAP and the pieces GA + HP_i_ were determined.^86^ The substitutions cause <4-fold decreases in (*k*_cat_/*K*_d_)_GA_ for wild-type *hl*GPDH-catalyzed reduction of GA and in *k*_cat_ for reduction of DHAP. This provides evidence that both reactions are catalyzed by the active, closed enzyme **E**_**C**_, whose catalytic activity is not strongly affected by Q295X substitutions

The Q295X substitutions cause large decreases, respectively, in (*k*_cat_/*K*_d_)_DHAP_ and *k*_cat_/*K*_GA_ *K*_HPi_ for *hl*GPDH-catalyzed reduction of the whole substrate DHAP and for dianion activated reduction of GA. The decrease in (*k*_cat_/*K*_d_)_DHAP_ is due mainly to an increase in *K*_m_ for DHAP so that the Q295X substitutions affect mainly the DHAP binding energy expressed at the substrate Michaelis complex. The observation that Q295X substitutions cause up to a 2.7 kcal/mol decrease in Δ*G*_HPi_^†^ for transition state stabilization by the phosphite dianion activator (eq 2 for Scheme 4) shows that the side chain functions to optimize dianion interactions in catalysis of the reaction of the whole substrate DHAP and the substrate pieces GA + HP_i_.^86^

Figure 15 shows the linear logarithmic correlation, with slope of 1.1, between the third-order rate constants *k*_cat_/*K*_GA_ *K*_HPi_ for reactions of the substrate pieces GA + HP_i_ and the second-order rate constant (*k*_cat_/*K*_m_)_DHAP_ for reactions of DHAP catalyzed by wild-type, Q295X and K120A variants of GPDH. The slope of close to 1.0 demonstrates that these substitutions result in similar changes in the stability of the transition state for the catalyzed reactions of the substrate pieces and whole substrate, so that severing the covalent connection at the whole substrate does not affect the interactions with the substituted side chains.^89^ The results are consistent with a model where the dianion binding energy provides similar stabilization of the active, closed form of *hl*GPDH for catalysis of the hydride transfer reaction of DHAP and of the substrate pieces GA + HP_i_.

**Figure 15:**
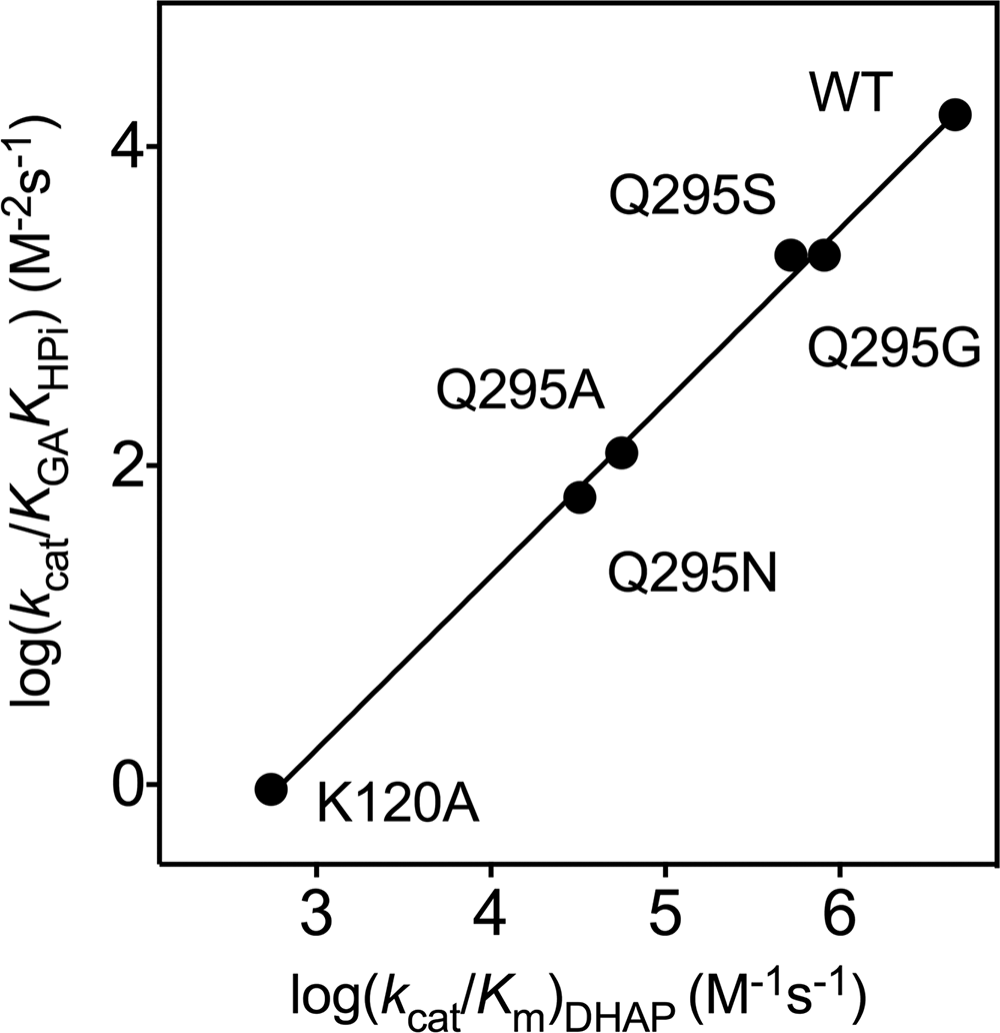
Linear logarithmic correlation, with a slope of 1.1, between the third-order rate constants *k*_cat_/*K*_GA_ *K*_HPi_ for phosphite dianion activated reduction of GA by NADH and the second-order rate constant (*k*_cat_/*K*_m_)_DHAP_ for direct reduction of DHAP catalyzed by wild-type, Q295X and K120A variants of *hl*GPDH. Reproduced with permission from ref 87. Copyright 2019 American Chemical Society.

The values of *k*_cat_/*K*_m_ for reduction of DHAP catalyzed by wild-type and variants of *hl*GPDH define the variant cycles for Figure 16A,B.^87^ The first cycle shows that the R269 side chain is required for the observation of a 2.5 kcal/mol destabilizing effect for the Q295A substitution. The first Q295A substitution reduces transition state stabilization by R269 from 9.1 to 5.3 kcal/mol. When R269 is absent the Q295 side chain destabilizes the transition state by 1.2 kcal/mol. A correction for the 1.2 kcal/mol destabilizing interaction of the Q295 side chain at the R269A variant gives a value of (9.1–1.2–2.4) ≈ 5.5 kcal/mol for the intrinsic transition state stabilization by the R269 side chain at wild-type GPDH, where 9.1 kcal/mol is the observed effect of the R269A substitution^87^ and 2.4 is the contribution of weakening of the K120 side chain interaction to the observed effect (Figure 13). The second cycle shows that the first K120A or Q295A substitution results in a 1 kcal/mol increase in the effect of the second substitution on the stability of the hydride-transfer transition state, so that the first K120A or Q295A substitution has the effect of tightening the interactions of the remaining side chain with the hydride transfer transition state.

**Figure 16:**
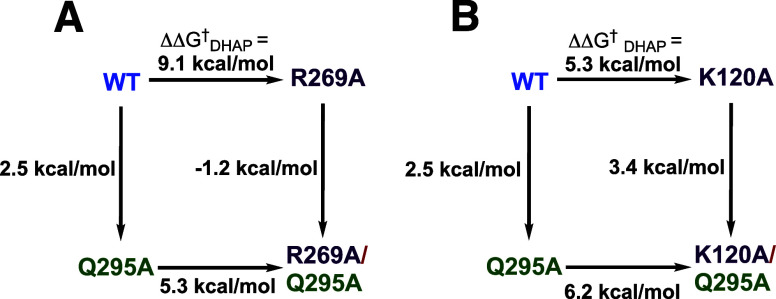
Variant cycles that illustrate the effect ΔΔ*G*_DHAP_^†^ of consecutive substitutions of amino acid side chains on the activation barrier Δ*G*_DHAP_^†^ for *hl*GPDH-catalyzed reduction of DHAP by NADH. The values of ΔΔ*G*_DHAP_^†^ were calculated from the ratio of the values of *k*_cat_/*K*_m_ for the parent and variant enzyme-catalyzed reactions.

#### 4.4.3 N270A Variant

The hydrogen bond between the substrate phosphodianion and the conserved N270 amide side chain (Figure 12) should stabilize the transition state for wild-type *hl*GPDH-catalyzed hydride transfer by *ca* 2–3-kcal/mol, but the N270A substitution results in a larger 5.6 kcal/mol transition state destabilization.^144^ The total effect is divided between a 3.6 kcal/mol destabilization of the ground-state Michaelis complex (*K*_m_ effect) and a 2.0 kcal/mol increase in the barrier for conversion of this complex to the hydride transfer transition state (*k*_cat_ effect).^144^

The N270A substitution also results in a 2.2 kcal/mol stabilization of the transition state for wild-type *hl*GPDH-catalyzed reduction of the truncated substrate GA, a decrease from 10.8 to 3.1 kcal/mol in the phosphodianion binding energy utilized to stabilize the transition state for hydride transfer to DHAP (Δ*G*_Pi_^†^ eq 4), and elimination of phosphite dianion activation of wild-type *hl*GPDH-catalyzed reduction of GA.^144^ These results show that the modest N270A substitution refashions the active-site structure to reduce the total dianion stabilization of the transition state for *hl*GPDH-catalyzed reduction of DHAP, enhance stabilization of the transition state for direct reduction of GA and eliminate phosphite dianion activation of this reaction.

4 $$\Delta G_{\mathrm{Pi}}^{†}=-\mathrm{RT}\ln\left(\frac{{\left(k_{\mathrm{cat}}/K_{m}\right)}_{\mathrm{DHAP}}}{{\left(k_{\mathrm{cat}}/K_{d}\right)}_{\mathrm{GA}}}\right)$$

Binding of phosphite dianion to the N270A variant causes (*k*_cat_/*K*_m_)_GA_ for enzyme-catalyzed reduction of GA to decrease (Figure 17).^144^ The data for reactions at low [HPO_3_^2–^] (Figure 17, inset) were fit to eq 5 derived for Scheme 12 using the following parameters: (*k*_cat_/*K*_m_)_GA_ = 2.0 M^–1^ s^–1^, (*k*_cat_/*K*_m_)_E•HPi_ = 1.3 M^–1^ s^–1^ and *K*_d_ = 6.0 × 10^–5^ M. By comparison, values of (*k*_cat_/*K*_m_)_GA_ = 0.05 M^–1^ s^–1^, *K*_d_ = 7.0 × 10^–2^ M and (*k*_cat_/*K*_m_)_E•HPi_ = 1100 M^–1^ s^–1^ were reported for phosphite dianion activation of wild-type *hl*GPDH-catalyzed reduction of GA.^144^ These kinetic parameters show that the N270A substitution modifies the GA and HP_i_ binding sites to give an increase in the reactivity of GA for unactivated enzyme-catalyzed hydride transfer and in phosphite dianion affinity for binding at either a modified, or distinct, dianion site that weakly deactivates *hl*GPDH for catalysis of reduction of GA.

5 $${\left(\frac{k_{\mathrm{cat}}}{K_{m}}\right)}_{\mathrm{obs}}=\left(\frac{K_{d}}{K_{d}+\left[{\mathrm{HPO}}_{3}^{2-}\right]}\right){\left(\frac{k_{\mathrm{cat}}}{K_{m}}\right)}_{E}+\left(\frac{\left[{\mathrm{HPO}}_{3}^{2-}\right]}{K_{d}+\left[{\mathrm{HPO}}_{3}^{2-}\right]}\right){\left(\frac{k_{\mathrm{cat}}}{K_{m}}\right)}_{E•{\mathrm{HP}}_{i}}$$

**Figure 17:**
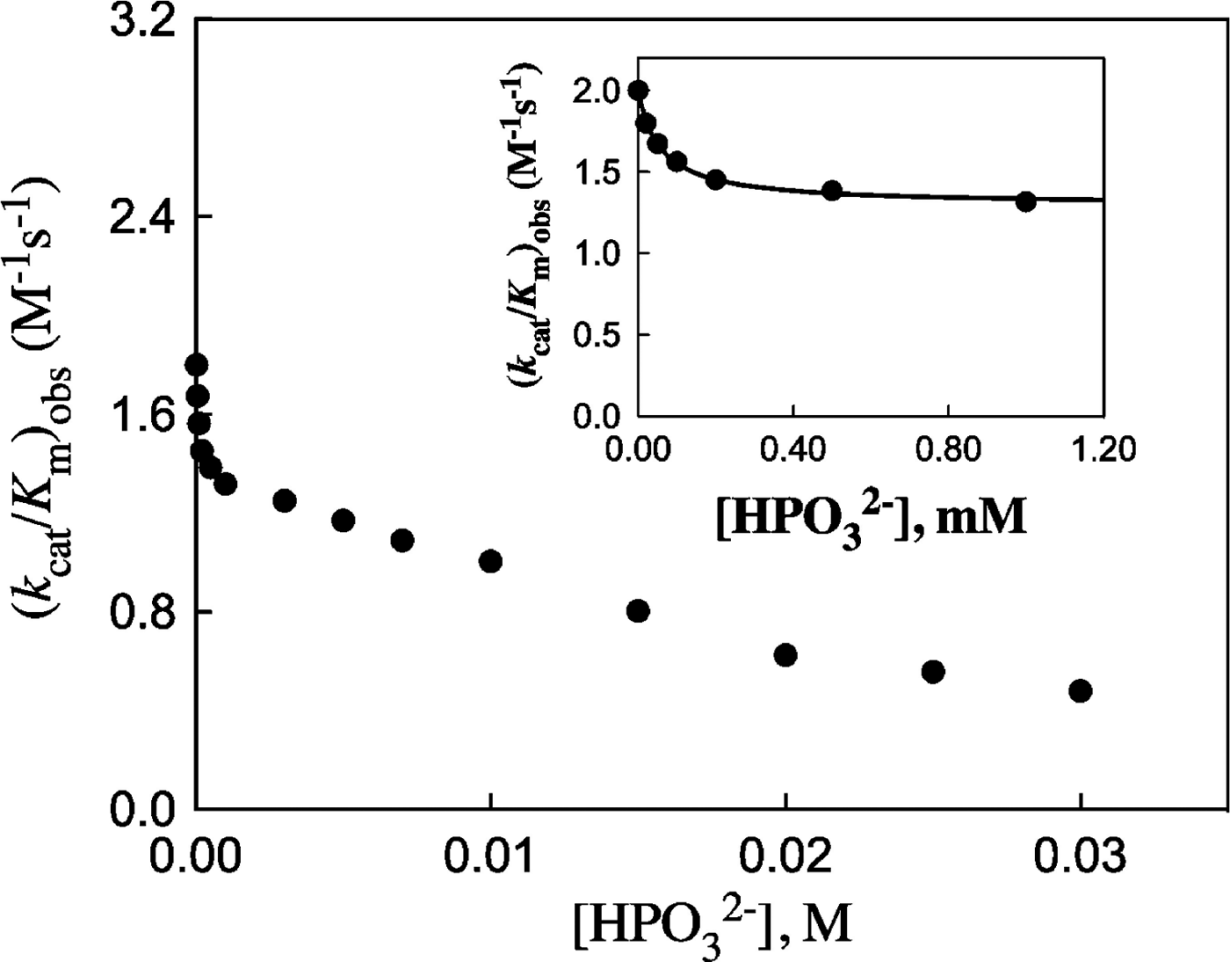
Effect of increasing [HPO^2–^] on the observed second-order rate constant for N270A *hl*GPDH-catalyzed reduction of GA at 25 °C and pH 7.5. The data at low [HPO^2–^] show a good fit to eq 5 derived for Scheme 12.^144^ Reproduced with permission from ref 144. Copyright 2016 American Chemical Society.

**Scheme 12:**
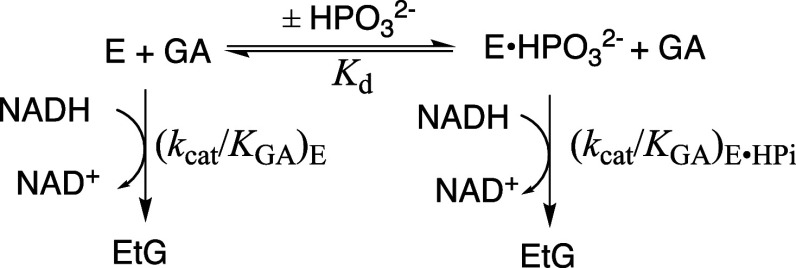
Kinetic Mechanism for N270A *hl*GPDH-Catalyzed Reduction of GA by NADH to Give Ethylene Glycol (EtG)

The variant cycle (Figure 18) shows that the N270A side chain substitution reduces ΔΔ*G*_DHAP_^†^ for the R269 substitution from 9.1 to 5.9 kcal/mol (3.2 kcal/mol). The N270A substitution causes a similar 3.6 kcal/mol destabilization of the Michaelis complex to DHAP (see above). We speculate that the N270A substitution induces a change in protein structure that favors a DHAP binding conformation where there are minimal ground-state interactions with the R269 side chain. These R269 interactions, that now develop only at the hydride transfer transition state for the N270A variant-catalyzed reaction, are eliminated by the second R269A substitution.

**Figure 18:**
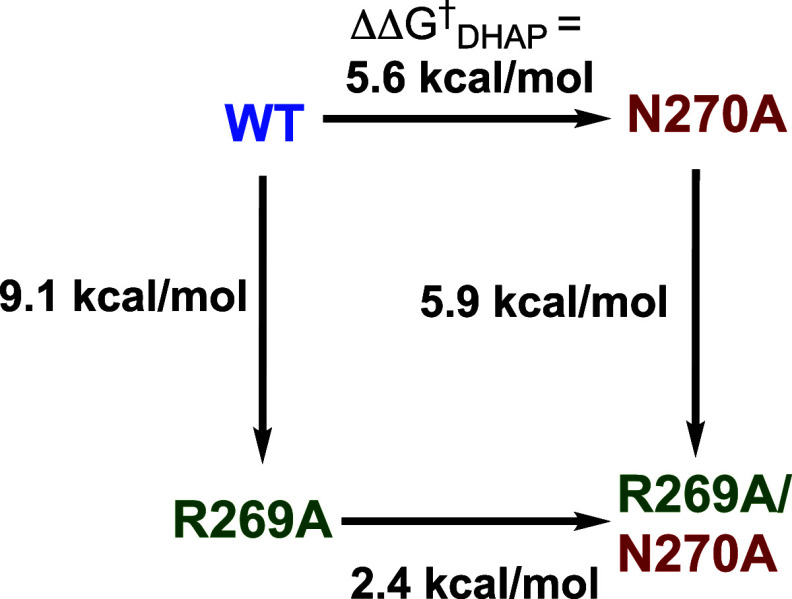
Variant cycle that illustrates the effect (ΔΔ*G*_DHAP_^†^) of consecutive substitutions of two amino acid side chains on the activation barrier Δ*G*_DHAP_^†^ for *hl*GPDH-catalyzed reduction of DHAP by NADH. The values of ΔΔ*G*_DHAP_^†^ were calculated from the ratio of the values of *k*_cat_/*K*_m_ for the parent and variant enzyme-catalyzed reactions.

#### 4.4.4 K120A, K204A, and D260G Variants

The K120 side chain is held close to the substrate carbonyl by an ion pair to D260 (Figure 19). This side chain cation may either provide formal Brønsted acid catalysis of hydride transfer to DHAP that is concerted with protonation of the carbonyl oxygen, or catalysis of stepwise hydride transfer to DHAP by formation of a hydrogen bond between the protonated K120 side chain and substrate carbonyl followed by proton transfer to the oxyanion reaction intermediate (Scheme 13). The following favor utilization of the K120 side chain in the stepwise reaction mechanism. (1) There is a relatively small driving force for concerted Brønsted general acid catalysis that avoids an oxyanion intermediate with a p*K*_a_ similar to p*K*_a_ = 13.6 for oxygen deprotonation of hydroxyacetone.^109,145^ (2) The driving force for the stepwise reaction mechanism through an oxyanion reaction intermediate is similar to that for TIM-catalyzed deprotonation of DHAP through the enediolate oxyanion intermediate.^32–34^ (3) There is no obvious role for the K204 side chain cation in a mechanism where K120 provides concerted Brønsted acid catalysis of hydride transfer from NADH; however, K204 may function to provide additional electrostatic stabilization of a stepwise oxyanion reaction intermediate (Scheme 13). (4) The many examples of stabilization of oxyanion reaction intermediates by multiple hydrogen bond donors at oxyanion holes provides precedent for a reaction mechanism where the K120 and K204 side chains are used to stabilize the stepwise oxyanion intermediate for direct hydride transfer to DHAP.^146–151^

**Figure 19:**
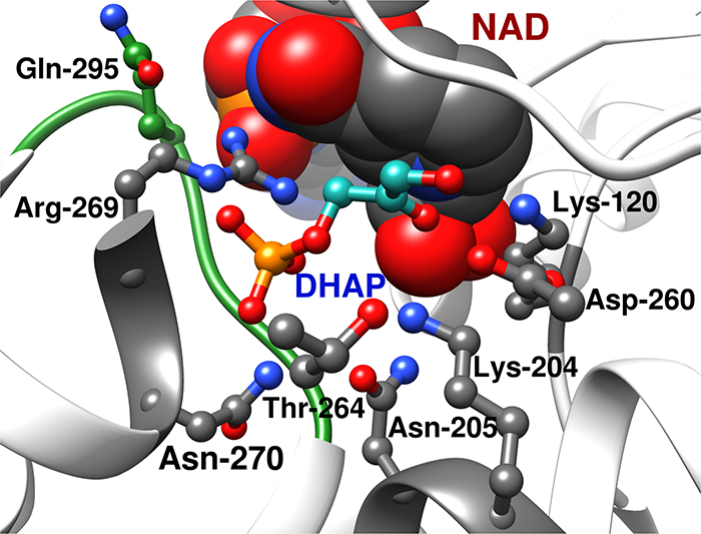
Representation of the X-ray crystal structure of the nonproductive ternary complex between *hl*GPDH, DHAP, and NAD^+^ (PDB 6E90)^87^ that shows the K120, D260, and K204 side chains. These side chains are positioned to participate in protonation of the oxyanion intermediate of stepwise hydride transfer from NAD^+^ to DHAP (Scheme 13). Reproduced with permission from ref 87. Copyright 2019 American Chemical Society.

**Scheme 13:**
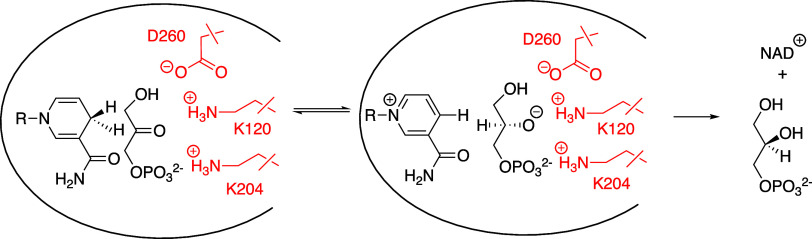
*hl*GPDH-Catalyzed Hydride Transfer to Form an Alkoxide Anion Intermediate Stabilized by Interactions with K120 and K204 Side Chain Cations^152^

Support for the stepwise reaction mechanism (Scheme 13) is provided by the following results from mutagenesis studies.

(1) Single K120A and K204A substitutions result, respectively, in 5.3,^87^ and 3.1,^152^ kcal/mol increases in the activation barrier Δ*G*_DHAP_^†^ for *hl*GPDH-catalyzed hydride transfer (Figure 20A). The first K120A or K204A substitution at wild-type *hl*GPDH results in a 1.1 kcal/mol decrease in the effect of the second substitution on the stability of the hydride-transfer transition state.^152^ There results are consistent with a stepwise mechanism where (i) Both side chain cations function to stabilize the O-2 oxyanion reaction intermediate (Scheme 13). (ii) Substitution of one side chain reduces the stabilizing interaction of the second side chain.

**Figure 20:**
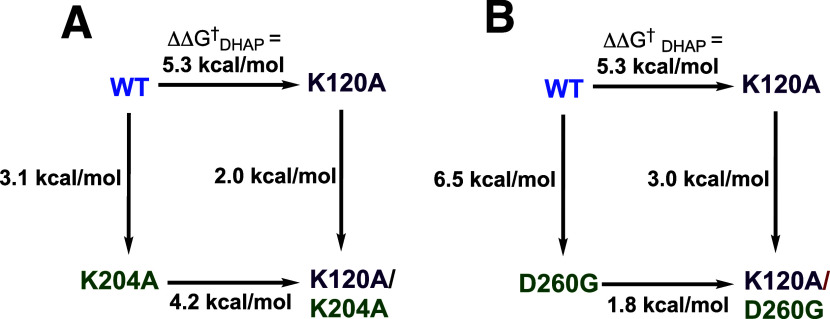
Variant cycles that illustrate the effect (ΔΔ*G*_DHAP_^†^) of consecutive substitutions of two amino acid side chains on the activation barrier Δ*G*_DHAP_^†^ for *hl*GPDH-catalyzed reduction of DHAP by NADH. The values of ΔΔ*G*_DHAP_^†^ were calculated from the ratio of the values of *k*_cat_/*K*_m_ for the parent and variant enzyme-catalyzed reactions.

(2) More than 50% of the 6.5 kcal/mol effect of the D260G substitution on Δ*G*_DHAP_^†^ is eliminated by an initial K120A substitution (Figure 20B). This highlights the importance of D260 in positioning K120 to provide for optimal transition state stabilization.^87^

(3) The K120 side chain is roughly equidistant from O-1 (3.0 Å) and O-2 (2.6 Å) of DHAP, while the K204 side chain interacts only with O-2 (2.8 Å; Figure 21).^87^ This provides evidence that the difference between the 5.3 and 3.1 kcal/mol effects of K120A and K204A substitutions on Δ*G*_DHAP_^†^ (Figure 20A) is due to the additional interaction of K120 with the C-1 hydroxyl that is lost for the K120A variant.^87,152^ The larger effect of the K120A (*K*_m_ = 1.6 mM)^87^ compared with the K204A substitution (*K*_m_ = 0.023 mM)^152^ on *K*_m_ = 0.052 mM for wild-type *hl*GPDH provides evidence that a 2 kcal/mol stabilizing interaction between the K120 side chain and the C-1 hydroxyl of DHAP accounts for the difference between the 5.3 and 3.1 kcal/mol effects of these substitutions on Δ*G*_DHAP_^†^ (Figure 20A).

**Figure 21:**
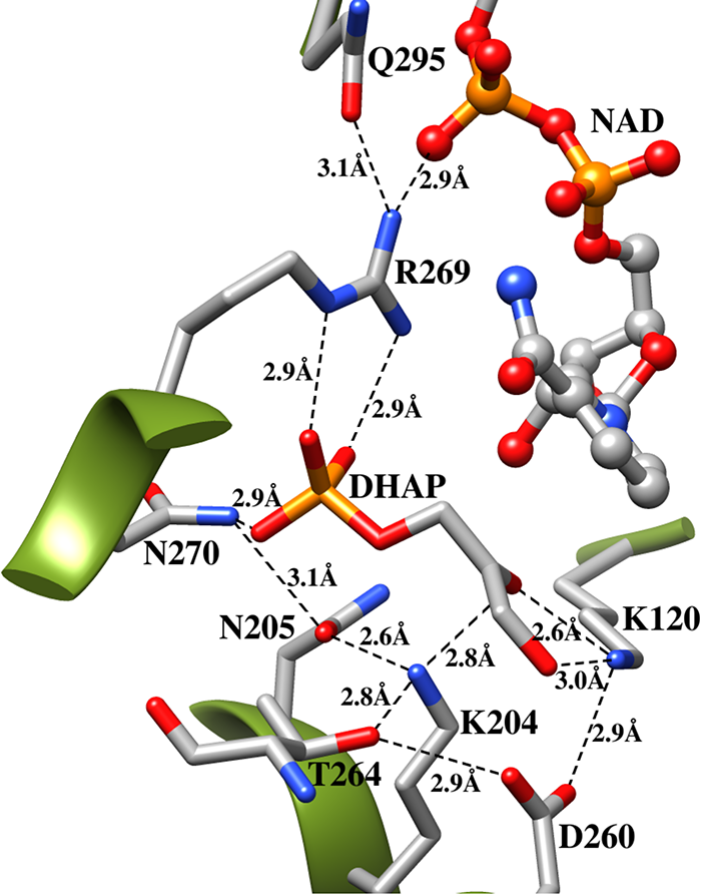
Representation of a portion of the X-ray crystal structure of the active site of *hl*GPDH (PDB 6E90) that shows the protein side chains that interact with the substrate C-1 and C-2 oxygens of DHAP. The K120 side chain cation interacts with the C-2 carbonyl oxygen (2.6 Å), the C-1 hydroxyl (3.0 Å) and the D260 carboxylate (2.9 Å). The K204 side chain interacts with the C-2 carbonyl oxygen (2.8 Å), the T264 hydroxyl (2.8 Å), and the N205 amide oxygen (2.6 Å).

(4) The similar *ca* 300-fold effects of the K120A^87^ and the K204A^152^ substitutions on *k*_cat_ = 240 s^–1^ for wild-type *hl*GPDH-catalyzed reduction of DHAP are consistent with similar *ca*. 3 kcal/mol stabilizing side chain interactions with the incipient transition state C-2 oxyanion.

### 4.5 Effect of Side Chain Substitutions on Enzyme Specificity

The induced-fit pathway for GPDH (Scheme 1) provides a mechanism for the enzyme to achieve a high specificity for binding the substrate dianion at the transition state for hydride transfer to DHAP. Figure 22 shows that the enzyme specificity for catalysis of reduction of DHAP compared to S = GA or S = acetaldehyde (AcA) [log (*k*_cat_/*K*_m_)_DHAP_/(*k*_cat_/*K*_m_)_S_] is sharply reduced by K204A and K120A/K204A substitutions which eliminate side chains that stabilize negative charge at the transition state for hydride transfer to DHAP (Scheme 13). This provides strong evidence that the phosphodianion-driven protein conformational change for the reaction of DHAP promotes optimal stabilization of the transition state for GPDH-catalyzed reduction by interactions of K204 and K120 side chains with the C-2 oxygen.

**Figure 22:**
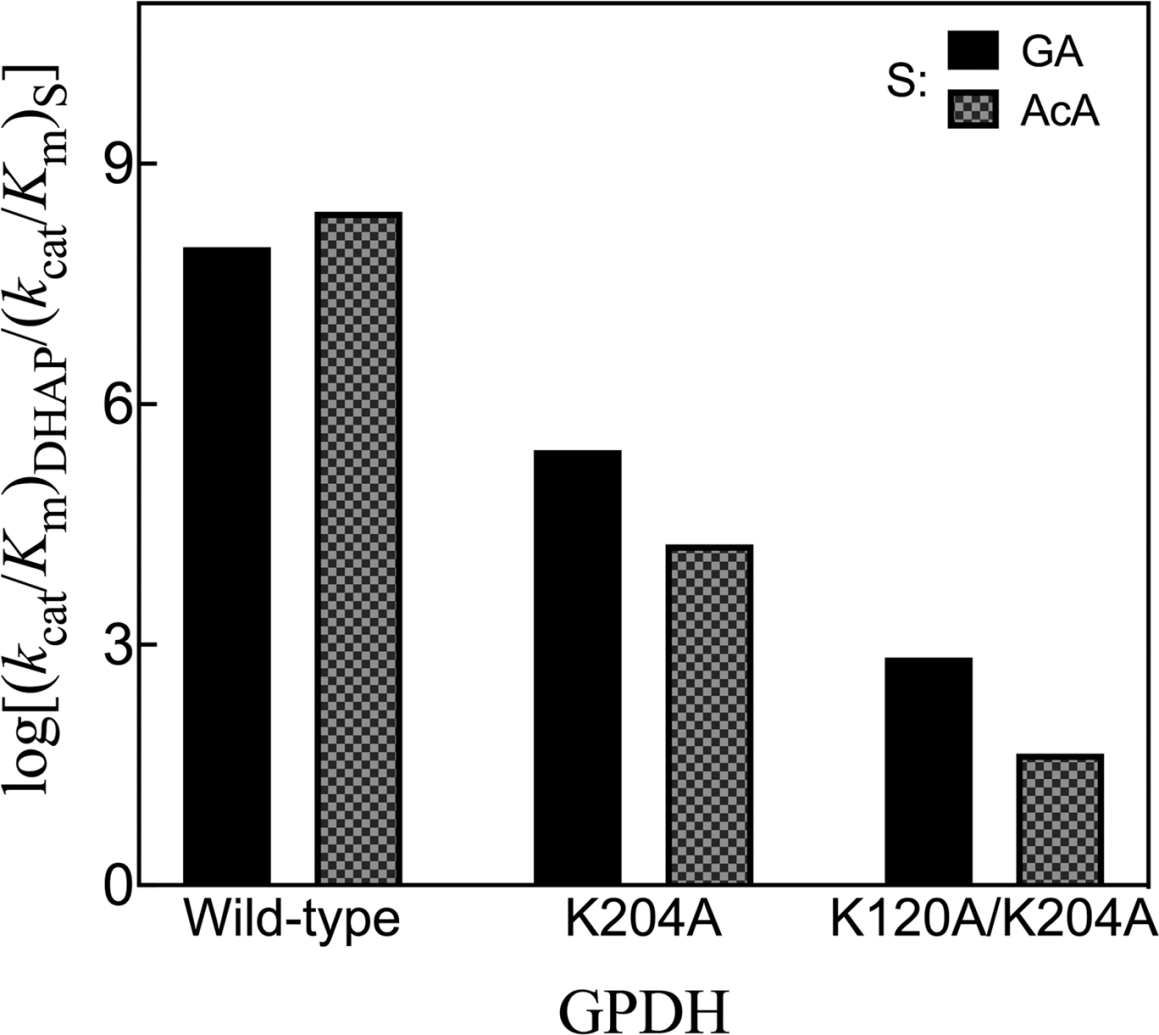
Effect of K204A and K120A substitutions on the selectivity [log (*k*_cat_/*K*_m_)_DHAP_/(*k*_cat_/*K*_m_)_AcA_] and [log (*k*_cat_/*K*_m_)_DHAP_/(*k*_cat_/*K*_m_)_GA_] for *hl*GPDH-catalyzed reduction of the physiological substrate DHAP and the small substrates GA and AcA. Reproduced with permission from ref 152. Copyright 2021 American Chemical Society.

A comparison of kinetic parameters *k*_cat_/*K*_m_ and *k*_cat_/*K*_d_ *K*_HPi_ for unactivated and phosphite dianion activated *hl*GPDH-catalyzed reactions of GA and AcA shows that the hydroxyl at GA stabilizes the transition states for the unactivated reaction and phosphite dianion activated reaction, respectively, by 0.7 and 5.7 kcal/mol.^152^ The former is the small inductive −OH substituent effect.^109^ The larger effect for the activated reaction is due to the phosphite dianion driven protein conformational change, that tightens interactions of the K120 and K204 side chains with O-1 and O-2 of the dianion truncated substrate GA.

### 4.6 pH-Rate Profiles

The pH-rate profiles for (*k*_cat_/*K*_m_)_dianion_ for wild-type (◆) and K120A (▲) *hl*GPDH-catalyzed reduction of DHAP dianion and for *k*_cat_/*K*_m_ *K*_am_ for ethylammonium cation (EtNH_3_^+^) rescue (●) of the K120A variant-catalyzed reaction are presented in Figure 23.^153^ The Figure provides the fit of these data to equations derived for Scheme 14 where p­(*K*_a_)_a_ and p­(*K*_a_)_b_, respectively, are for the D260 and K204 side chains. Data for the wild-type enzyme were fit to an equation derived for Scheme 14A using values of p­(*K*_a_)_a_ = 4.4 and p­(*K*_a_)_b_ = 7.6, respectively. Data for the K120A variant were fit to the appropriate equation derived for Scheme 14B using p­(*K*_a_)_a_ = 5.0 and p­(*K*_a_)_b_ = 7.6. The data for rescue of the K120A variant by EtNH_3_^+^ were fit to an eq derived for Scheme 14C using p­(*K*_a_)_b_ = 7.7 for the K204 side chain.

**Figure 23:**
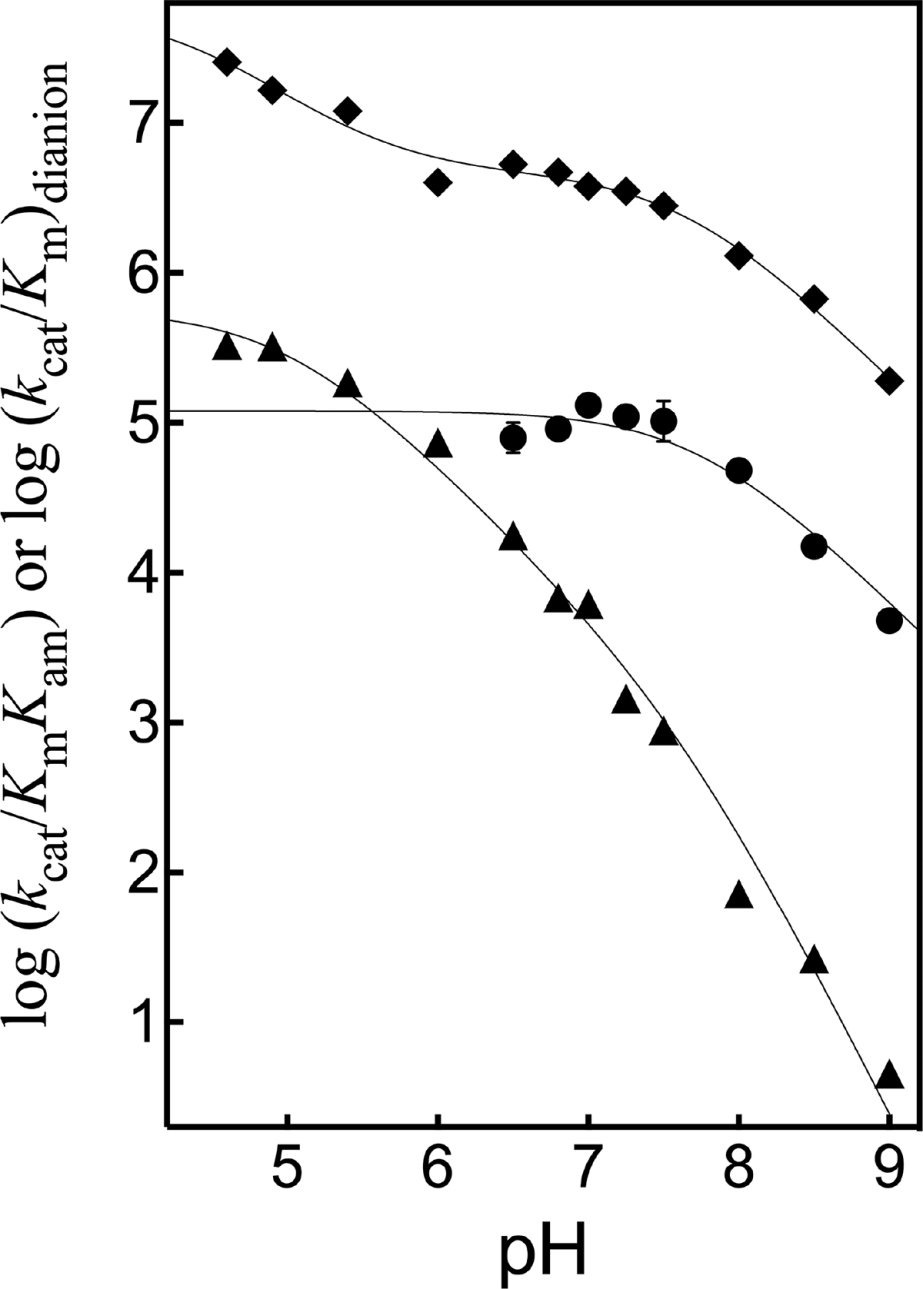
pH profiles for second-order rate constants (*k*_cat_/*K*_m_)_dianion_ for wild-type (◆) and K120A variant (▲) *hl*GPDH-catalyzed reduction of DHAP dianion by NADH, and for third-order rate constants (*k*_cat_ */K*_m_ *K*_am_) (●) for rescue of the K120A variant by EtNH_3_^+^. The Figure shows the fit of the experimental data to equations derived for Scheme 14A (◆), B (▲), and C (●). Reproduced with permission from ref 153. Copyright 2021 American Chemical Society.

**Scheme 14:**
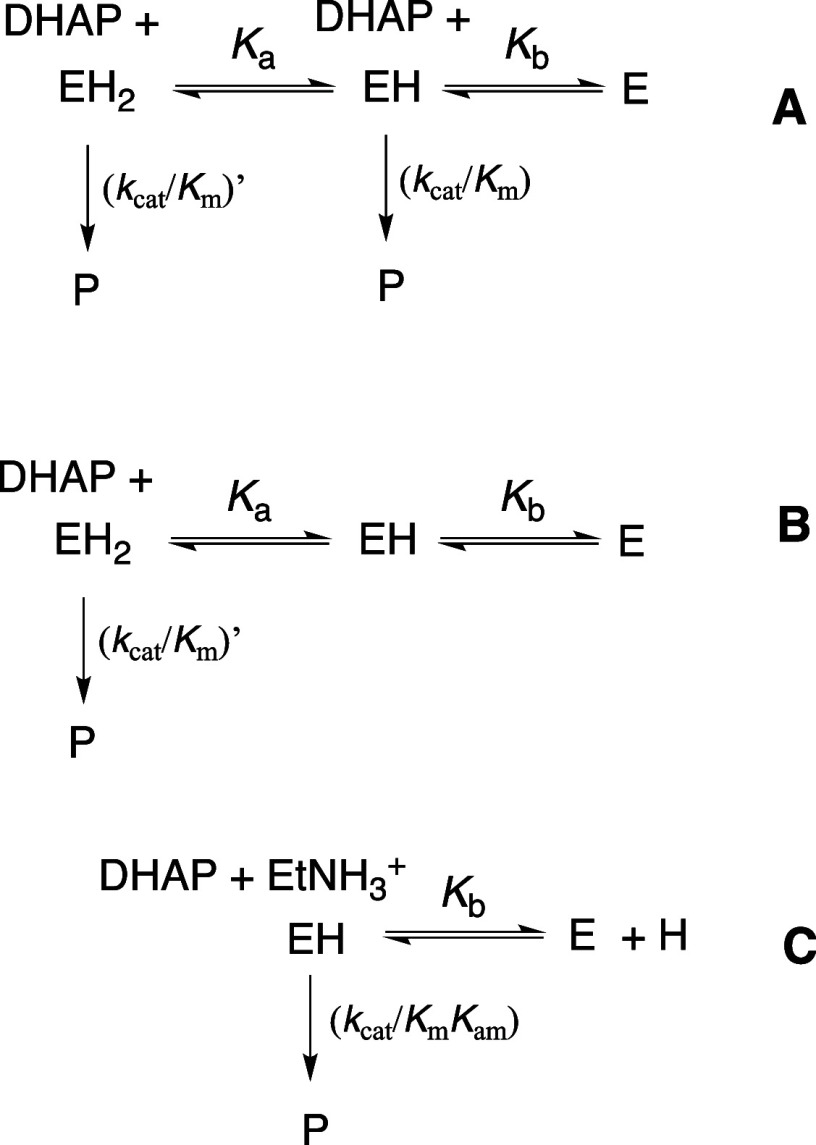
Kinetic Mechanisms for *hl*GPDH-Catalyzed Hydride Transfer Used to Fit Data from Figure 23 for Wild-Type and K120A Variant-Catalyzed Hydride Transfer, Where p­(*K*_a_)_a_ and p­(*K*_a_)_b_, Respectively, Are for the D260 and K204 Side Chains^a^

a (A) Wild-type *hl*GPDH-catalyzed reactions (◆). (B) K120A Variant-Catalyzed Reactions (▲). (C) Rescue of the K120A variant by EtNH_3_^+^ (●).

The pH-rate profiles from Figure 23 provide support for the following model: (1) The K120 side chain at wild-type *hl*GPDH remains fully protonated up to pH 9 due to stabilization of the side chain cation by the ion-pairing interaction with the D260 side chain. (2) Protonation of the D260 carboxylate at low pH enhances Brønsted acid catalysis at wild-type *hl*GPDH. (3) Protonation of K204 [p­(*K*_a_)_b_ = 7.6] and D260 [(p­(*K*_a_)_a_ = 5.0] side chains give rise to a slope of −2 for the profile for the K120A variant. (4) The K204 side chain has a similar p­(*K*_a_)_b_ = 7.6 for deprotonation at wild-type *hl*GPDH and at the K120 variant. (5) Rescue of the K120A variant by EtNH_3_^+^ requires that the K204 side chain be protonated with p­(*K*_a_)_b_ = 7.7.

### 4.7 Hydride Transfer Reaction Mechanism

Substrate dianion binding energy might be utilized to drive a protein conformational change to form a tunneling ready state that enables hydride transfer to DHAP by quantum-mechanical (QM) tunneling, without passage over a semiclassical reaction coordinate.^154,155^ Primary deuterium kinetic isotope effects (1° DKIEs) of *k*_H_/*k*_D_ = 2.4–2.9 on *k*_cat_/*K*_m_ were determined for unactivated and for dianion-activated *hl*GPDH-catalyzed hydride transfer from NADH to GA. These isotope effects lie at the bottom end of the range (*k*_H_/*k*_D_ = 2–7) for semiclassical hydride transfer.^138^ The 1° DKIEs are much smaller than observed for enzyme-catalyzed hydrogen transfer that occurs by QM tunneling, where tunnelling is favored for hydrogen compared to deuterium by the shorter QM wavelength for hydrogen.^156^ Similarly, the 1° DKIEs determined for hydride transfer to DHAP catalyzed by crippled variants of *hl*GPDH, at rates limited by the chemical barrier to hydride transfer, give nearly invariant 1° DKIEs on *k*_cat_/*K*_m_ that range from *k*_H_/*k*_D_ = 2.4–3.1 and which do not vary systematically over a 9.1 kcal/mol change in the reaction activation barrier.^157^ The 1° DKIE of *k*_H_/*k*_D_ = 1.5 for wild-type *hl*GPDH-catalyzed reduction of DHAP is smaller than the intrinsic value determined for variant enzymes, because the hydride transfer is not cleanly rate determining for the wild-type enzyme.^157^

Results from computational studies that model the observed barrier for enzyme-catalyzed hydride transfer are consistent with QM tunneling of transferred −H and −D. The tunneling occurs at close to the top of the semiclassical free energy barrier to hydride transfer, and results in only small (incidental) decreases in barrier height that give rise to small increases in the 1° DKIE.^158,159^ These small effects of tunneling on the barrier height to enzyme-catalyzed hydride do not provide a significant driving force for evolution of active sites that stabilize tunneling ready states.^138,157^

### 4.8 Network of Active-Site Amino Acid Side Chains

The dianion-driven enzyme conformational change at *hl*GPDH creates an extended network of hydrogen bonding interactions, where the K120, R269, N270, and K204 are first shell side chains that interact directly with the substrate DHAP, and the Q295, N205, D260, and T264 side chains hold the first-shell side chains at their active positions. (Figure 12). This side chain network is maintained in the efficient rescue of R269A, K120A, and K120A/R269A variants by added Gua^+^ (R269A),^141,143^ EtNH_3_^+^ (K120A)^87^ or by a mixture of Gua^+^ and EtNH_3_^+^ (K120A/R269A,^160^ Figure 24). There is no rescue of the K204A variant by EtNH_3_^+^, because either the pocket for the excised side chain is not accessible to EtNH_3_^+^ or the enzyme structure for the variant is perturbed by a protein conformational change.^152^

**Figure 24:**
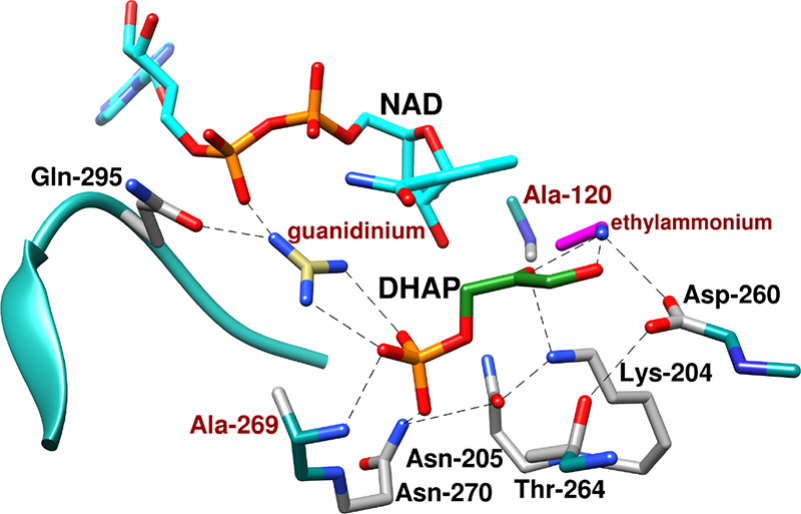
Representation of the X-ray crystal structures of the complex of K120A/R269A *hl*GPDH to NAD^+^, DHAP, Gua^+^, and EtNH_3_^+^, where the cations act to rescue the activity for the variant enzyme.^160^ This complex was generated *in silico*, starting with the X-ray crystal structure of the nonproductive complex between *hl*GPDH, DHAP, and NAD^+^ (PDB 6E90) and then deleting of the relevant side chain covalent linkages while maintaining a fixed position for the remaining atoms at the Michaelis complex. Reproduced with permission from ref 160. Copyright 2020 American Chemical Society.

The sum of the effect of six side chain substitutions on Δ*G*_DHAP_^†^ for *hl*GPDH-catalyzed hydride transfer: Q295A, 2.5 kcal/mol;^86^ R269A, 9.1 kcal/mol;^143^ N270A, 5.6 kcal/mol;^144^ K120A, 5.3 kcal/mol;^87^ D260G, 6.5 kcal/mol;^87^ and K204A, 3.1 kcal/mol^152^ is 32 kcal/mol. This sum is >2-fold larger than the estimated 15 kcal/mol total transition state stabilization by the protein catalyst.^157,161^ These results provide evidence that the loss of direct interactions of single side chains with the reaction transition state has the effect of weakening transition state interactions by other networked side chains. This is illustrated by the variant cycles from Figures 13, 16A, 17, 18, and 20. An interesting exception is Figure 16B, where the first K120A or Q295A substitution results in a 1.1 kcal/mol increase in the effect of the second substitution on transition state stability.

We suggest that *hl*GPDH holds the DHAP substrate and the NADH cofactor at positions that minimize the barrier to enzyme-catalyzed hydride transfer and that this requires a tight network of protein–ligand interactions to optimize the positioning of the hydride acceptor and donor.^47,162,163^ The present results are consistent with the existence of this network, whose function is impaired by substitution of any of the participating amino acid side chains so that the sum of the effects of substitution of these side chains exceeds the catalytic advantage provided by the side chain network.

### 4.9 A Role for Cofactor Binding Energy

*hl*GPDH catalyzes slow hydride transfer from G3P to the truncated cofactor nicotinamide riboside (NR). The transition state for *hl*GPDH-catalyzed hydride transfer from G3P to NR is stabilized by the binding of adenosine 5́-diphosphate (ADP), adenosine 5′-monophosphate (AMP), and ribose 5-phosphate (R5P) activator cofactor fragments (Figure 25).^20^ We have reported similar results for the hydride transfer reactions of the whole NAD^+^ and the truncated NR cofactors catalyzed by formate dehydrogenase.^19^

**Figure 25:**
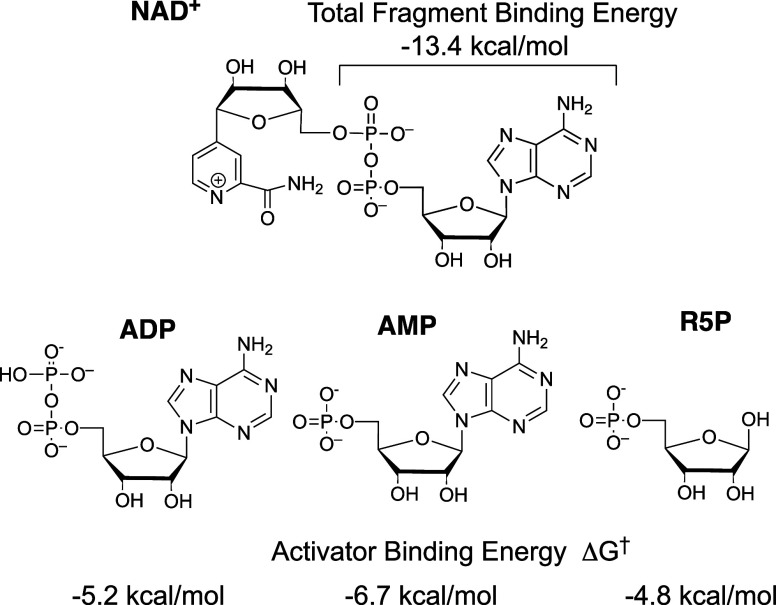
(top) The stabilization of the transition state for *hl*GPDH-catalyzed hydride transfer to NR by the ADP moiety at the NAD^+^ cofactor. (bottom) The stabilization of the transition state for hydride transfer from G3P to NR by 1.0 M activator.^20^

The binding of NAD^+^ to the binary E•SO_4_^2–^ complex causes the [292-LNGQKL-297] loop to move by 5 Å toward the enzyme-bound cofactor.^20,135^ This motion gives rise to at least two interactions that activate *hl*GPDH for catalysis of hydride transfer (Figure 26). (1) The K296 side chain cation moves to within 5 Å of the purine ring; this is within the range of distances observed for lysine cation-pi interactions at proteins.^164–166^ (2) The backbone amide of K296 moves into position to hydrogen bond to the α-adenosyl phosphate of NAD^+^ (Figure 26). In addition, the R269 side chain cation forms ion-pairing interactions with the DHAP phosphodianion and the NAD^+^ α-adenosyl phosphate (Figure 12). The extended interactions of the R269 side chain, which connect the DHAP and cofactor binding sites, are consistent with a conformational change that is driven by cooperative interactions between these two binding sites.

**Figure 26:**
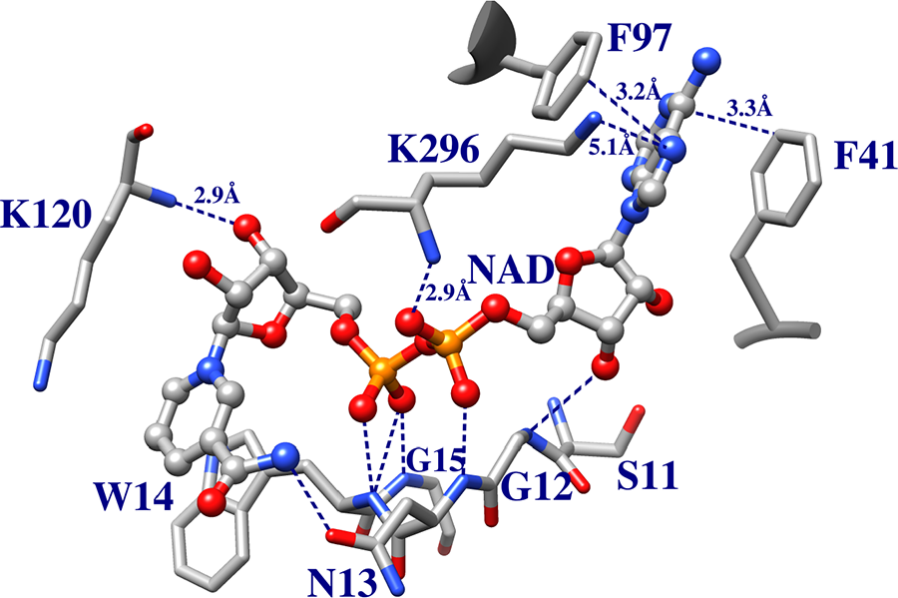
Representation of the X-ray crystal structure for the nonproductive E•NAD^+^•DHAP complex [PDB 6E90] for GPDH that highlights interactions between the protein and the bound cofactor. These include interactions of side chains from the highly conserved [10-GxGxxG-15] Rossman-fold motif.^167,168^ Reproduced with permission from ref 20 Copyright 2024 American Chemical Society.

The [292-LNGQKL-297] loop folds over the mouth of the active site of *hl*GPDH at the ternary E•NAD^+^•DHAP complex (Figure 11). This positions the Q295 and K296 loop side chains to interact, respectively, with the DHAP phosphodianion and the cofactor adenine ring (Figure 27). The [292-LNGQKL-297] loop sequence is conserved at GPDH from human, rabbit, rat, mouse, fish, and *Drosophila melanogaster*,^20^ but there is considerable variation in the sequence for GPDH from organisms at lower positions on the evolutionary tree.^140^ This suggests that the loop sequence from higher organisms provides optimal loop function in catalysis of hydride transfer by enabling interactions of side chains with both the phosphodianion (Q295) and the AMP (K296) activators. We are testing this hypothesis by examining the effect of substitutions of these side chains on enzyme activity for catalysis of the reactions of whole substrates and the substrate pieces.

**Figure 27:**
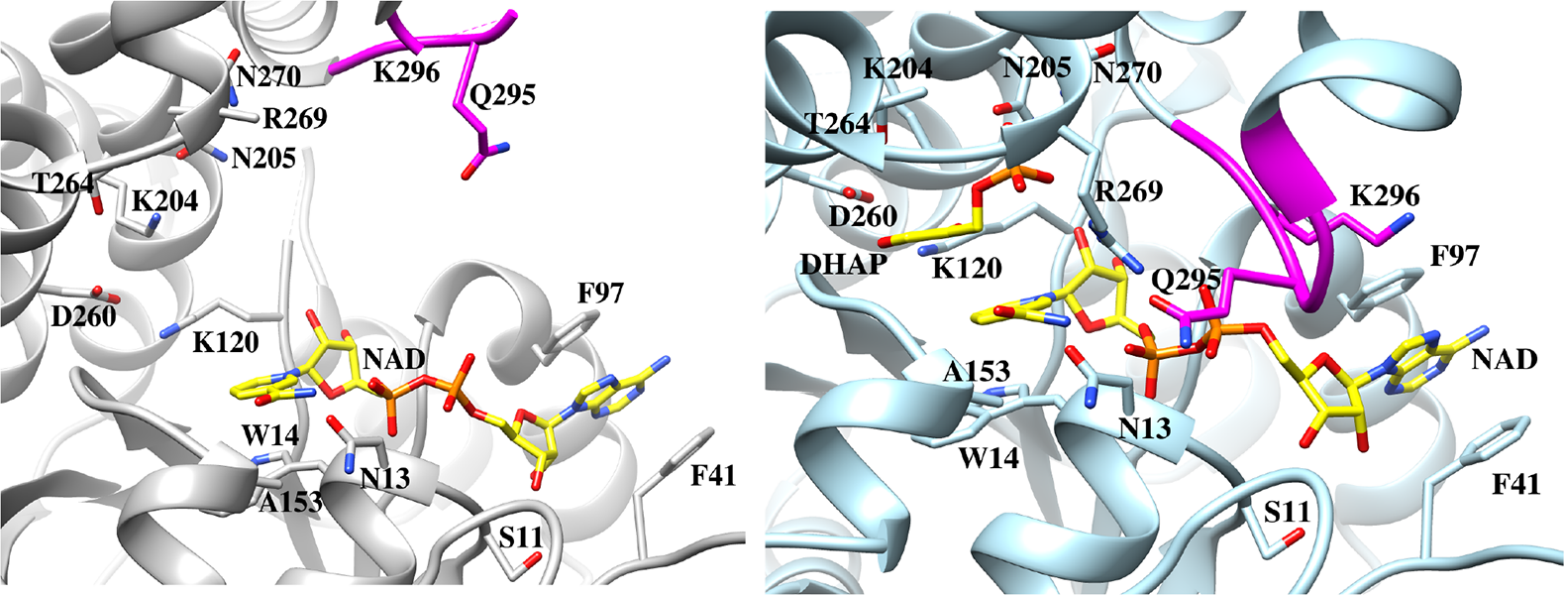
Representations of X-ray crystal structures for GPDH with the [292-LNGQKL-297] loop shaded magenta. (left) The open protein conformation for the binary E•NAD^+^ complex [PDB 6E8Z]. The K296 side chain is not visible. (right) The closed conformation for the nonproductive ternary E•NAD^+^•DHAP complex [PDB 6E90]. The Q295 and K296 side chains are positioned to interact, respectively, with the R269 side chain and the adenine ring of NAD^+^. There is an additional interaction between the backbone amide of K296 and the α-adenosyl phosphate of NAD^+^ (Figure 26). Reproduced with permission from ref 20. Copyright 2024 American Chemical Society.

### 4.10 Summary

The results from studies on variants of *hl*GPDH provide support for a model where the 12 kcal/mol substrate phosphodianion binding energy is utilized to stabilize a network of active-site amino acid side chains that provide a template for the transition state for *hl*GPDH-catalyzed hydride transfer. The dominant interaction in this network is the ion-pair between the R269 side chain and the substrate dianion. The R269A substitution causes a 9.1 kcal/mol increase in the activation barrier to hydride transfer due to loss of the stabilizing ionic interaction, a weakening of the interaction with the K120 side chain (Figure 13), and the movement of the Q295 side chain to a position that destabilizes the transition state by 1.2 kcal/mol (Figure 16A). The efficient small molecule rescue of K120A, R269A, and K120A/R269A variants demonstrates that the integrity of this network remains largely intact at these variant enzymes.^87,141,143,160^

The transition state for hydride transfer to the specific phosphodianion substrate DHAP is strongly stabilized by interactions with the K120 and K204 side chains but these side chains interact only weakly with the transition state for hydride transfer to the nonspecific substrate GA (Figure 22). In other words, the substrate dianion binding energy is used to stabilize a protein conformation where the K120 and K204 side chain cations provide optimal transition state stabilization. The K120, K204, and D260 side chains are clustered around the DHAP carbonyl group (Figure 12). This favors rescue of lost activity for the K120A variant by protonation of the D260 side chain at this variant (Figure 23).

The N270A substitution causes a large increase in the second-order rate constant for unactivated *hl*GPDH-catalyzed hydride transfer to the truncated substrate GA and elimination of phosphite dianion activation of this hydride transfer reaction. These results are consistent with the notion that the dianion activation site for *hl*GPDH has evolved from a parent dehydrogenase catalyst of hydride transfer from NADH to a dianion truncated substrate (e.g., alcohol dehydrogenase) and that the conformation of this precursor catalyst is stabilized by the N270A substitution.

The important role for nonreacting fragments of the NAD^+^ cofactor in stabilization of the transition state for hydride transfer to the whole and truncated cofactors provides evidence that these fragments cooperate with the substrate phosphodianion and stabilize the active closed form (**E**_**C**_) of *hl*GPDH. The participation of the [292-LNGQKL-297] loop, whose side chains interact with both the substrate DHAP and the NADH cofactor, suggests that *hl*GPDH undergoes a global activating protein conformational change that is driven by interactions with both substrates.

## 5 Orotidine 5′-Monophosphate Decarboxylase (OMPDC)

### 5.1 Introduction

OMPDC catalyzes the final step in the biosynthesis of pyrimidine nucleosides.^169^ The 10^23^-fold rate acceleration for OMPDC-catalyzed decarboxylation of OMP to form UMP represents a large 31 kcal/mol stabilization of the transition state for formation of the very unstable vinyl carbanion reaction intermediate.^170,171^ This transition state stabilization is achieved through formation of an extensive network of protein side chain interactions shown in Figure 28 for the carbanion reaction intermediate analog 6′-hydroxyuridine phosphate (BMP) complexed to OMPDC from yeast (*Sc*OMPDC).^172^ By comparison, the value of *K*_m_ = 1.4 μM for OMP corresponds to a substrate binding energy of *ca* 8 kcal/mol, so that the interactions shown in Figure 28 provide only a modest stabilization of the Michaelis complex to OMP.

**Figure 28:**
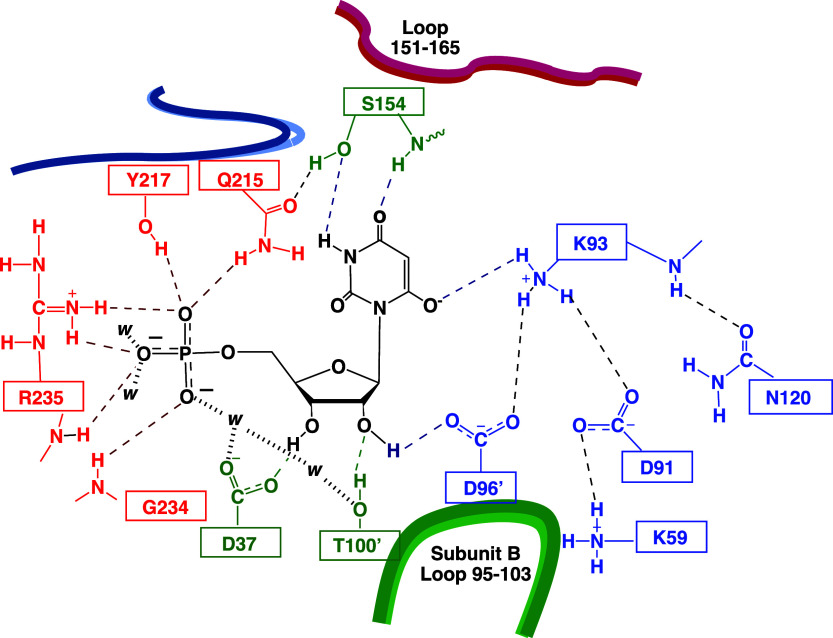
A pancake representation of *Sc*OMPDC complexed with an intermediate analog BMP (PDB 1DQX). Reproduced with permission from ref 88. Copyright 2021 American Chemical Society.

The variation in the protein structure for OMPDC from different organisms is greater than observed for TIM.^173^ However, the X-ray crystal structures for orthologs of OMPDC and bioinformatic analyses show that there is a strong conservation in the active-site side chains.^174–179^ Our work has focused mainly on the mechanism of action of OMPDC from yeast (*Sc*OMPDC) with additional experiments on the enzyme from *Methanobacter thermoautotrophicum* (*Mt*OMPDC). We consider it an open question of whether all of the important conclusions from this work hold for all orthologs of OMDPC.

OMPDC catalyzes the reactions illustrated in Scheme 15. (A) Decarboxylation of OMP and of 5-fluoroorotidine 5́-monophosphate (FOMP) to give, respectively, uridine 5́-monophosphate (UMP) and 5-fluorouridine 5́-monophosphate (FUMP) (Scheme 15A).^180^ (B) Direct and phosphite dianion activated decarboxylation of truncated substrates 1-(β-d-erythrofuranosyl)­orotate (EO) and 1-(β-d-erythrofuranosyl)-5-fluoroorotate (FO) to give, respectively, EU and FEU (Scheme 15B).^10,181^ (C) Exchange of the C-6 substrate hydrogen for deuterium from solvent D_2_O at UMP and FUMP to form, respectively, *d*-UMP and *d*-FUMP (Scheme 15C).^182,183^ (D) Direct and phosphite dianion activated exchange of the C-6 substrate hydrogen for deuterium from solvent D_2_O at 1-(β-d-erythrofuranosyl)-5-fluorouracil (FEU) to form *d*-FEU (Scheme 15D).^184^ (E) Direct, phosphite activated and sugar phosphate activated decarboxylation of 5-fluororotate to give 5-fluorouracil (Scheme 15E). This large suite of reactions has been examined in mutagenesis studies with the aim of localizing the site of stabilizing interactions between amino acid side chains and the different substrate fragments at the transition states for OMPDC-catalyzed decarboxylation and deuterium exchange reactions.^171^

**Scheme 15:**
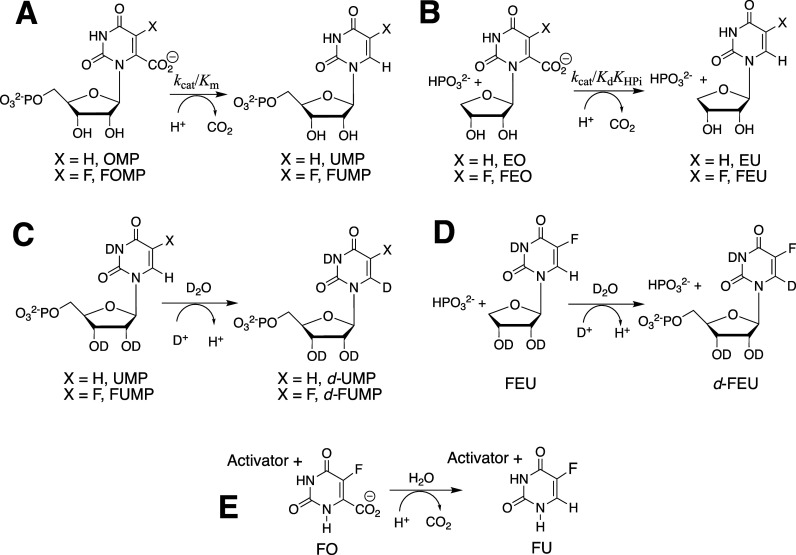
Decarboxylation and Deuterium-Exchange Reactions Catalyzed by OMPDC

### 5.2 Early Mutagenesis Studies

The cationic side chain of K93 is positioned to protonate the vinyl carbanion intermediate of direct decarboxylation of OMP or FOMP (Figure 28). An early study found that the K93C substitution at *Sc*OMPDC results in *a* > 10^8^-fold reduction in enzyme activity, and that part of this lost activity is recovered after reaction of the variant with bromoethylamine to give the (2-ethylammonium)­cysteine side chain.^185^ The protein-inhibitor interactions are otherwise heavily weighted toward stabilization of the Michaelis complex, but they provide an observed binding energy of only 8 kcal/mol for OMP, because the binding energy associated with the substrate binding interactions is utilized to drive an activating protein conformational change.^171^ For example, the Q215, Y217, and R235 side chains interact directly with the ligand phosphodianion, but the pyrimidine ring is *ca* 10 Å distant from these side chains (Figure 28). We were struck by the observation that the R235A substitution at *Sc*OMPDC results in an 800-fold increase in *K*_m_ and a 15-fold decrease in *k*_cat_ ^186,187^ because the side chain is not in a position to interact directly with the transition state for decarboxylation of enzyme-bound OMP (Figure 29). We have worked to show that this stabilization results from the utilization of binding interactions between the R235 side chain and OMP to hold OMPDC in the active closed conformation (Scheme 1A).

**Figure 29:**
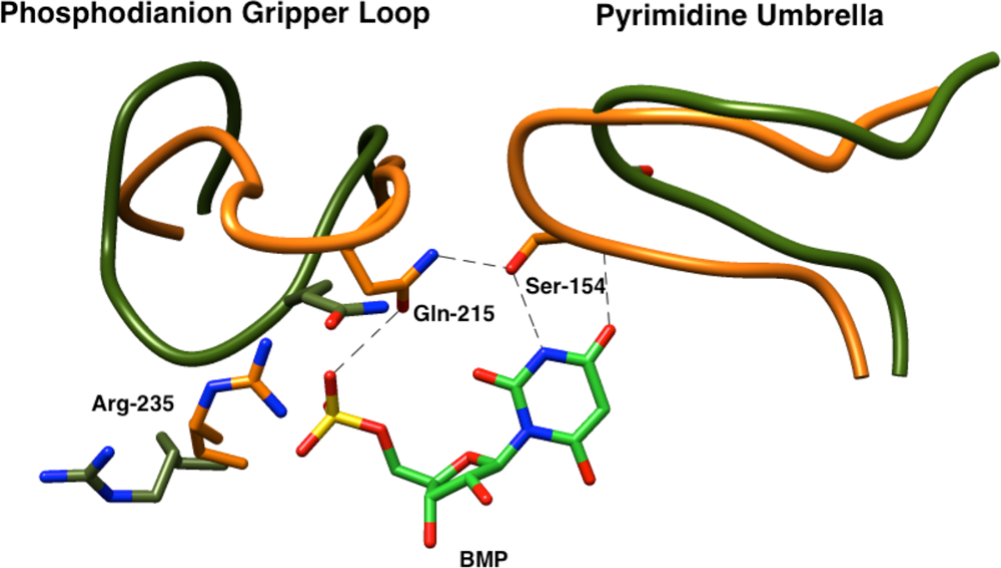
Superimposition of the partial X-ray crystal structures of the *Sc*OMPDC•BMP complex (orange, PDB 1DQX) and unliganded *Sc*OMPDC (green PDB 1DQW), which illustrates movement of the phosphodianion gripper loop (P202 to V220) toward the pyrimidine umbrella (A151 to T165) and formation of contacts between the gripper side chains R235 and Q215 and the substrate dianion. Reproduced with permission from ref 189. Copyright 2013 American Chemical Society.

### 5.3 The Activating Dianion-Driven Protein Conformational Change

Figure 29 presents a superposition of the structures of unliganded *Sc*OMPDC and the complex to BMP. Interactions between R235, Q215, and Y217 (not shown) side chains and the ligand dianion drive closure of the dianion gripper loop over the ligand dianion, while the hydrogen bond between the S154 and Q215 side chains moves an umbrella of loop side chains over the pyrimidine ring at the enzyme-bound ligand. Similar protein conformational changes are observed for OMPDC from other organisms,^173^ and it was concluded in an early crystallographic study on OMPDC from *E. coli* that “*without the phosphoryl group of the substrate the conformational changes are unlikely to occur at all and the enzyme stays in the open and inactive apo conformation*.”^188^ The authors were tempted “*to describe catalysis by OMPDC as an induced-fit mechanism*,” but failed to pursue this prescient proposal.

The R235A substitution at *Sc*OMPDC results in 19000-fold decreases in *k*_cat_/*K*_m_ for *Sc*OMPDC-catalyzed decarboxylation of OMP (Scheme 15A) and in (*k*_cat_/*K*_m_)_E•HPi_/*K*_d_ for phosphite dianion activated decarboxylation of the dianion-truncated substrate EO but has little effect on (*k*_cat_/*K*_d_)_E_ for unactivated *Sc*OMPDC-catalyzed decarboxylation of EO (Scheme 4 and Scheme 15B).^190^ This result shows that the transition states for *Sc*OMPDC-catalyzed decarboxylation of OMP and the substrate pieces EO + HP_i_ are stabilized by similar interactions with the R235 side chain, but that there are no significant stabilizing interactions between the R235 side chain and the orotate ring of EO. In other words, the interactions between R235 and the OMP phosphodianion or the phosphite dianion activator function exclusively to hold *Sc*OMPDC in the active closed conformation **E**_**C**_ (Scheme 1A,B).

More dramatic support for this conclusion was provided by the demonstration that the Q215A/Y217F/R235A triple substitution of dianion gripper side chains (Figure 28) at *Sc*OMPDC causes *k*_cat_/*K*_m_ = 1.1 × 10^7^ M^–1^ s^–1^ for wild-type *Sc*OMPDC to decrease to 0.037 M^–1^ s^–1^,^191^ but results in only a 9-fold decrease in *k*_cat_/*K*_m_ for decarboxylation of the phosphodianion-truncated substrate EO (Scheme 15B). This corresponds to very different 12 and 1.3 kcal/mol effects, respectively, of eliminating gripper side chains on the stability of transition states for decarboxylation of OMP and EO.^191^ The results show that *Sc*OMPDC is composed of a protein core that provides effective catalysis of the decarboxylation of EO and OMP and a dianion binding domain that uses dianion binding energy to drive a protein conformational change that activates the core for catalysis of decarboxylation at the orotate ring (Scheme 1).^173,190^

The results of an EVB computational study were reported to show that the binding of phosphate dianion to the Michaelis complex between *Mt*OMPDC and orotidine results in a *ca* 5 kcal/mol reduction in the activation barrier Δ*G* ^†^ for substrate decarboxylation due to a reduction in the reaction reorganization energy.^192^ It was claimed that these computational results model the experimental observation of phosphite dianion activation of OMPDC-catalyzed of EO (Scheme 15).^10,173^ We recommend that these calculations be reevaluated because they fail to model the observation that there is no detectable phosphate dianion activation of *Sc*OMPDC-catalyzed decarboxylation of orotidine due to severe steric effects on the rapid decarboxylation reactions of the EO and HP_i_ substrate pieces.^181,193^ These computational results also fail to provide a rationalization for effects discussed here of substitution of the Q215, Y217, and R235 dianion gripper side chains on the enzyme kinetic parameters.^194^

### 5.4 Effects of Substitutions on *K*_C_ for the Protein Conformational Change

The Q215, Y217, and R235 side chains interact directly with the phosphodianion of OMP to hold *Sc*OMPDC in the active closed protein conformation **E**_**C**_ (Scheme 1). The side chains are distant from the site of substrate decarboxylation, so that the effect of their substitution(s) on *k*_cat_/*K*_m_ for *Sc*OMPDC-catalyzed decarboxylation of OMP should reflect mainly the increase in *K*_m_ for formation of the Michaelis complex and with little change in the microscopic rate constant *k*_cat_ for decarboxylation of enzyme-bound substrate. In fact, as discussed briefly above, the side chain substitutions affect both (*k*_cat_)_obs_ (eq 6 and 7, Figure 30) and (*K*_m_)_obs_ (eq 6 and 8, Figure 30), where binding of OMP to wild-type ScOMPDC with *K*_d_ ≈ 1.0 mM is followed by a dianion-driven protein conformational change (*K*_C_ ≈ 1000, Figure 30A). The major effect of multiple dianion gripper side chain substitutions is to cause *K*_C_ ≈ 1000 for wild-type OMPDC to decrease to *K*_C_ ≪ 1.0 for severely crippled multiple variants (Figure 30B).

6 $$v=\frac{\left(\frac{k_{\mathrm{cat}}K_{c}}{1+K_{c}}\right)\left[\mathrm{OMP}\right]}{\left(\frac{K_{d}}{1+K_{c}}\right)+\left[\mathrm{OMP}\right]}$$

7 $$(k_{\mathrm{cat}})\mathrm{obs}=\left(\frac{k_{\mathrm{cat}}K_{c}}{1+K_{c}}\right)$$

8 $$(K_{m}{)}_{\mathrm{obs}}=\left(\frac{K_{d}}{1+K_{c}}\right)$$

**Figure 30:**
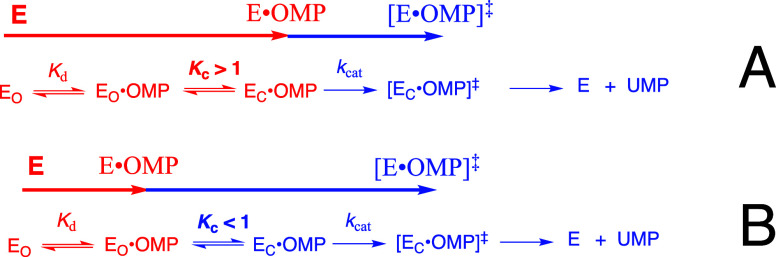
Representations of the kinetic mechanism for OMPDC-catalyzed decarboxylation that illustrate the effect of substitutions of gripper side chains on the equilibrium constant *K*_C_ for enzyme closure over the substrate. The red and blue lines show, respectively, the steps that control the values for the kinetic parameters (*K*_m_)_obs_ and (*k*_cat_)_obs_. (A) Decarboxylation reaction catalyzed by wild-type *Sc*OMPDC where the Michaelis complex exists mainly in the closed conformation E_C_•OMP (*K*_C_ ≈ 1000). (B) Decarboxylation reaction catalyzed by variants of *Sc*OMPDC where the Michaelis complex exists mainly in the open conformation E_O_•OMP (*K*_C_ < 1).

The effects of single and multiple side chain substitutions at wild-type *Sc*OMPDC on the kinetic parameters (*K*_m_)_obs_ and (*k*_cat_)_obs_ conform to eq 6–8 derived for Figure 30.^191^ An important lesson from this work is that substitutions of remote gripper side chains affect the macroscopic kinetic parameter (*k*_cat_)_obs_ (eq 6 and 7), without changing the microscopic rate constant *k*_cat_ (Figure 30) for decarboxylation of **E**_**C**_ **•OMP**, when the barrier to (*k*_cat_)_obs_ includes the barrier for conversion of the dominant Michaelis complex **E**_**O**_ **•OMP** to **E**_**C**_ **•OMP** (*K*_C_ < 1, Figure 30B).

(1) The single Q215A and Y217F substitutions at wild-type *Sc*OMPDC result in (60–80)-fold increases in (*K*_m_)_obs_ for decarboxylation of OMP and small (<2-fold) increases in (*k*_cat_)_obs_.^191^ These changes are consistent with *K*_C_ ≈ 1000 for the wild-type OMPDC. The small increases in (*k*_cat_)_obs_ are due to increases in the rate constant for product release from the variant enzymes, which limits the values for (*k*_cat_)_obs_. The Q215A/Y217F double substitution causes (*K*_m_)_obs_ (eq 6 and 8) to decrease to the limiting value of 1 mM and a 5-fold decrease in (*k*_cat_)_obs_. There is now a decrease in (*k*_cat_)_obs_ (eq 6 and 7) because the Michaelis complex to the double variant exists mainly as E_O_•OMP and only ≈ 20% of the total Michaelis complex is present in the active E_C_•OMP form at saturating OMP.^191^

(2) The R235A substitution at wild-type *Sc*OMPDC destabilizes the rate-determining transition state for *Sc*OMPDC-catalyzed decarboxylation by 5.6 kcal/mol; 4.0 kcal/mol of this effect is expressed as a 700-fold increase in (*K*_m_)_obs_ to a limiting value of (*K*_m_)_obs_ ≈ *K*_d_ = 1 mM. The remaining effect of the mutation (1.6 kcal/mol) is the 15-fold decrease in (*k*_cat_)_obs_.

(3) The R235A substitution at the Q215A variant likewise results in a large 5.8 kcal/mol destabilization of the rate-determining transition state for Q215A variant-catalyzed decarboxylation of OMP. However, now this substitution causes only a 13-fold increase (*K*_m_)_obs_ from 0.11 to 1.4 mM and a large 1200-fold decrease in (*k*_cat_)_obs_ from 24 to 0.020 s^–1^, because the barrier to *K*_C_ (Figure 30B) is now included in the activation barrier to (*k*_cat_)_obs_

### 5.5 Amino Acid Variant Cycles

#### 5.5.1 Two Amino Acid Variant Cycles

The effect of sequential elimination of S154 and Q215 side chains on the activation barrier Δ*G*_OMP_^†^ for *k*_cat_/*K*_m_ for *Sc*OMPDC-catalyzed decarboxylation is summarized in Figure 31A.^195^ The effect depends upon the order of the substitutions, with the first Q215A or S154A substitutions resulting, respectively, in 2.2 and 5.8 kcal/mol increases in Δ*G*_OMP_^†^, while the same substitutions at single Q215A or S154A variants cause smaller 0.3 or 3.9 kcal/mol increases in Δ*G*_OMP_^†^.^195^ The difference in the effect of the first and second substitutions represents the 2 kcal/mol stabilizing interaction of Q215 with the substrate phosphodianion (Figure 29). This interaction is eliminated at both the Q215A and S154A variants because S154 holds Q215 close to the dianion so that there is no dianion interaction at the S154A variant (Figure 29). The 3.9 kcal/mol effect of the S154A substitution at the Q215A variant provides the isolated S154 side chain interaction. Similar effects are predicted for an amino acid substitution at wild-type OMPDC and for the same substitution at a single variant, when there are no interactions between the two substituted side chains.^196,197^ This is illustrated by the cycle from Figure 31B for Q215A or Y217F substitutions at wild-type OMPDC and at Y217F or Q215A variants.^191^

**Figure 31:**
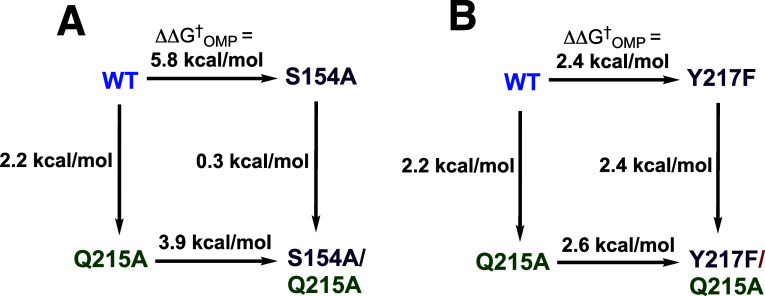
Variant cycles that illustrate the effect (ΔΔ*G*_OMP_^†^) of consecutive substitutions of two amino acid side chains on the activation barrier Δ*G*_OMP_^†^ for OMPDC-catalyzed decarboxylation of OMP. The values of ΔΔ*G*_OMP_^†^ were calculated from the ratio of the values of *k*_cat_/*K*_m_ for parent and variant enzyme-catalyzed reactions.

#### 5.5.2 Three Amino Acid Variant Cycles

Interactions of OMP with the S154, Q215, Y217, and R235 side chains from *Sc*OMPDC activate the enzyme for catalysis by stabilizing the closed dianion-gripper loop and pyrimidine umbrella scaffold (Figure 29). The role of these structural elements in catalysis was examined by determining the effect of their systematic deconstruction on enzyme activity. This was accomplished by preparing the full set of 16 single, double, triple, and quadruple variants of the S154A, Q215A, Y217F, and R235A side chains of *Sc*OMPDC,^191,198^ after noting that the Y217F substitution is more conservative than Y217A (section 5.10.).^186,198^

The effects of all single Q215A, Y217F, and R235A substitutions at wild-type and variant forms of *Sc*OMPDC on Δ*G*_OMP_^†^ (ΔΔ*G*_OMP_^†^) for decarboxylation of OMP are summarized in Figure 32A, where ΔΔ*G*_OMP_^†^ is calculated from the ratio of the values of *k*_cat_/*K*_m_ for the respective parent and variant enzyme-catalyzed reactions. Figure 32B presents the effects of single substitutions of the same three side chains on Δ*G*_OMP_^†^ but now starting with the S154A variant. The effects of these substitutions on enzyme activity may also be illustrated on a single four-dimensional hypercube,^199^ but we have not been able to use this hypercube to provide additional mechanistic insight.

**Figure 32:**
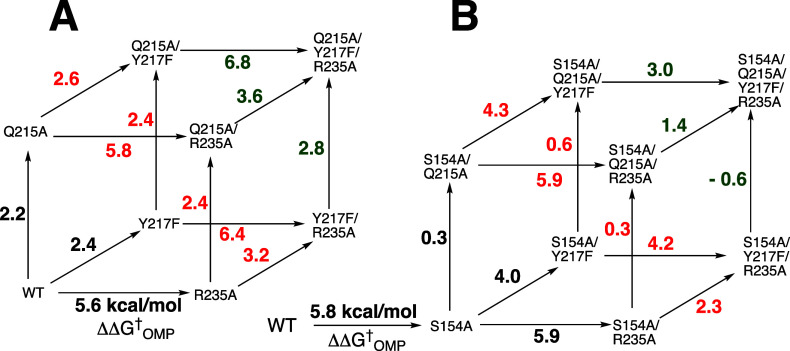
Cubes that show the effect (ΔΔ*G*_OMP_^†^) of consecutive Q215A, Y217F, and R235A substitutions on the activation barrier Δ*G*_OMP_^†^ for *Sc*OMPDC-catalyzed decarboxylation of OMP. The values of ΔΔ*G*_OMP_^†^ were calculated from the ratio of the values of *k*_cat_/*K*_m_ for parent and variant enzyme-catalyzed reactions. (A) The effects on Δ*G*_OMP_^†^ starting with wild-type *Sc*OMPDC. (B) The effects on Δ*G*_OMP_^†^ starting with the S154A variant. Key: Black values, effects on Δ*G*_OMP_^†^ for the parent enzyme. Red values, effect on Δ*G*_OMP_^†^ for single variants. Green values, effect on Δ*G*_OMP_^†^ for double variants. Reproduced with permission from ref 198. Copyright 2018 American Chemical Society.

Parts A and B of Figure 32 show that the effect of single side chain substitutions at OMPDC on Δ*G*_OMP_^†^ depends upon the side chains that remain from the original four amino acid network. For example, values of 5.6, 6.8, and 3.0 kcal/mol, respectively, were determined for ΔΔ*G*_OMP_^†^ for R235A substitutions at wild-type, Q215A/Y217F and S154A/Q215A/Y217F variants of *Sc*OMPDC. There are several questions to consider in developing an explanation for the context dependence of these side chain substitutions.

(1) What is the intrinsic stabilization of the transition state for the wild-type *Sc*OMPDC-catalyzed reaction by interactions with single amino acid side chains? There are no direct gripper side chain interactions with the orotate ring at the decarboxylation reaction transition state (Figure 29). Figure 32A therefore shows the effects of side chain substitutions on the stability of active loop-closed OMPDC (**E**_**C**_) relative to the inactive open enzyme **E**_**O**_ (*K*_C_, Figure 30). The sum of the effects of Q215A (2.2 kcal/mol), Y217F (2.4 kcal/mol), and R235A (5.6 kcal/mol) substitutions on Δ*G*_OMP_^†^ (11.2 kcal/mol) is similar to the 11.7 kcal/mol difference in the activation barriers for OMPDC-catalyzed reactions of the whole substrate OMP and the phosphodianion truncated substrate EO. The effect of single Q215A, Y217F, and R235A substitutions account for the total substrate dianion activation and therefore represent the intrinsic contributions of each side chain to this activation.

(2) Under what conditions is the effect of a single side chain substitution on enzyme activity greater than the intrinsic side chain interaction with the substrate dianion because the substitution perturbs the interaction of other network side chains (see Figures 13, 16A, 17, 18, and 20)? The effects on Δ*G*_OMP_^†^ of Q215A (2.8 compared to 2.2 kcal/mol), Y217F (3.6 compared to 2.4 kcal/mol), and R235A (6.8 compared to 5.6 kcal/mol) substitutions at double variants missing the other two gripper side chains are significantly larger than the effect of the same substitution on Δ*G*_OMP_^†^ for wild-type *Sc*OMPDC (Figure 32A). This shows that substitution of two out of three gripper side chains results in an apparent tightening of the interaction of the remaining side chain at loop-closed *Sc*OMPDC. We propose that the first two substitutions erode the stability of the side chain network that holds the enzyme in the closed conformation, and that the third substitution now results in a *ca* 1.0 kcal/mol decrease in the 4 kcal/mol interaction between S154 and the decarboxylation transition state (Figure 31A) which is added to observed effect of the side chain substitution on Δ*G*_OMP_^†^

(3) Under what conditions is the effect of a single side chain substitution on Δ*G*_OMP_^†^ smaller than the intrinsic side chain interaction with the substrate dianion? We note for Figure 32B that the effects on Δ*G*_OMP_^†^ of Q215A (−0.6 kcal/mol), Y217F (1.4 kcal/mol), and R235A (3.0 kcal/mol) substitutions at variants, where these are the only remaining side chains from the original network, are much smaller than the effect of the same substitution on Δ*G*_OMP_^†^ for the reaction catalyzed by wild-type OMPDC. This is consistent with an acute erosion of side chain function at severely crippled forms of *Sc*OMPDC, so that the side chain-dianion interactions now provide minimal stabilization of the active closed (**E**_**C**_) conformation of OMPDC.

The results from Figure 32 are consistent with a model where (i) The effect of single amino acid substitutions on Δ*G*_OMP_^†^ for the reaction catalyzed by wild-type OMPDC is close to the intrinsic side chain interaction with the substrate dianion, because interactions of the remaining side chains that stabilize the stiff active-site cage are not perturbed by the first substitution. (ii) The substitution of any two dianion gripper side chains relax the protein cage, so that substitution of the last gripper side chain has the effect of weakening transition state stabilization by the interaction between S154 and the pyrimidine ring (Figure 29). (iii) The deletion of any two gripper side chains plus S154 distorts the cage structure and reduces stabilization of the active-site cage by the final gripper side chain.

### 5.6 Ground-State Destabilization

A computational study to model decarboxylation of OMP catalyzed by the enzyme from *Mt*OMPDC provided evidence that stabilizing interactions between the protein and substrate phosphodianion introduce destabilizing electrostatic interactions between the protein and OMP carboxylates that are relieved at the decarboxylation transition state.^25^ Stabilizing protein–dianion interactions would normally be expressed as a decrease in *K*_m_ for formation of an unstressed Michaelis complex E•OMP_U_. However, if the stabilizing side chain interactions are used to generate a balancing set of destabilizing electrostatic interactions at the Michaelis complex (E•OMP_st_) which are then relieved at the unstressed decarboxylation transition state [E•OMP_U_]^**†**^, then the stress-inducing binding interactions will cause offsetting increases in *K*_m_ and in *k*_cat_ for *Mt*OMPDC-catalyzed decarboxylation but will not affect the barrier to *k*_cat_/*K*_m_ for reaction of the unstressed substrate to form the unstressed transition state (Figure 33).

**Figure 33:**
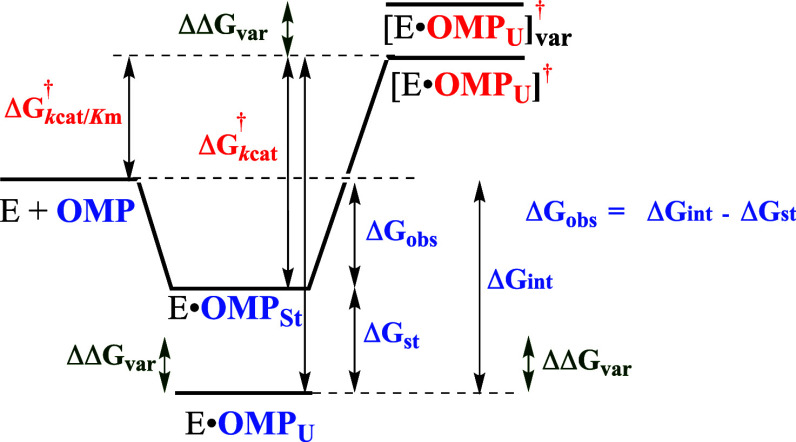
Comparison of the hypothetical activation barriers for OMPDC-catalyzed decarboxylation of an unstressed substrate E•OMP_U_ and the stressed substrate E•OMP_St_, where the stress (Δ*G*_st_) is introduced by using stabilizing protein phosphodianion interactions to create destabilizing electrostatic interactions between protein carboxylate side chains and the substrate carboxylate that are relieved at the decarboxylation transition state. A side chain substitution that reduces the total substrate binding energy by (ΔΔ*G*_var_) by specifically reducing the binding energy utilized to introduce electrostatic stress (ΔΔ*G*_st_) will increase the overall reaction barrier Δ*G*_kcat/Km_^†^ by ΔΔ*G*_var_ without affecting the observed binding energy Δ*G*_obs_.

Substitutions of dianion gripper side chains that specifically reduce ground state stress at enzyme-bound OMP (ΔΔ*G*_var._), Figure 33) will result in a similar reduction in the intrinsic substrate binding energy Δ*G*_int_, but will not affect Δ*G*_obs_ or *K*_m_. The smaller effect of stress relieved at the decarboxylation transition state would then result in an increase in the activation barrier for formation of the unstressed decarboxylation transition state [E•OMP_U_]^†^ and a decrease in *k*_cat_. The net effect of these substitutions would then be to cause equivalent decreases in *k*_cat_ and *k*_cat_/*K*_m_, but no change in *K*_m_. By contrast, the observation that single Q215A, Y217F, and Q215A/Y217F substitutions of dianion gripper side chains at *Sc*OMPDC result in only small [≤3-fold] changes in *k*_cat_ and that nearly their entire effect is expressed as increases in *K*_m_ shows that interactions of enzyme-bound OMP with these side chains function mainly to stabilize the Michaelis complex for *Sc*OMPDC and do not introduce electrostatic stress at this complex.^191^

### 5.7 A Backbone Amide to Ester Substitution

The conserved S127 side chain at *Mt*OMPDC plays a role similar to S154 at *Sc*OMPDC in stabilizing the decarboxylation transition state by formation of the hydrogen bond between the backbone amide of S127 and O-4 of the substrate pyrimidine. It was proposed that this interaction provides a strong stabilization of a carbene-like transition state.^200^ The contribution of this hydrogen bond to transition state stabilization was examined by determining the effect of replacing an ester linkage for the S127 backbone amide by substitution of l-glycerate for S127 for *Mt*OMPDC (Figure 34).^201^ This substitution results in a 2.6 kcal/mol increase in the activation barrier to *k*_cat_/*K*_m_ for decarboxylation of FOMP. This was attributed to the loss of a hydrogen bond that stabilizes the reaction transition state, with the caveat that the observed effect on the activation barrier may also include a destabilizing interaction between the protein ester oxygen and the decarboxylation reaction transition state.^201^

**Figure 34:**
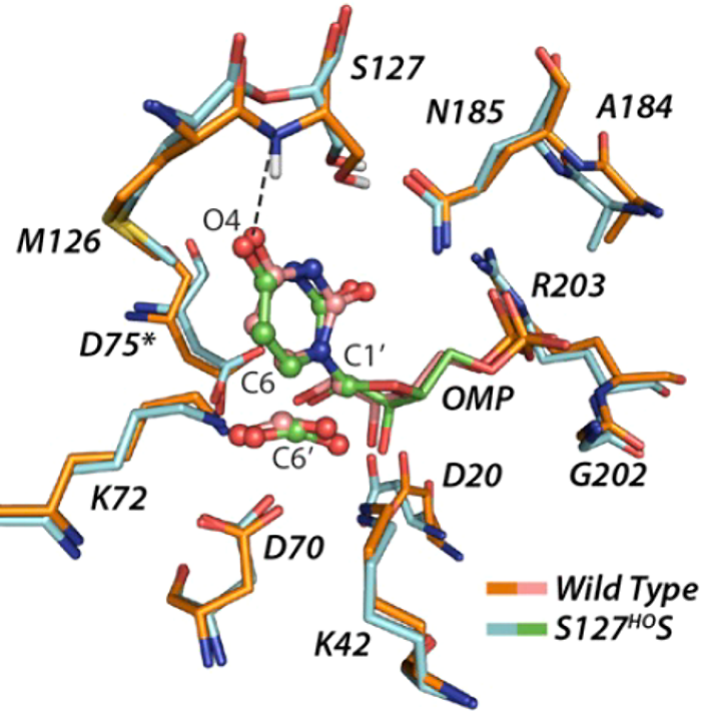
Representative structures from molecular dynamic simulations of the transition states for the decarboxylation of OMP catalyzed by wild-type *Mt*OMPDC and the variant enzyme with l-glycerate substituted for l-serine at position 127. Reproduced with permission from ref 201. Copyright 2014 U.S. National Academy of Sciences.

### 5.8 Side Chain Effects on the Product Deuterium Isotope Effect

The *Sc*OMPDC-catalyzed decarboxylation of OMP or FOMP in 50/50 (v/v) HOH/DOD gives a 1:1 yield of *h*-UMP and *d*-UMP or of *h*-FUMP and *d*-FUMP, consistent with unitary (*k*_H_/*k*_D_ = 1.0) primary product deuterium isotope effects (1°PDIEs).^202^ Unitary 1° PDIEs were also determined for *Sc*OMPDC-catalyzed decarboxylation of OMP and of FOMP catalyzed by several variant enzymes.^203^ The observation that there is no discrimination between the reaction of hydrogen and deuterium at the product-determining transition step (protonation of the vinyl carbanion intermediate) requires that the reaction intermediate be protonated by the initial interacting hydron from the K93 side chain cation and at a much faster rate than rotation about the side chain −C-ND_2_H^+^ bond that exchanges the positions of −H and −D (*k*_p_ ≫ *k*_rot_ Figure 35A). This rotational exchange of hydrons is required for selection of the more reactive −H for transfer to product that would result in a normal 1°PDIE.^202^

**Figure 35:**
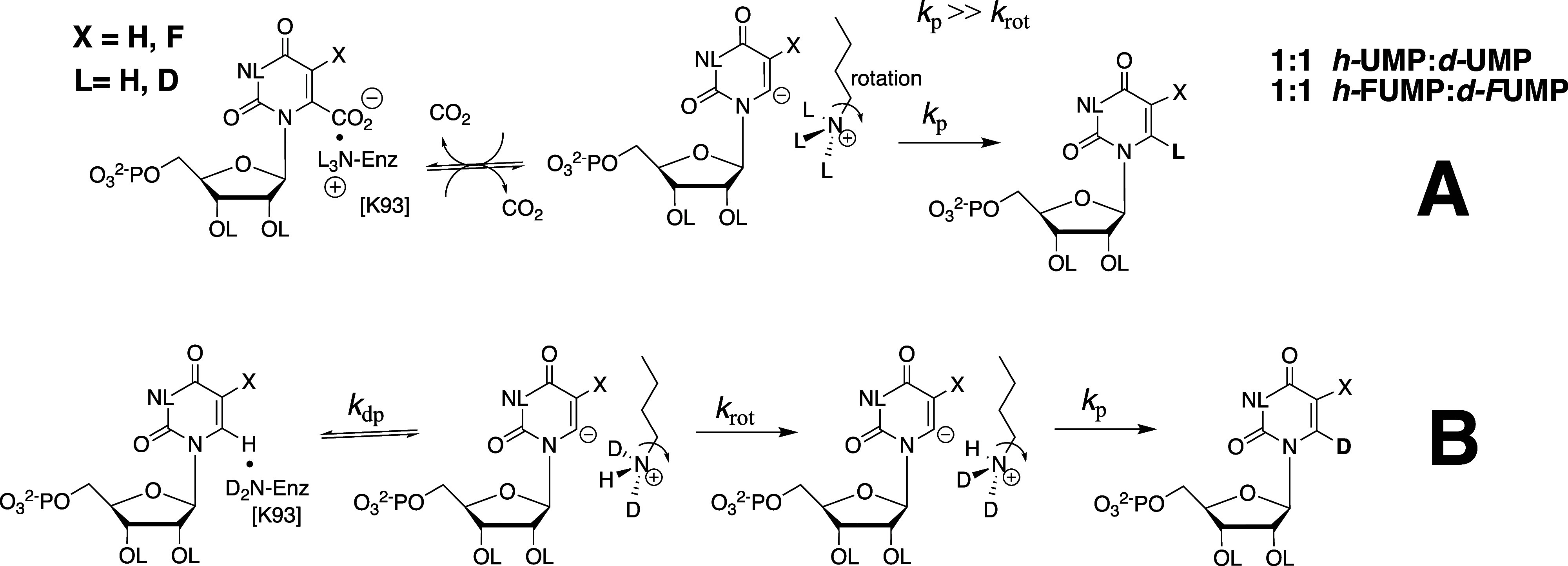
(A) The *Sc*OMPDC-catalyzed decarboxylation of OMP or FOMP in 50/50 (HOH/DOD) to form a 1:1 yield of hydrogen and deuterium labeled products. (B) The *Sc*OMPDC-catalyzed D-exchange reactions of UMP or FUMP in 100% DOD. The reactions illustrated by (A) and (B) both proceed through vinyl carbanion intermediates, but rotation (*k*_rot_) about the −CND_2_H^+^ bond of the cationic K93 side chain, that moves the solvent −D into a position to protonate the vinyl carbanion intermediate, is only required to observe the deuterium exchange reaction.

### 5.9 *Sc*OMPDC-Catalyzed Deuterium-Exchange Reactions

*Sc*OMPDC catalyzes the deuterium-exchange reactions in D_2_O at whole substrates UMP and FUMP, and at the phosphodianion truncated substrate FEU (Scheme 15C,D) by the mechanism illustrated in Figure 35B for XUMP.^182–184^ The activation barriers to *Sc*OMPDC-catalyzed decarboxylation (Figure 35A) and D-exchange reactions (Figure 35B) are related to the stability of the vinyl carbanion intermediate common to both reactions. The large ratio 1 × 10^7^ M^–1^ s^–1^/1.2 × 10^–5^ M^–1^ s^–1^ = 8 × 10^11^ for the second-order rate constants *k*_cat_/*K*_m_ for OMPDC-catalyzed decarboxylation of OMP and for D-exchange of UMP corresponds to a 16 kcal/mol difference in reaction activation barriers. This is similar to the estimated 17 kcal/mol difference in enzymatic stabilization of the rate-determining transition states for OMPDC-catalyzed decarboxylation (31 kcal/mol)^170^ and D-exchange (14 kcal/mol).^182^

These differences are partly or entirely due to (1) The *ca* 6 kcal/mol larger barrier to *k*_rot_ for rotation about the C-ND_2_H^+^ bond of the cationic K93 side chain that exchanges reactant −H for solvent −D (Figure 35B) than for *k*_p_ for direct protonation of the carbanion intermediate (Figure 35A).^202,203^ (2) The *ca* 10 kcal/mol stabilization of the late, product-like, transition state for the decarboxylation reaction by interactions of hydrophobic side chains with the nascent product CO_2_.^182^ These include F89, I183, and I230 at a hydrophobic pocket at *Sc*OMPDC that is on the opposite face of the pyrimidine ring from K93,^175,182,204^ and the I96, L123, and V155 side chains at a pocket close to the enzyme-bound OMP carboxylate at *Mt*OMPDC.^205,206^ The role for the hydrophobic side chains at *Sc*OMPDC has not been investigated by site-directed mutagenesis, but results from studies on *Mt*OMPDC show that substitutions of I96, L123, and V155 side chains by neutral hydrophilic residues result in up to a 400-fold decrease in *k*_cat_ for decarboxylation of OMP, but with minimal effect on *k*_cat_ for deuterium exchange into FUMP.^206^ This provides direct evidence that these hydrophobic side chains function to stabilize nascent CO_2_ product at the late transition state for decarboxylation of OMP.

The effect of substitutions of dianion gripper side chains (Figure 28) on the activation barriers for *Sc*OMPDC-catalyzed decarboxylation of OMP and the exchange of the C-6 hydrogen of FUMP for deuterium from D_2_O are compared in Table 3.^189,191,204^ The critical observation is that these substitutions cause a larger destabilization of the transition state for the D-exchange compared to the decarboxylation reaction. This is consistent with larger intrinsic stabilizing side chain interactions,^204^ but the observation that *Sc*OMPDC provides a 16 kcal/mol smaller total stabilization of the D-exchange transition state (see above)^182^ suggests that the individual Q215, Y217, and R235 side chains are unlikely to show larger *intrinsic* interactions with the D-exchange transition state.

**Table 3:** Effect of Side Chain Substitutions on the Activation Barriers for *Sc*OMPDC-Catalyzed Decarboxylation of OMP (Δ*G*_OMP_^†^) and for Exchange of the C-6 Hydrogen of FUMP for Deuterium in D_2_O (Δ*G*_FUMP_^†^)

enzyme	ΔΔ*G*_OMP_^†^ (kcal/mol)^a^	ΔΔ*G*_FUMP_^†^ (kcal/mol)^b^	[ΔΔ*G*_FUMP_^†^ – ΔΔ*G*_OMP_^†^] (kcal/mol)
Q215A	2.2	3.5^c^	1.3
Y217F	2.4	4.1^c^	1.7
Q215A/Y217F	4.8	6.8^c^	2.0
R235A	5.6	7.2^d^	1.6

a Effect of the amino acid substitution(s) on the activation barrier Δ*G* ^†^ for *k*_cat_/*K*_m_ for *Sc*OMPDC-catalyzed decarboxylation of OMP.^191,198^

b Effect of the amino acid substitution(s) on *k*_cat_/*K*_d_ for the deuterium exchange reaction of FUMP.

c Reference 204.

d Reference 189.

The surprising difference in the effects of single gripper side chain substitutions on Δ*G*_OMP_^†^ and Δ*G*_FUMP_^†^ (Table 3) is consistent with a greater weakening in the interactions of the remaining dianion-gripper side chains at the transition state for the D-exchange compared to the decarboxylation reaction, where the effect of the first side chain substitution on Δ*G*_OMP_^‡^ is close to the intrinsic side chain interaction with the substrate dianion (section 5.5.2). The decarboxylation reaction transition state is stabilized by a *ca*. 10 kcal/mol interaction with the nascent CO_2_ that is absent from the transition state for the D-exchange reaction.^204^ The substitution of single gripper side chains does not cause a significant weakening of interactions between the remaining side chains and the bound ligand at the decarboxylation reaction transition state, in part because the protein is locked into a tight structure by the large 10 kcal/mol interaction with the CO_2_ product. We propose that, by comparison, the loss of strong protein interactions with CO_2_ at the transition state for the D-exchange reaction has the effect of relaxing the interactions of the dianion gripper and pyrimidine umbrella side chains, so that single substitutions of these side chains are now accompanied by a weakening in the stabilizing interactions of the remaining side chains.

### 5.10 Effect of Side Chain Substitutions on the Protein Conformational Change

There have been few enzyme kinetic studies to probe the effect of site-directed amino acid substitutions on protein conformational changes, because conformational changes are seldom strongly rate-determining for enzyme turnover.^35,207^ In the case of *Sc*OMPDC-catalyzed decarboxylation, the binding of OMP is partly rate determining for enzyme turnover at low [OMP] and the release of product UMP is partly rate determining for turnover at high [OMP].^208^ The 5-F at 5-fluoroorotate and FOMP provides strong stabilization, respectively, of the vinyl carbanion-like transition states for nonenzymatic and enzyme-catalyzed decarboxylation reactions but does not otherwise affect the rate constants *k*_c_ and *k*_‑c_^′^ for the protein conformational change (Scheme 16). Consequently, these rate constants are strongly rate-determining for wild-type *Sc*OMPDC-catalyzed decarboxylation of FOMP and control the observed effect of side chain substitutions on the enzyme kinetic parameters.^182^

**Scheme 16:**
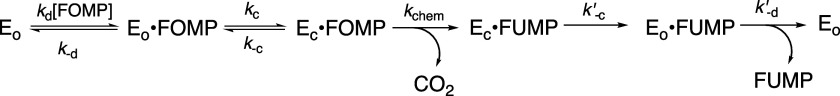
*Sc*OMPDC-Catalyzed Decarboxylation of FOMP that Includes Kinetically Significant Protein Conformational Changes Required for Substrate Binding (*k*_c_) and Product Release (*k*_‑c_^′^)

The 5-F at the crippled substrate FEO causes a 400-fold increase in *k*_cat_/*K*_m_ for *Sc*OMPDC-catalyzed decarboxylation of EO for reactions that are limited by the chemical decarboxylation step.^181^ By comparison, similar values of *k*_cat_/*K*_m_ = 1.0 × 10^7^ M^–1^ s^–1^ and 1.2 × 10^7^ M^–1^ s^–1^ are observed, respectively, for *Sc*OMPDC-catalyzed decarboxylation of OMP and FOMP.^181^ Both values of *k*_cat_/*K*_m_ are smaller than *k*_d_ ≈ 1 × 10^8^ M^–1^ s^–1^ determined for fully diffusion-controlled enzyme-catalyzed reactions.^36,209,210^ The rate constant *k*_c_ for the protein conformational change that traps substrate at the enzyme (Scheme 16) is therefore rate determining for wild-type *Sc*OMPDC-catalyzed decarboxylation of FOMP. The C-5 F causes a 6-fold increase in *k*_cat_ from 16 s^–1^ to 95 s^–1^ for decarboxylation of OMP compared to FOMP, compared to the 400-fold increase in *k*_cat_ expected for the fluorine substituent effect when the chemical step (*k*_chem_) is fully rate determining. The observed *k*_cat_ = 95 s^–1^ is equal to *k*_–c_^′^ for the conformational change that opens the protein cage and allows release of FUMP (Scheme 16, *k*_chem_ ≫ *k*_cat_ = *k*_–c_^′^ = 95 s^–1^).^181,208^ These conclusions are supported by the results of a study on the effect of changing solvent viscosity on the kinetic parameters for *Sc*OMPDC-catalyzed decarboxylation of OMP and FOMP.^211^

Substitutions of Q215, Y217, or R235 side chains cause very different changes in *k*_cat_/*K*_m_ or *k*_cat_ for *Sc*OMPDC-catalyzed decarboxylation of OMP and FOMP when the rate-determining steps for decarboxylation of the two substrates are different. When the rate of decarboxylation of OMP is limited by the chemical decarboxylation step (*k*_chem_), the side chain substitutions cause *k*_cat_/*K*_m_ to decrease to (*k*_cat_/*K*_m_)_H_ = 0.036 M^–1^ s^–1^ for the Q215A/Y217F/R235A variant. By comparison, the first Q215A or Y217F substitutions at wild-type *Sc*OMPDC cause *k*_cat_/*K*_m_ for decarboxylation of FOMP to decrease to (*k*_cat_/*K*_m_)_F_ ≈ 1 × 10^6^ M^–1^ s^–1^ due to a decrease in *k*_c_, which is sensitive to the total number of gripper side chains that hold the enzyme in the closed conformation (Scheme 16). Further substitutions cause a change to rate determining chemical decarboxylation, and a decrease to (*k*_cat_/*K*_m_)_F_ = 28 M^–1^ s^–1^ for decarboxylation catalyzed by the Q215A/Y217F/R235A variant. The large ratio (*k*_cat_/*K*_m_)_F_/(*k*_cat_/*K*_m_)_H_ = 780 for the triple variant is as expected for decarboxylation reactions through a transition state that is strongly stabilized by the C-5 fluorine.^182^

Comparisons of the effect of substitutions of Q215, Y217, and R235 side chains on the kinetic parameters for *Sc*OMPDC-catalyzed decarboxylation of OMP and FOMP provide additional insight into the effect of changing gripper loop structure on the rate constants for rate-determining protein conformational changes (Scheme 16).^212^ The difference in the effects of Y217F and Y217A substitutions is particularly revealing.^212^

(1) The Y217F substitution at *Sc*OMPDC causes *k*_cat_ for decarboxylation of FOMP to *increase* from 95 s^–1^ to 430 s^–1^ due to a 4.5-fold increase in *k*_–c_^′^ for rate-determining loop opening. By comparison, there is a 10-fold decrease in *k*_cat_/*K*_m_ due to the 10-fold falloff in *k*_c_ for rate determining loop closure (Scheme 16).^212^ These changes in *k*_–c_^′^ and *k*_c_ are due to the loss of the side chain phenol hydroxyl that interacts with the OMP phosphodianion and holds OMPDC in the closed conformation. The decrease in the stability of active **E**_**C**_ **•FOMP** relative to inactive **E**_**O**_ **•FOMP** for the Y217F variant (see Figure 30 for **E**_**C**_ **•OMP** and **E**_**O**_ **•OMP**) results in an 80-fold increase in *K*_m_ for variant-catalyzed decarboxylation of FOMP compared with the wild-type enzyme-catalyzed reaction.^191^

(2) By comparison the Y217A substitution at *Sc*OMPDC causes a 12-fold decrease in *k*_cat_ for decarboxylation of FOMP from 95 to 8.2 s^–1^ due to a large decrease in *k*_‑c_^’^ for rate determining loop opening. There is an even larger 900-fold decrease to *k*_cat_/*K*_m_ = 1.3 × 10^4^ M^–1^ s^–1^ due to a decrease *k*_c_ for rate determining loop closure (Scheme 16). The Y217A substitution is estimated to cause a large decrease in *K*_C_ = 1000 for the conformational change at the wild-type enzyme (Figure 36) to *K*_C_ = 0.45. The large destabilization of the closed form of *Sc*OMPDC by the Y217A substitution is expressed as the large barrier for formation of the transition state for the protein conformational change of the variant enzyme, whose rate limits the values of *k*_cat_ (*k*_‑c_^’^ rate determining) and *k*_cat_/*K*_m_ (*k*_c_ rate determining) for Y217A variant-catalyzed decarboxylation of FOMP.^212^

**Figure 36:**
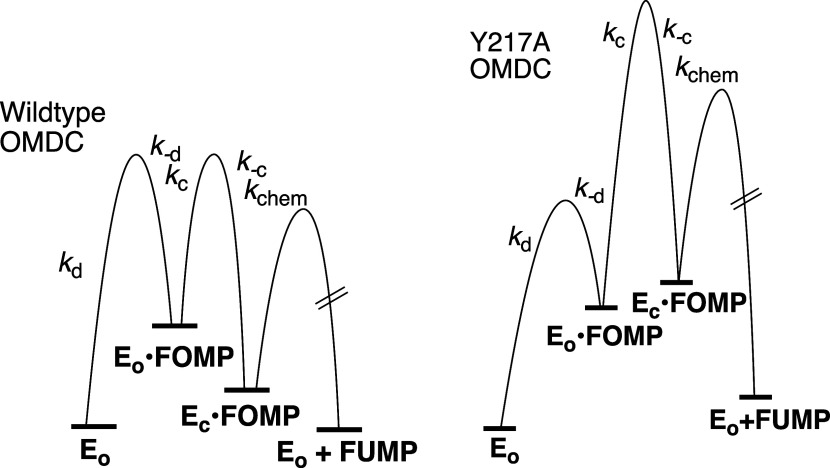
Free energy profiles for wild-type and Y217A variant *Sc*OMPDC-catalyzed decarboxylation of FOMP (Scheme 16) that are drawn for reactions at [S] ≪ *K*_m_ and using the estimated rate and equilibrium constants reported in table 2 of ref 212.

The kinetic parameters for decarboxylation of FOMP catalyzed by wild-type and the Y217A variant *Sc*OMPDC were used to calculate the rate and equilibrium constants reported in Table 2 of ref 212 and to construct the reaction free energy profiles shown in Figure 36 for Scheme 16.^212^ These and other data provide support for the proposal that the structure for the large [P202–V220] phosphodianion gripper loop has evolved to optimize both the dianion binding energy and to ensure the loop flexibility required to undergo the relatively fast and kinetically competent opening and closure over enzyme-bound OMP.

### 5.11 Protein-Ribosyl Hydroxyl Interactions

There is a 4.6 kcal/mol larger activation barrier for *Sc*OMPDC-catalyzed decarboxylation of 2′-deoxyOMP compared to OMP.^213^ This is nearly 50% of the estimated total 10.6 kcal/mol stabilization of the decarboxylation transition state by interactions with the ribosyl ring.^214^ Three side chains contribute to these interactions, D37, D96′ and T100′, where the side chains designated as prime are located at the smaller portion of the enzyme active site that is contributed by the second protein subunit. The D96′ side chain is part of the network of charged residues discussed below (K59, D91, K93, and D96′) that interact with the substrate pyrimidine ring (see section 5.12).

#### 5.11.1 Side Chain Effects on Dimer Stability

The D37G and T100’A substitutions at *Sc*OMPDC result in only small 0.5 kcal/mol increases in Δ*G*_FOMP_^†^ for decarboxylation of FOMP because of their small effect on the barrier to *k*_c_ (Scheme 16) for rate determining closure of *Sc*OMPDC over the enzyme-bound substrate.^215^ The same substitutions cause larger *ca* 2.5 kcal/mol increases in Δ*G*_OMP_^†^ for reactions with rate determining chemical decarboxylation. By comparison the D37A and T100′G substitutions result in 3–4 kcal/mol increases Δ*G*_FOMP_^†^ due to increases in the barrier to *k*_c_ for the protein conformational change.^215^

The T100′G substitution causes a 3.5 kcal/mol destabilization of the active *Sc*OMPDC dimer relative to the inactive monomer, while the T100′A substitution has no detectable effect on dimer stabilility.^208,215^ The T100′ side chain sits at the dimer interface, close to the N-terminal end of an α-helix (G98′–S106′).^172^ Internal Gly side chains are known to destabilize α-helices relative to Ala,^216,217^ and it was proposed that helix destabilization for the T100′G variant destabilizes the OMPDC dimer relative to monomer by impairing intersubunit contacts at the dimer interface (Figure 37).

**Figure 37:**
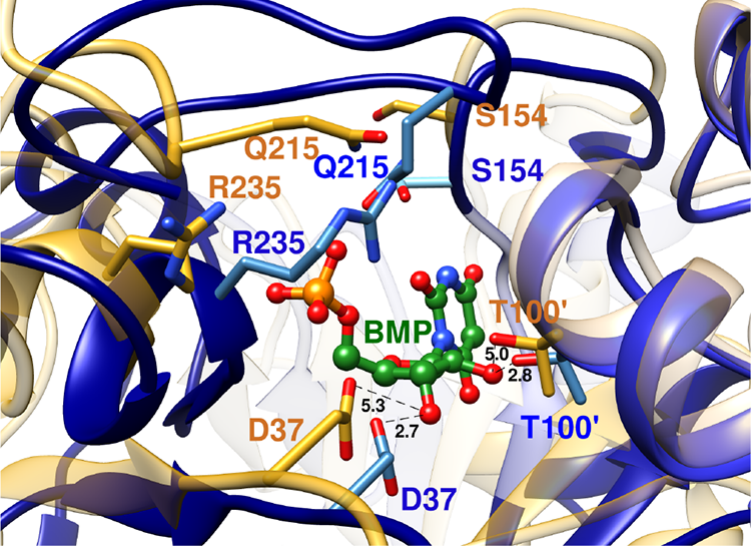
Superimposition of representations of X-ray crystal structures of the unliganded open form of *Sc*OMPDC (gold, PDB 1DQW) and of the closed enzyme (blue, PDB 1DQX) complexed to BMP. The figure illustrates the movement of the D37 and T100′ side chains toward BMP due to ligand-driven ligand movement of protein domains. The side chains lie 5.3 Å and 5.0 Å, respectively, from the C-3′-OH and C-2′-OH ribosyl-OH at the hypothetical BMP ligand that is placed at the same position as BMP bound to the closed enzyme. The distances decrease to 2.7 Å and 2.8 Å, respectively, at the *Sc*OMPDC•BMP complex for the closed enzyme. Reproduced with permission from ref 88. Copyright 2021 American Chemical Society.

#### 5.11.2 Enzyme-Activation by Protein Ribosyl Hydroxyl Interactions

Figure 37 shows that binding of BMP to *Sc*OMPDC is accompanied by movement of D37 and T100′ side chains toward the ligand ribosyl hydroxyls. This is due to domain motion that occurs along a hinge that bisects the enzyme active site and which is driven by ribosyl hydroxyl binding energy that stabilizes the active closed form of *Sc*OMPDC.^218^ We proposed that this binding energy activates *Sc*OMPDC for catalysis of decarboxylation at the pyrimidine ring. Chart 1 compares the activation of wild-type *Sc*OMPDC-catalyzed decarboxylation of the substrate fragment fluoroorotate (**FO**) by phosphite dianion and by sugar phosphates with activation of D37 and T100′ variants, where the D37 and T100′ side chains interact, respectively, with the C-3′ and C-2′ ribosyl-OH (Figure 37).^88,214^ Chart 1 provides support for the following conclusions.

**Chart 1:**
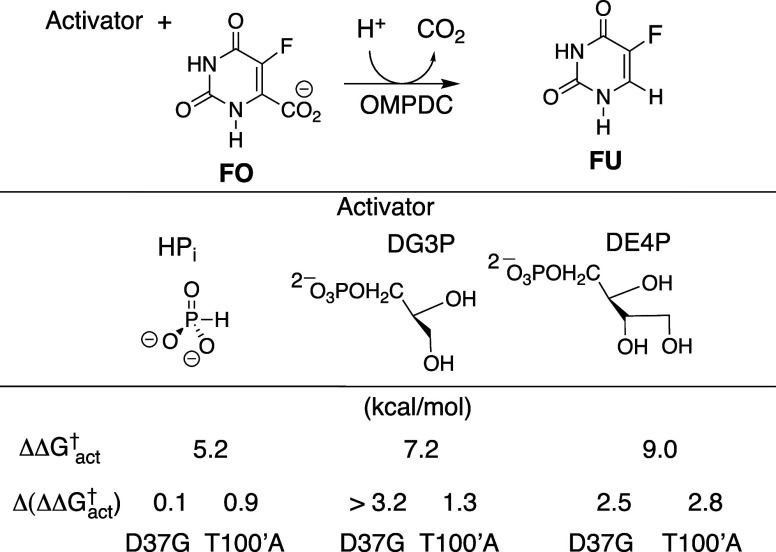
Effect [Δ­(ΔΔ*G*_act_^†^)] of D37G and T100′A Substitutions on ΔΔ*G*_act_^†^ for *Sc*OMPDC-Catalyzed Decarboxylation of FO Activated by Phosphite and Sugar Phosphate Dianions: ΔΔ*G*_act_^†^ Is Calculated from the Ratio of Rate Constants for the Activated and Unactivated Reactions of the Truncated Substrate FO.^88^

(1) The D37G substitution eliminates interactions with the −OH at d-glycerol 3-phosphate (DG3P) and d-erythrose 4-phosphate (DE4P) located at positions sterically equivalent to the C-3′ ribosyl-OH of OMP (Figure 37). The substitution results in significant >3.2 and 2.5 kcal/mol decreases, respectively, in ΔΔ*G* ^†^ for activation of wild-type *Sc*OMPDC-catalyzed decarboxylation of the truncated substrate **FO** by DG3P and DE4P.^88^ This provides support for the conclusion that the interactions between D37 and the C-3′-ribosyl hydroxyl activate OMPDC for catalysis of decarboxylation at the pyrimidine ring.

(2) The T100A substitution eliminates the interaction between the hydroxyl at DE4P that sits at the position sterically equivalent to the C-2′ ribosyl-OH of OMP, but the T100′ side chain should not interact with the −OH at DG3P. The T100′A substitution results in different 2.8 and 1.3 kcal/mol decreases, respectively, in ΔΔ*G* ^†^ for activation of wild-type *Sc*OMPDC-catalyzed decarboxylation of **FO** by DE4P and DG3P, because only the interaction of an −OH at DE4P is eliminated by this substitution.^88^ This provides support for the proposal that the interactions between D37 and the C-2′-ribosyl hydroxyl activate OMPDC for catalysis of decarboxylation at the pyrimidine ring.

### 5.12 Side Chain Interactions that Stabilize the Reaction Intermediate

The prime imperative for catalysis by OMPDC is to reduce the large thermodynamic barrier for loss of CO_2_ to form the vinyl carbanion reaction intermediate.^170,174,219^ A comparison of the equilibrium constants (*K*_eq_)_enz_ = 1.2 × 10^–12^ and *K*_CH_/*K*_RNH3_ = 2 × 10^–22^ for vinyl carbanion formation at *Sc*OMPDC (Scheme 17A) and in water (Scheme 17B, *K*_RNH3_ = 10^–7^ M)^182^ shows that the enzyme effects a ≈ (5 × 10^9^)-fold increase in the equilibrium constant for proton transfer from C-6 of orotidine to an alkylamine base of p*K*_a_ = 7. This corresponds to a 13 kcal/mol stabilization of the vinyl carbanion by interactions with the protein catalyst.^182^

**Scheme 17:**
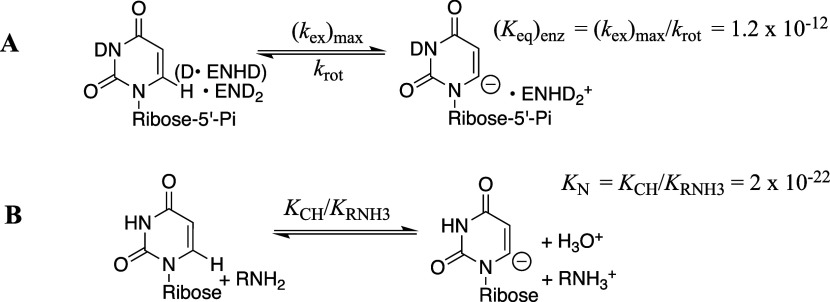
(A) The Pseudo-Equilibrium for Formation of the C-6 Vinyl Carbanion Intermediate of the *Sc*OMPDC-Catalyzed D-Exchange Reaction of UMP, Where (*k*_ex_)_max_ Is for Reaction at Saturating UMP and *k*_rot_ Is for Bond Rotation at the Cationic Lysine Side Chain that Exchanges H for D;^182^ (B) Equilibrium for Nonenzymatic Deprotonation of UMP by an Amine Base of *K*_RNH3_ = 10^–7^ M

OM-MM simulations of the decarboxylation of OMP bound to *Mt*OMPDC provide a free energy barrier for decarboxylation of 14.9 kcal/mol, that is in fair agreement with the experimental estimate of 16.4 kcal/mol.^24^ These calculations use the crystal structure of *Mt*OMPDC complexed to the BMP (**E**_**C**_, Scheme 1) to model the decarboxylation reaction barrier. The results provide support for the conclusion that catalysis by the closed enzyme **E**_**C**_ (Scheme 1) is due entirely to specific transition-state stabilization at an enzyme active site that provides a template for the decarboxylation reaction transition state.

X-ray crystal structure of *Sc*OMPDC complexed to the intermediate analog BMP (Figure 28) shows that the inhibitor sits close to a cluster of charged residues (K59, D91, K93, and D96′), where the K93 side chain cation has been proposed to form a cation–anion pair that stabilizes the vinyl carbanion.^172^ No activity for decarboxylation of OMP was detected for D91A, K93A, and D96′A variants of *Sc*OMPDC,^220^ so that each of these amino acids is essential for the proper enzyme function.^54^ Partial function is observed for the K59A variant where there is a 10^5^-fold falloff in *k*_cat_/*K*_m_ for decarboxylation of OMP.

A simple proposal is that the interaction between the cationic K93 side chain and the vinyl carbanion provides a 13 kcal/mol stabilization of the enzyme-bound intermediate. A comparison of the values of *K*_m_ for decarboxylation of OMP (*K*_m_ ≥ *K*_d_)^208^ and of *K*_d_ for inhibition of decarboxylation by UMP shows that the Michaelis complex to OMP is stabilized by *ca* 3 kcal/mol by interactions with the OMP carboxylate.^208^ We speculate that the network of interacting K59, D91, K93, and D96′ side chains optimizes the difference in the stabilization of the substrate carboxylate anion and the vinyl carbanion reaction intermediate by interactions with the K93 side chain cation.

The D91, K93 and D96 side chains lie in a (DXKXXD) motif reported to be present at the core of OMPDC from all organisms.^179^ This highly conserved core is reported to have “*undergone a variety of changes in subunit size or fusion to other protein domains, such as orotate phosphoribosytransferase, during evolution in different kingdoms*”.^179^ We propose that conservation of the DXKXXD motif reflects the still unknown role of these side chains in stabilization of the vinyl carbanion reaction intermediate. The many other modifications in protein structure during enzyme evolution were then directed toward adjusting the structure of the Michaelis complex to provide for optimal transition state stabilization by interactions of other protein side chains. The positioning of the (DXKXXD) side chains at the Michaelis complex to OMP is likely different from that observed for X-ray crystal structures of OMPDC complexed to a transition state analog or reaction product. The rapid breakdown of the E•OMP complex to form UMP, with a halftime of *ca* 10 ms at room temperature, provides an impediment to the determination of its structure.

Human OMPDC has been purified as the C-terminal domain of a UMP synthase multienzyme complex.^175^ X-ray structures were reported for the Michaelis complex to OMP in the variant (K314 KAcK) of human OMPDC where the lysine side chain from the DXKXXD motif has been acetylated.^176^ The X-ray crystal structure for this Michaelis complex shows migration of a proton from the wild-type Lys-NH_3_^+^ to the −COO^–^ of a critical Asp from the DXKXXD motif, with stabilization of the protonated Asp side chain by a hydrogen bond to the OMP carboxylate. The activity of the K314 KAcK variant is only 0.06% of wild-type human OMPDC. It is likely that this reduction in activity reflects a change in reaction mechanism associated with the migration of a proton from the Lys to the Asp side chain. The mechanistic model described in this review is based mainly on the results of experiments on OMPDC from yeast and predicts that the fully ionized OMP carboxylate undergoes direct decarboxylation to form O=C=O product that is strongly stabilized by interactions with hydrophobic active-site side chains. We see no obvious catalytic role for partial protonation of the substrate by an active-site Asp side chain.

### 5.13 Summary

A comparison of the second-order rate constants *k*_cat_/*K*_m_ for OMPDC-catalyzed decarboxylation of OMP, EO and orotate provides values of 12 and 10 kcal/mol, respectively, for transition state stabilization by the nonreacting phosphodianion and ribosyl fragments of OMP.^10,214^ The binding energy for these substrate fragments is utilized to stabilize a protein conformation where the enzyme active site serves as tight-binding template for formation of the decarboxylation reaction transition state.

Interactions between the substrate dianion or the activator phosphite dianion and the Q215, Y217, R235 side chains provide, respectively, a 12 kcal/mol stabilization of the transition state for *Sc*OMPDC-catalyzed decarboxylation of OMP and an 8 kcal/mol stabilization of the transition state for dianion-activated decarboxylation of EO.^10,13^ The 8 kcal/mol interaction represents the binding energy utilized to hold *Sc*OMPDC in a closed conformation, where the dianion gripper loop and the umbrella that covers the substrate pyrimidine ring sequester OMP from interaction with bulk solvent (Figure 29).

The deconstruction of the dianion gripper loop by sequential single substitutions of S154, Q215, Y217, and R235 side chains (Figure 29) uncovers systematic variations in the effect of individual side chain substitution on the barrier Δ*G*_OMP_^†^ for substrate decarboxylation. The effects of single Q215A, Y217F, and R235A substitutions at wild-type OMPDC on Δ*G*_OMP_^†^ sum to a value approximately equal to the total effect of the three side chains on transition state stability and therefore are close to the intrinsic side chain interactions. By comparison, the sum of the effects of single Q215A, Y217F, and R235A substitutions on Δ*G*_OMP_^†^ at OMPDC variants that lack the remaining two gripper side chains exceeds the total effect of the three gripper side chains on the stability of the wild-type transition state, because the substitutions now cause a weakening in other protein interactions that stabilize this transition state (section 4.8). Finally, substitution of S154 and two additional gripper side chains severely erodes the function of the dianion gripper loop, so that the sum of effects of the final single Q215A, Y217F, and R235A substitutions on Δ*G*_OMP_^†^ is much smaller than the total effect of the three gripper side chains on transition state stability.

Interactions between the substrate ribosyl ring hydroxyls and the D37 and T100′ side chains are utilized to stabilize the catalytically active domain-closed form of *Sc*OMPDC (Figure 37) and account for a large fraction of the *ca* 10 kcal/mol ribosyl ring-stabilization of the decarboxylation reaction transition state. The binding interactions of D37 and T100′ side chains with truncated forms of ribose 5′-phosphate activate *Sc*OMPDC for catalysis of decarboxylation of 5-fluoroorotate.^88^ This provides direct evidence that these side chain interactions drive an enzyme-activating protein conformational change.

Interactions between *Sc*OMPDC and nascent CO_2_ product are estimated to provide a *ca* 10 kcal/mol stabilization of the decarboxylation reaction transition state.^204^ This accounts for much of the difference in the activity of *Sc*OMPDC for catalysis of decarboxylation of OMP and the deuterium exchange reaction of FUMP in D_2_O.^182,204^ The loss of the strong interaction of CO_2_ product at the transition state for the *Sc*OMPDC-catalyzed deuterium exchange reaction provides a rationalization for the difference in the effect of dianion gripper side chain substitutions on the kinetic parameters for the *Sc*OMPDC-catalyzed decarboxylation and deuterium exchange reactions (Table 3).^204^

The large rate acceleration for *Sc*OMPDC is obtained by combining two important properties for proteins in a single enzyme.^31^ First, the active closed form of *Sc*OMPDC (**E**_**C**_) provides a rigid protein cage for the substrate that is complementary to the enzymatic transition state. This Michaelis complex has been used to model the rate acceleration for OMPDC. Second, the inactive unliganded protein uses protein–ligand interactions to drive thermodynamically demanding changes in protein conformation to form the catalytically active closed enzyme (Scheme 1), where the active-site side chains are positioned to provide optimal transition state stabilization. These binding interactions are not expressed in the value of *K*_m_ for formation of the Michaelis complex but rather are utilized to stabilize the active protein cage that shows a tight fit for the decarboxylation reaction transition state. The evolution of rigid precursor enzymes to forms that undergo substrate-driven conformational changes from flexible inactive forms to catalytically active cages for the enzyme-bound substrate requires the erosion of the structure of the active stiff precursor and the selection of the caged complex generated by the protein confomational change.^27^

The requirement for transition-state complementarity promotes natural selection of active-site protein cages that provide specificity for binding transition states with a much higher affinity than substrate. This specificity is reflected for the active **E**_**C**_ form of *Sc*OMPDC by (i) The *ca* 13 kcal/mol stabilization of the vinyl carbanion reaction intermediate relative to the substrate OMP that is expressed at the late, product-like, transition state for enzyme-catalyzed substrate decarboxylation.^182^ The conserved (DXKXXD) motif is proposed to have evolved to optimize stabilizing interactions between a cationic lysine side chain and the carbanion intermediate. (ii) The *ca* 10 kcal/mol stabilization of the late, product-like transition state for decarboxylation of OMP by interactions of hydrophobic protein side chains at the pyrimidine ring binding site with the nascent CO_2_ product.^182^ The mechanism by which such active catalytic cages evolve alongside the erosion of the structure of the rigid parent enzyme is unclear.^27^

## 6 Protein Structure–Function Relationships

Enzymologists failed for many years to recognize the role of induced-fit type conformational changes in catalysis by nature’s most proficient enzymes. Furthermore, investigations on the mechanisms for enzyme activation by protein conformational changes are in their infancy and there has been little thought given to the development of strategies for the incorporation of these activating protein conformational changes into protocol for the *de novo* design of protein catalysts. We hope that the work described in this review will provide a modest foundation for future studies.

The induced fit model from Scheme 1 provides a straightforward rationalization for the large body of experimental and computational data discussed in this manuscript. We note two particularly striking results that are difficult or impossible to rationalize by classical models for enzymatic catalysis.

(1) The L230A substitution at TIM causes a shocking 20-fold increase in *k*_cat_/*K*_m_ for the TIM-catalyzed reactions of the truncated substrate [1-^13^C]-GA, and a small decrease in *k*_cat_/*K*_m_ for the catalyzed reaction of whole triosephosphate substrates.^95^ This is consistent with the notion that TIM has evolved to exist in both stable inactive open forms (**E**_**O**_) and in unstable, active closed forms (**E**_**C**_), and that this change in protein conformation is driven by the utilization of phosphodianion binding energy.^27^ This model predicts the existence for some enzymes of protein structural elements that destabilize **E**_**C**_ relative to **E**_**O**_ (Scheme 1B) so that side chain substitutions which decrease the destabilization of **E**_**C**_ will cause a specific increase in the kinetic parameters for the catalyzed reactions of phosphodianion truncated substrates. In the case of TIM this barrier is partly due to the requirement for desolvation of the basic E165 side chains and is proposed to be reduced by substitutions that favor solvation of this base.^93–95^

(2) Interactions between OMPDC and the OMP substrate phosphodianion are established by three gripper side chains from OMPDC that are distant from the site of pyrimidine ring decarboxylation. The triple Q215A/Y217F/R235A side chain substitution at *Sc*OMPDC causes very different 1.3 and 12 kcal/mol changes, respectively, in the activation barrier for enzyme-catalyzed decarboxylation of the phosphodianion truncated substrate EO and the specific substrate OMP.^191^ The former small effect shows that there are minimal direct stabilizing side chain interactions with the pyrimidine ring. The latter large effect is consistent with a strong stabilization of the active enzyme **E**_**C**_ by interactions with the substrate phosphodianion (Figure 29). The results provide support for a model where the rigid protein core, that provides efficient catalysis of decarboxylation of a neutral substrate such as EO, evolved first. The structure of this core was then modified during evolution by appending a dianion gripper loop that provides specificity for catalysis of decarboxylation of phosphorylated substrate OMP.

There is a need to strike a balance between contrasting hypotheses. First, we predict that the activating dianion driven conformational changes for TIM, GPDH, and OMPDC and other enzymes share common properties. Second, we expect that the different imperatives for catalysis of proton transfer, hydride transfer and decarboxylation reactions will be reflected by differences in the dianion-driven motions observed for these different enzymes.

We note the following similarities in the substrate driven protein conformational changes for TIM, GPDH and OMPDC. (i) Each enzyme utilizes dianion binding interactions to drive a protein conformational changed that creates an active-site cage for the substrate. (ii) The dianion driven protein motions either align flexible loops over the substrate phosphodianion or close protein domains around the substrate. (iii) The formation of caged Michaelis complexes excludes bulk water from the enzyme active site. (iv) The formation of these caged complexes is accompanied by movement of catalytic side chains into positions that provide optimal stabilization of the enzymatic transition state. (v) TIM (K12), GPDH (R269), and OMPDC (R235) each has a key cationic side chain that is positioned at the closed enzyme to interact with the substrate dianion. (vi) Each of these side chain cations acts to hold their respective enzyme in the active closed conformation. (vii) Neutral for cationic side chain substitutions result in a large destabilization of the enzymatic transition state: K12G substitution at TIM, 8 kcal/mol;^55^ R269A substitution at GPDH, 9 kcal/mol;^141^ R235A at OMPDC, (6 kcal/mol).^186,187^ (viii) Each of these variants show efficient rescue of wild-type activity by the small molecule analog of the excised side chain; Arg, Gua^+^, and Lys, EtNH_3_^+^.^56,143,187^

A detailed examination of these conformational changes reveals elements tailored to optimize the specific catalyzed reaction. TIM catalyzes the reaction of the compact triosephosphate substrate, where the substrate dianion provides nearly the entire observed transition state binding energy.^52^ The TIM barrel scaffold is organized into flexible loops that interact with the substrate dianion, and rigid loops that are connected by inter- and intraloop hydrogen bonds and whose position remains fixed during the protein conformational change. The dianion binding energy is utilized in the construction of a protein cage that provides for a strong increase in the driving force for substrate deprotonation. The protein conformational change is dominated by dianion-driven movement of loops 6 and 7 which entrap the substrate in a protein cage, while at the same time driving desolvation the active-site base by moving the base into a hydrophobic clamp. This desolvation strongly increases the driving force for substrate deprotonation by the catalytic base.

The substrate phosphodianion provides similar 12 kcal/mol stabilization of the transition states for OMPDC-catalyzed decarboxylation and TIM-catalyzed isomerization, but this corresponds to a relatively small fraction of the total 31 kcal/mol stabilization of the transition state for OMPDC-catalyzed decarboxylation. The phosphodianion binding energy and the 10 kcal/mol binding energy from substrate ribosyl hydroxyl groups is utilized to drive expansive protein conformational changes that activate OMPDC for catalysis of decarboxylation of OMP. Enzyme activation is partly or entirely due to the stabilization of a caged Michaelis complex for OMP that provides for a large stabilization of the C-5 vinyl carbanion reaction intermediate relative to substrate OMP and product UMP. This stabilization is proposed to be due to a strong interaction between negative charge at the reaction intermediate and the K93 side chain cation from a highly conserved (DXKXXD) motif.^179^

The substrate phosphodianion also provides a 12 kcal/mol stabilization of the transition state for GPDH-catalyzed hydride transfer to DHAP. The large dianion binding energy is not strictly required for efficient catalysis, because of the large binding energy available from the NAD^+^ cofactor. The primary role for utilization of the substrate binding energy is to drive a protein conformational change which organizes the scattered apoenzyme active-site side chains into positions that provide optimal substrate reactivity and stabilization of the transition state for enzyme-catalyzed hydride transfer. The expansive conformational change moves the [292-LNGQKL-297] loop folds over the mouth of the enzyme active site at the ternary E•NAD^+^•DHAP complex (Figure 11). This motion positions the Q295 and K296 loop side chains to interact, respectively, with the DHAP phosphodianion and the cofactor adenine ring. It is consistent with the notion that activation of *hl*GPDH by the dianion fragment of substrate DHAP and by the AMP fragment of cofactor NAD^+^ is due to a coordinated protein conformational change that is driven by interactions with fragments contributed by each substrate.^20^

## 7 Concluding Remarks

Computational chemists are now able to start with the structures of enzyme Michaelis complexes and use these to model the activation barriers to many enzyme-catalyzed reactions. This has created in some the impression that there is now little scope for fundamental advances in our understanding of enzymatic reaction mechanisms. The response of mechanistic enzymologists is to cite the limited success of efforts at *de novo* design of protein catalysts as justification for the existence of previously unrecognized models for enzymatic catalysis.^154,221^ One prominent enzymologist responded in 2009 by presenting “*A 21st century revisionist’s view at a turning point in enzymology”*,^154^ and 16 years later by moving toward “*A Foundational Shift in Models for Enzyme Function”*.^221^ We encourage readers to examine and critically assess these papers and other models that propose to establish links between protein motions and enzyme catalytic efficiency.^222,223^

Protocols for the *de novo* design of active protein catalysts that ignore the catalytic role of ligand-driven conformational changes are unlikely to reproduce the catalytic power of such proficient enzymes as TIM and OMPDC. Current design strategies focus on identifying structures for proteins that are catalytically active in their unliganded form and to treat substrate binding as a passive event. In fact, many of Nature’s most proficient catalysts are inactive in their unbound form; substrate binding is the first step in a process that generates the active catalyst. We suspect that the field of *de novo* enzyme design is not yet sufficiently developed to enable design of protein catalysts that conform to the induced-fit model but would be thrilled if this view turns out to be overly pessimistic.

The recognition of the importance of induced-fit protein conformational changes has led to the identification of elements of enzyme architecture that enable efficient catalysis through coordinated, substrate-driven, movement of protein side chains from scattered positions in the free enzyme to their catalytic positions at active sites that are complementary to the reaction transition state. We are more confident in predicting that many more important roles for catalytic loops and related protein structural elements remain to be identified in studies on the ligand-driven protein conformational changes observed for other enzymes. An expansion in our understanding of the role of activating conformational changes in enzyme catalysis can only help facilitate their *de novo* design by protein engineers.

An important step in the development of methods to study the catalytic role of protein conformational changes and other aspects of enzymatic reaction mechanisms is to define the imperatives for effective catalysis. For example, effective catalysis by TIM and OMPDC requires that these enzymes reduce the large thermodynamic barriers to formation of their respective carbanion reaction intermediates. This was the basis for our hypotheses, largely confirmed by the experimental results described here, that TIM and OMPDC utilize protein binding energy to generate protein cages that provide strong stabilization of carbanion reaction intermediates. This hypothesis provides a starting point for studies on the many other enzymes that catalyze proton transfer and decarboxylation reactions through unstable carbanion reaction intermediates and which undergo substrate driven conformational changes to form caged complexes for substrates.

There has been less attention paid to the formal role of protein conformational changes in stabilization of protein cages that enable formation of carbocation reaction intermediates that show short or negligible lifetimes in water.^224–230^ We predict that the cages for the intermediates of enzyme-catalyzed carbocation cascade reactions that lead to the biosynthesis of natural product are stabilized by binding interactions with nonreacting substrate pyrophosphate groups.^227–229^ We look forward to reading the results of future structure–function studies on the relationship between protein cage structure and the stability of enzyme bound carbanion and carbocation reaction intermediate.

The importance of induced-fit protein conformational changes in enzyme catalysis raises interesting questions about the timing and the imperatives for the appearance of these conformational changes during evolution, because it is not obvious how natural selection of protein catalysts of increasing proficiency resulted in the appearance of catalysts that are inactive in their unliganded form.^27^ Insight into the answer to this question might be obtained by a close examination of induced fit conformational changes observed for members of families or superfamilies of enzyme catalysts.^231–233^
